# Hit-to-Lead Development of the Chamigrane Endoperoxide Merulin A for the Treatment of African Sleeping Sickness

**DOI:** 10.1371/journal.pone.0046172

**Published:** 2012-09-27

**Authors:** Gabriel Navarro, Supchar Chokpaiboon, Geraldine De Muylder, Walter M. Bray, Sean C. Nisam, James H. McKerrow, Khanitha Pudhom, Roger G. Linington

**Affiliations:** 1 Department of Chemistry and Biochemistry, University of California Santa Cruz, Santa Cruz, California, United States of America; 2 Department of Chemistry, Faculty of Science, Chulalongkorn University, Bangkok, Thailand; 3 Sandler Center for Basic Research in Parasitic Disease, University of California San Francisco, San Francisco, California, United States of America; 4 Chemical Screening Center, University of California Santa Cruz, Santa Cruz, California, United States of America; INSERM U1094, University of Limoges School of Medicine, France

## Abstract

**Background:**

Human African trypanosomiasis (HAT) is an infectious disease with a large global health burden occurring primarily in Central and Eastern Africa. Most current treatments have poor blood brain barrier (BBB) penetration, which prevent them from targeting the most lethal stage of the infection. In addition, current therapeutics suffer from a variety of limitations ranging from serious side effects to difficulties with treatment administration. Therefore it is of crucial importance to find new treatments that are safe, affordable, and effective against both sub-species of *Trypanosoma brucei*.

**Methods:**

Semi-synthetic derivatization of the fungally-derived natural product merulin A (**1**) has led to the discovery of new development candidates for the protozoan parasite *T. brucei*, the causative agent of HAT. Creation of an initial SAR library based around the merulin scaffold revealed several key features required for activity, including the endoperoxide bridge, as well as one position suitable for further derivatization. Subsequent synthesis of a 20-membered analogue library, guided by the addition of acyl groups that improve the drug-like properties of the merulin A core, resulted in the development of compound **12** with an IC_50_ of 60 nM against *T. brucei*, and a selectivity index greater than 300-fold against HeLa and immortalized glial cells.

**Significance:**

We report the semi-synthetic optimization of the merulin class of endoperoxide natural products as development candidates against *T. brucei*. We have identified compounds with low nM antiparasitic activities and high selectivity indices against HeLa cells. These compounds can be produced economically in large quantities via a one step derivatization from the microbial fermentation broth isolate, making them encouraging lead candidates for further development.

## Introduction

Human African trypanosomiasis (HAT) is a vector-borne disease with a large global health burden. Transmitted by the tsetse fly vector, the flagellated protozoan *Trypanosoma brucei* subspecies are the etiologic agents of HAT. *T. b. gambiense*, a more chronic infection, is prevalent in West and Central Africa, while *T. b. rhodensiense*, a more acute and virulent infection, occurs primarily in East and Southern Africa. Infection with either subspecies manifests itself in two stages. In the first stage, the parasite resides and proliferates in the hemolymphatic system. This stage is characterized by fever, headaches, joint pain, and itching. In the second stage, the parasite crosses the blood brain barrier (BBB) and infects the central nervous system (CNS). This stage is characterized by confusion, reduced coordination, and disruption of the sleep cycle. If a second stage infection is left untreated the disease becomes lethal.

Current treatments are poor and suffer from a variety of limitations ranging from severe side effects to difficulties with treatment administration (Table 1). These limitations reduce the effectiveness of current treatments in developing countries and result in poor patient compliance. In particular, the side effects for the treatment of the second stage infection (CNS infection) with melarsoprol are very severe and include an estimated three to ten percent mortality rate due to drug-associated toxicity [1]. The use of eflornithine to treat second stage HAT infection, while safer than melarsoprol, still suffers from the following common side effects: fever, unusual bleeding, weakness, diarrhea, nausea, stomach pain, and vomiting. Therefore it is of crucial importance to find new treatments to combat HAT and overcome the problems with current therapeutics.

**Table 1:** Available therapeutics for HAT.

Drug	Parasite Specificity	Stage Specificity	Limitations
Suramin	*Rhodensiense*	first-stage	Efficacy, parenteral
Melarsoprol	*gambiense* & *rhodensiense*	second-stage	Safety, parenteral
Pentamidine	*Gambiense*	first-stage	Resistance, compliance, parenteral
Eflornithine	*Gambiense*	second-stage	Cost, parenteral

Due to the lack of financial incentive for major pharmaceutical companies to invest in tropical disease medicine, the development of new drugs to treat neglected diseases, such as HAT, has progressed slowly over the past 30 years [2]. New efforts in this area are increasingly driven by public private partnerships (PPP), which typically couple academic research programs with industrial partners to fill technical gaps in the drug development pipeline that are outside the purview of most academic programs. These PPPs can be funded from a diverse array of sources, including federal grants, philanthropic funding (e.g. the Bill and Melinda Gates Foundation), and ‘*pro bono*’ contributions from the pharmaceutical companies (e.g. The Novartis Vaccines Institute for Global Health and the Global Health Discovery Unit at GlaxoSmithKline). Representative examples of PPP-based drug development programs include the Drugs for Neglected Disease *initiative* (DND*i* - Geneva), Institute for One World Health (IOWH - San Francisco CA), Infectious Disease Research Institute (Seattle WA), African Programme for Onchocerciasis Control (APOC), and Onchocerciasis Elimination Programme in the Americas (OEPA). Highlights of current PPP successes in HAT drug discovery and development include DND*i*'s oxaborole SCYX-7158 (Sandler Center of the University of California San Francisco/SCYNEXIS - Phase I study) and fexinidazole (Sanofi/Swiss Tropical and Public Health Institute – Phase II/III study) [3], [4]. Both of these compounds have reached clinical trials and appear to be promising treatments for HAT.

Potential development candidates must meet strict target product profiles (TPP) to be considered for further development by PPP organizations. For HAT, the most widely recognized TPP is published by DND*i* (Table 2). While many of these criteria can only be evaluated using expensive and low-throughput *in vivo* animal models, some early *in silico* and *in vitro* assays can be conducted as indicators of the suitability of a given compound to meet the TPP requirements outlined by DND*i*. These indicators, which are conducted in our laboratories as a component of our broader goal to develop new treatments for HAT and other neglected infectious diseases, include *in vitro* antiparasitic screening against protozoan parasites, human cell line screening for evaluating mammalian cell cytotoxicity and potential CNS toxicity, *in vitro* evaluation of serum stability to measure compound half-life, *in silico* prediction of BBB permeation and human intestinal absorption, and a focus on microbially-derived lead compounds to reduce the cost of producing affordable drugs. Together, these factors combine to provide a mechanism to select lead compounds of the highest priority for hit-to-lead development against global health targets such as HAT.

**Table 2:** DNDi target product profile for HAT.

Ideal	Acceptable
Effective against stage 1 and 2	Effective against stage 1+2 (used stage 2 only)
Broad Spectrum (*gambiense* and *rhodesiense*)	Efficacy against *gambiense* only
Clinical efficacy >95% at 18 months follow up	To be determined by expert consultation
Effective in melarsoprol refractory patients	
<0.1% drug related mortality	<1% drug related mortality
Safe also during pregnancy, for breastfeeding women and children	
Adult and paediatric formulations	
No monitoring for AEs	Weekly simple lab testing (field testing)
<7 days p.o. once daily (DOT)	10 days p.o. (up to tid)
<7 days i.m. once daily	10 days i.m. once daily
Stability in Zone 4 for >3 years	Stability in Zone 4 for >12 months
Cidal	Static
Multitarget	Unique target (but not uptake via P2-transporter only)
<30 €/course* (only drug cost)	<100 €*/course
	<200 €*/course ok if very good on other criteria

* It is expected that donor agencies will pay this, not patients. Considering that some 20,000–50,000 patients per year might require treatment, this is still realistic.

**Figure 1:**
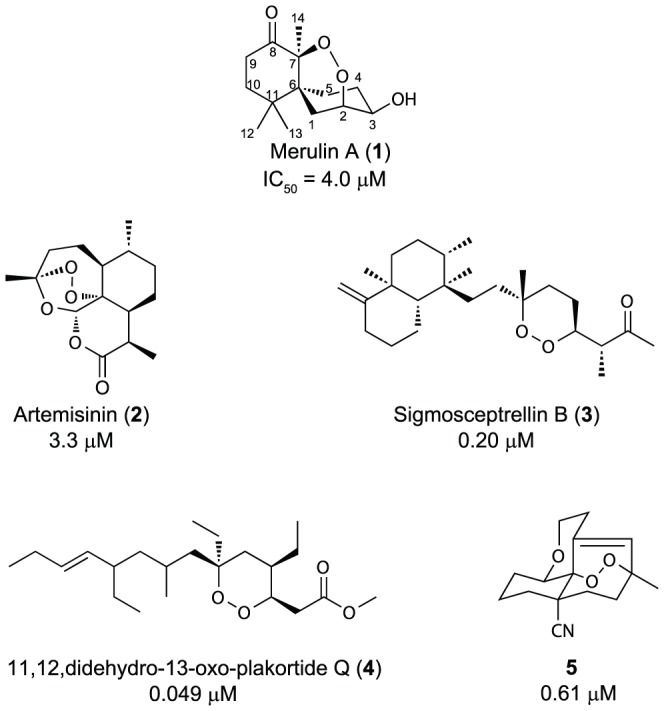
Reported anti-trypanosomal endoperoxides.

In recent years, several publications have reported the discovery of cyclic endoperoxide scaffolds with anti-trypanosomal activities (Figure 1). Among these, artemisinin (**2**) [5], an endoperoxide-containing terpene that is currently used as an active ingredient in all front line antimalarial combination therapies, is the most well-known. However, the relatively weak activity of **2** against *T. brucei* and its high production cost [6] make it a poor candidate for further development. A number of other endoperoxide-containing natural products, including sigmosceptrellin B (**3**) [7] and 11,12-didehydro-13-oxo-plakortide Q (**4**) [8], have displayed impressive *in vitro* activities against *T. brucei*. Unfortunately, because these compounds originate from marine sponges, subsequent development has been hampered by a lack of available material. To address this, two independent research groups have recently reported the generation of libraries of synthetic analogues, highlighted by **5**, that show strong efficacy against *T. brucei* with excellent selectivity indices (SI) [9], [10]. However, these approaches require multi-step synthetic routes, and include low yielding key photo-oxidation steps. Therefore a gap still exists in the creation of new approaches for the development of endoperoxide-containing compounds as lead compounds for HAT.

Merulins A–C are a new class of endoperoxide-containing natural products recently isolated in one of our laboratories [11], in high yield (>28 mg/L) from liquid fungal cultures. With a high yielding renewable source and orthogonal functional groups for chemical derivatization, the merulin compound class contains many elements that position it to overcome the obstacles that have hampered previous attempts to develop endoperoxide-based treatments for HAT. The discovery of these new endoperoxide-containing compounds provided motivation to explore their potential for development in this area.

## Results and Discussion

### Synthesis and *in vitro* activity

**Figure 2:**
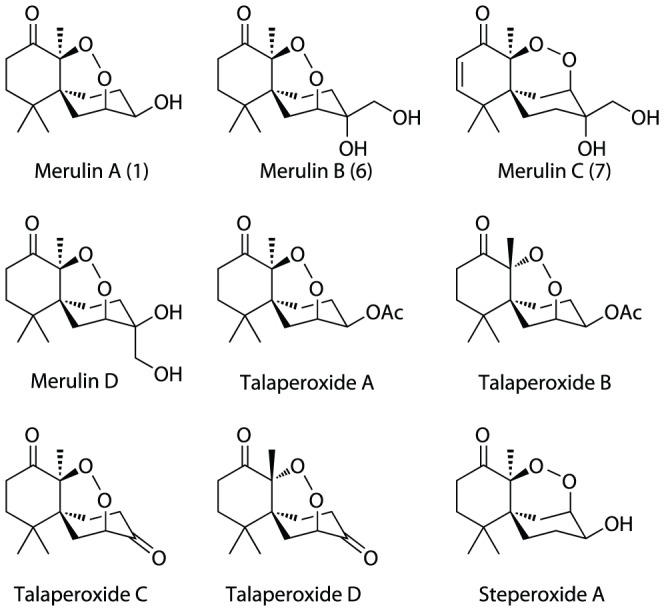
Natural product analogues of merulin A.

**Figure 3:**
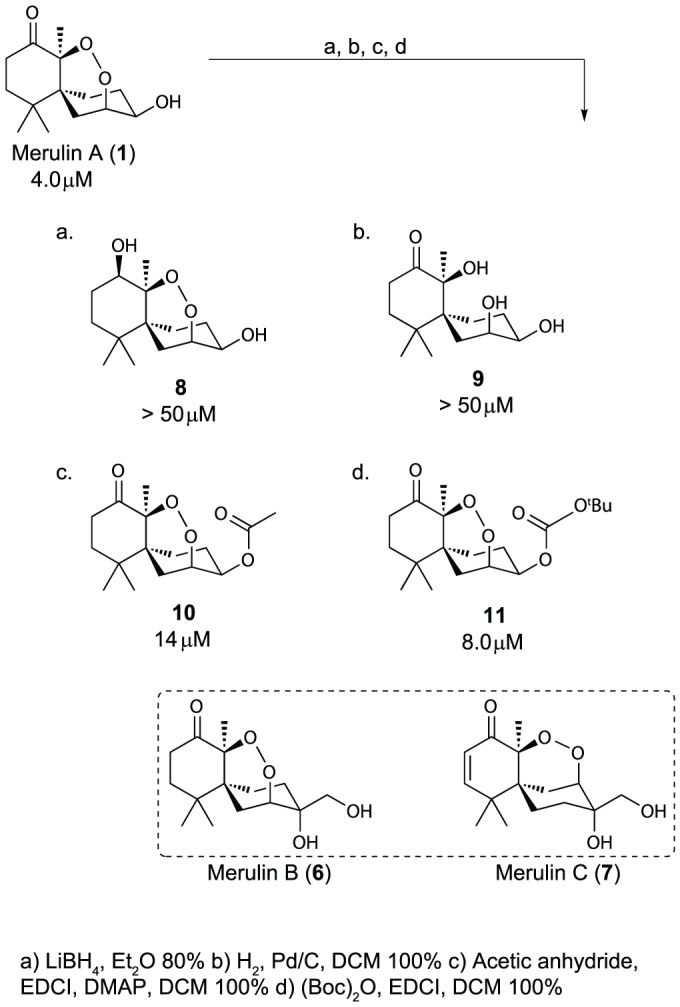
Initial SAR analog library. IC_50_ values against *T. brucei* brucei shown below each structure.

Merulin A was isolated from an endophytic fungus in the family Meruliaceae (Subkingdom Dikarya, Phylum Basidiomycota) and first reported in 2010 [11]. While the original isolation publication reported two additional analogues, other recent studies have expanded the suite of merulin-like compounds to nine (Figure 2) [12]–[14]. Most of these merulin analogues, along with the parent compound merulin A, were reported to possess moderate cytotoxicities against a variety of mammalian cell lines (10–30 µM), but were not tested for antiprotozoal activity. Initial *in vitro* screening of merulins A–C (**1**, **6**, **7**) against the bloodstream form of *T. brucei brucei* in one of our laboratories showed that these compounds possess activities in the low µM range (0.8–8 µM) (Figure 3), and confirmed our initial hypothesis that these compounds could be effective as antiparasitic agents, albeit with weak activity. Screening of these compounds against HeLa cells was in line with published data, indicating cytotoxicity values in the low µM range. Despite the modest potencies and low SIs for these compounds, the presence of the six-membered endoperoxide ring system and a renewable and ready source of supply of the lead compounds prompted us to explore the structure-activity relationship (SAR) characteristics of the merulin A core. A large-scale culture and isolation effort yielded 415 mg of merulin A for semi-synthetic derivatization, as well as small quantities of merulins B and C for biological analysis.

Substantial effort has been invested to determine the mode of action of cyclic endoperoxides as antiparasitics, because of the importance of artemisinin in antimalarial therapy. Despite these efforts, the precise mechanism of action of artemisinin and other endoperoxides remains unknown. It has also proved difficult to generate clearly defined pharmacophore models for this compound series, due to the ambiguity surrounding the molecular target(s) [15], [16]. In designing our analogue series, we elected to focus our attention on the improvement of pharmacokinetic (PK) and pharmacodynamic (PD) properties, using Accelry's Discovery Studio, to address current limitations in *T. brucei* therapeutics. Considering the calculated PK and PD properties for merulin A (Table 3), several of these properties require optimization to meet the TPP outlined by DND*i*. In particular, predicted BBB permeation and hepatotoxicity values (both calculated *in silico*) were outside the desired ranges for lead candidates. Additionally, in order to avoid ambiguous cutoffs presented by Lipinski's rules, we used a recently reported method for qualitative estimate of drug-likeness (QED) to determine favorable physicochemical properties (Table S2) [17]. Not surprisingly, the QED scores for current HAT therapeutics are low (Table 3). By comparison, merulins A–C (**1**, **6**, **7**) display more favorable properties than the current drugs. Most of the proposed semisynthetic analogues in this new library also possessed favorable QED scores. These calculated results provide additional evidence that the merulin core is a reasonable starting point for the creation of new lead compounds against HAT.

**Table 3:** Comparison of computational pharmokinetic and pharmacodynamic properties of HAT therapeutics to merulin A analogues.

	MW	ADMET BBB	ADMET BBB level	ADMET Absorption	ADMET Hepatotoxicity Probability	ADMET PPB Level	ADMET ALogP98	QED (weighted)	*T.b.b* IC_50_ (uM)
Suramin	1296	*n/a*	4	3	0.98	1	4.447	0.016	0.007
Melarsoprol	398	*n/a*	4	0	0.913	0	2.108	0.5556	0.002
Pentamidine	340	*n/a*	4	0	0.764	0	2.658	0.302	0.03
Eflornithine	182	−1.826	3	0	0.066	0	−0.742*	0.390	59.0
Merulin A (**1**)	254	−0.287	2	0	0.574	0	2.436	0.708	4
Merulin B (**6**)	284	−0.827	3	0	0.536	0	1.753	0.690	8
Merulin C (**7**)	282	−0.834	3	0	0.45	0	1.730	0.688	0.8
**12**	416	−0.094	2	0	0.213	1	4.25	0.701	0.06
**30**	483	*n/a*	4	0	0.437	1	4.707	0.587	0.42

MW - Molecular weight; ADMET BBB – log of brain/blood partition coefficient; ADMET BBB Level – Ranking of the LogBB values into the following levels: 0 - Very high (value≥0.8), 1 – High (0.8>value≥0.0), 2 – Medium (0.0>value≥−0.8), 3 – Low (value<−0.8), 4 – Undefined; ADMET Hepatotoxicity Probability; ADMET PPB Level – plasma protein binding level: the lower the number the better; QED - quantitative estimate of drug-likeness: the bigger the number the more drug-like, weighted using QED_w,mo_. [17].

In identifying synthetically accessible sites to generate derivatives of natural product **1**, three positions on the core scaffold (carbonyl C8, alpha-carbon C9 and hydroxyl on C3) were selected as preliminary chemical handles for expanding the SAR model for the merulin series. Initial attempts to derivatize the carbonyl position using Tebbe olefination, Corey-Chaykovsky epoxidation, Takai iodo olefination, and Baeyer-Villiger oxidation proved unsuccessful. However, treatment of **1** with LiBH_4_/THF resulted in the diastereospecific formation of the R-hydroxyl (**8**) as the major diastereomer. Our initial hypothesis suggested that the endoperoxide was an essential structural feature. Therefore we converted merulin A to the corresponding diol (**9**) by opening the endoperoxide ring under hydrogenation conditions. Attempts to derivatize the alpha position using a variety of approaches proved unsuccessful. However, derivatization at the C3 alcohol was readily accomplished in high yield to afford esters **10** and **11**.

Screening of this initial compound series (**8–11**) revealed a number of SAR features that were valuable in the design of the second-generation library (Figure 3). As expected, opening of the endoperoxide ring (**9**) led to complete loss of activity. In addition, reduction of the carbonyl also eliminated activity, precluding us from developing alternative modifications at this site. The three compounds in this initial SAR series that possessed structural differences at the secondary alcohol on C3 (**6**, **10**, and **11**) all displayed similar activities to merulin A, suggesting that this position was suitable for further derivatization, and tolerant to a range of steric and stereoelectronic modifications. However, none of these new derivatives improved potency or selectivity indices over the parent compound. By contrast, merulin C (**7**) displayed significantly improved activity (0.8 µM), suggesting that the potency of this initial scaffold could be improved by structural modification. Merulin C contains variations at the secondary alcohol, the addition of an α,β-unsaturated carbonyl, and an alternative connectivity for the endoperoxide bridge. This combination of structural variations made it impossible to determine which structural components were responsible for the improvement in activity, however, these results provided encouragement for further synthetic development.

**Figure 4:**
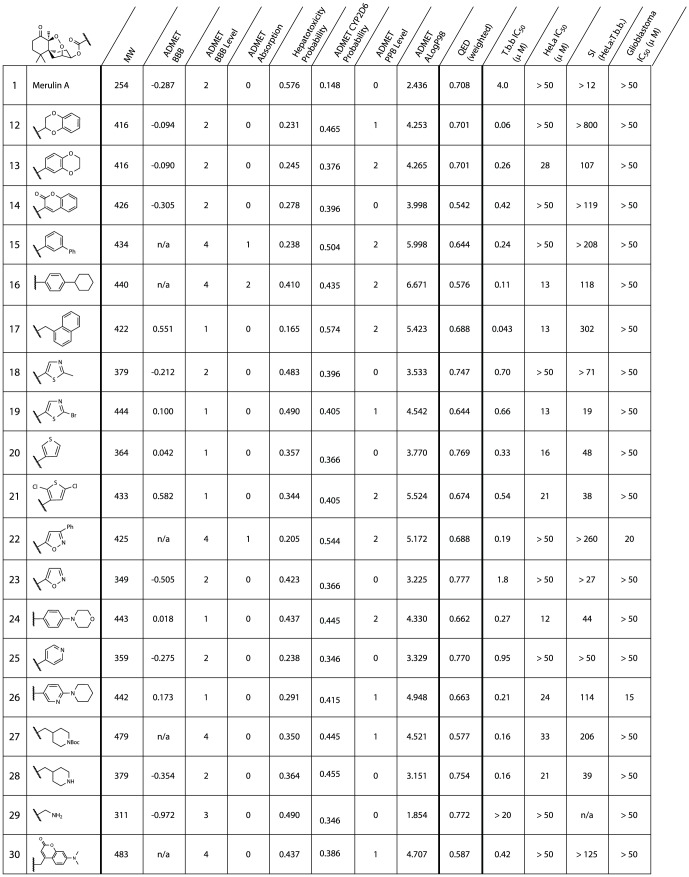
Ester library biological data. MW - Molecular weight; ADMET BBB – log of brain/blood partition coefficient; ADMET BBB Level – Ranking of the LogBB values into the following levels: 0 - Very high (value≥0.8), 1 – High (0.8>value≥0.0), 2 – Medium (0.0>value≥−0.8), 3 – Low (value<−0.8), 4 – Undefined; ADMET Hepatotoxicity Probability; ADMET PPB Level – plasma protein binding level: the lower the number the better; QED - quantitative estimate of drug-likeness: the bigger the number the more drug-like, weighted using QED_w,mo_ [17]. *n.d.* = not determined.

A second-generation library was designed, centered on the derivatization of the secondary alcohol at position C3 in merulin A. This library was designed to improve PK and PD properties, with high priority on calculated BBB permeation, without surpassing the acceptable MW limit (Figure 4). With this in mind we biased the design of our derivatives toward lipophilic molecules with H-bonding capacity, mainly in the form of hetero-aromatic compounds, as there is significant literature relating the importance of lipophilicity and hydrogen bonding to membrane permeability [18]–[21]. We also included compounds that did not contain favorable PK and PD properties, but whose structures were designed to further our understanding of the merulin pharmacophore. While most of the analogues elected for semi-synthesis had high ALogP values, these higher values are appropriate for drug leads against trypanosomes, as these parasites contain complex cell membranes and numerous internal vesicles, such as glycosomes, which are potentially valuable drug targets, but require compounds with high lipophilicity ranges for membrane permeability.

The selected suite of 20 carboxylic acid building blocks, primarily consisting of hetero-aromatic cores, had an array of functionalities designed to probe the pharmacophore including: heteroaromatic, basic, aliphatic and halogenated acids. These acids were each coupled to merulin A through the formation of ester linkages at position C3 using standard coupling reagents. In most cases these derivatives possessed increased computational BBB permeabilities and lower calculated hepatotoxicities than the parent scaffold. Although most of these analogues also showed increased probability of CYP2D6 inhibition, these values were in a range considered to only be moderately accurate at predicting *in vivo* CYP2D6 inhibitory activities. In all cases the QED scores for the merulin analogues were better than current HAT therapeutics, however none of the analogues had significantly better QED scores than parent **1** (figure 4).

Screening this library of merulin A analogues against the bloodstream form of *T. brucei* revealed that many of these new analogues had comparable or improved activities compared to merulin A (Figure 4). In particular, compounds **12** and **17** showed excellent antiparasitic activities (60 nM and 43 nM respectively). In order to differentiate improved selective activity against *T. brucei* rather than broad increase in cytotoxicy the merulin A library was screened against HeLa cells. HeLa cytotoxicity values were typically in the high µM range, with several of the library members having SIs>100. Possession of good BBB permeability is a requirement for treatment of the second stage HAT infections. However, with BBB penetration comes the potential for neurotoxicity and associated neurological side effects. There are currently no straightforward methods for *in vitro* evaluation of neurological side effects, however it is possible to evaluate potential neurotoxicity using *in vitro* whole cell screening. Consequently, the merulin A library was screened against glioblastoma cells as an indicator of general brain cell toxicity. IC_50_ values against glioblastoma cells were greater than 50 µM for all tested compounds, with the exception of **22** and **26**, (Figure 4) revealing a large window of efficacy and, more importantly, suggesting low cytotoxicities for these compounds against the CNS.

**Figure 5:**
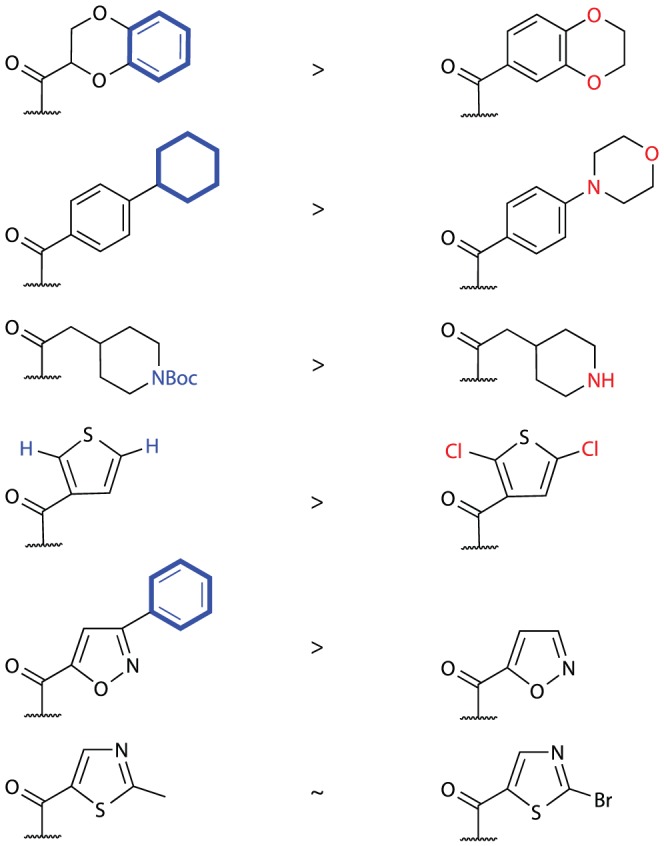
Hydrophobicity *vs.* activity comparison within analog pairs. Non-polar atoms are highlighted in blue, while the respective polar atoms are highlighted in red.

Consideration of the SAR results for this second generation library revealed a number of useful conclusions. Overall, while the ability to form hydrogen bonds was important for activity, the inclusion of additional hydrogen bond donors resulted in reduced activities (**28** and **29**). Furthermore, although inclusion of heteroaromatic sidechains lead to a general improvement in antiparasitic activities (typically a 20-fold improvement in IC_50_), inclusion of non-polar aromatic sidechains results in the most striking improvements in activity (**12** and **17**). When comparing ester analogues sharing the same core functional group, analogues with a more non-polar periphery display better *in vitro* activities than more polar analogues (Figure 5). These results suggest that, while the merulin endoperoxide warhead is a suitable motif for drug development against *T. brucei*, modifications of physicochemical properties to improve ease of transport into the parasite, are critical factors to further development efforts. One possible interpretation of this observation is that the mode of action of the merulin compounds may involve the radical mediated disruption of lipid membranes, as has been previously suggested for the mode of action of artemisinin against *Plasmodium falciparum* [22], [23].

### Site localization imaging

Little is known about the role of cyclic endoperoxides as antiparasitic agents against *T. brucei*. To explore the site localization of these compounds a fluorescent merulin derivative was designed and synthesized. Given that fused aromatic derivative **17** showed reasonable potency in the screening library, we elected to couple a 7-dimethylamino-coumarin dye to the C3 hydroxyl, forming compound **30** in an analogous fashion to the original library creation. Although coumarin dyes have only modest photophysical properties for fluorescence imaging, they are small neutral species that are ideal for use as fluorescent tags in situations such as this, where narrow cLogP ranges are required [24]. Screening of compound **30** displayed similar biological properties to the top analogues from the previous library (*T. brucei* IC_50_ = 0.42 µM, HeLa IC_50_>50 µM, SI>125).

**Figure 6:**
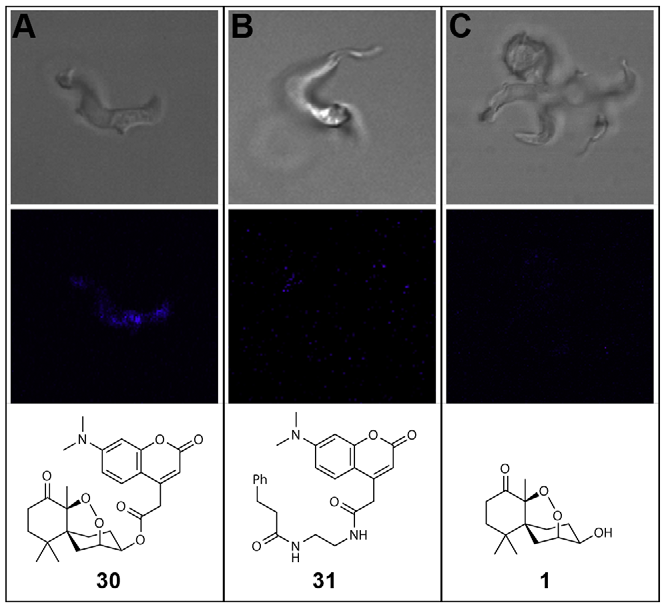
Live trypanosome fluorescence imaging. Diffuse cytoplasmic fluorescent signal of fluorophore conjugate 30, (a) indicating the uptake and accumulation of the compound into the cells. No fluorescence was detected in parasites treated with unlabeled compound **1** (b) or with free coumarin dye **31** (c).

To explore the intracellular of distribution the coumarin-labeled derivative **30**, parasites were incubated with 20 µM of **30** for 2 hours, washed once, and incubated in compound-free medium for 1 hour prior to microscopy. Fluorescence was detected in live parasites as a diffuse cytoplasmic signal, indicating the uptake and accumulation of the compound into the cells (Figure 6a). No fluorescence was detected in parasites treated with unlabeled compound **1** (Figure 6c) or with the free coumarin dye **31**, (Figure 6b) showing the specificity of the signal observed with compound **30**. Although visualization of labeled merulin A in live cell imaging is an encouraging observation, further experiments are required to determine the precise localization and potential function of these compounds against *T. brucei*.

### Conclusion

We report the semi-synthetic optimization of the merulin class of endoperoxide natural products as development candidates against *T. brucei*, one of the causative agents of African sleeping sickness. We have created a library of 25 merulin analogues using a variety of synthetic approaches, and identified several key physicochemical properties that are important for improving biological potency. Using this approach we have identified two compounds (**12** and **17**) with low nM antiparasitic activities, high selectivity indices against HeLa and glioblastoma cells, and good predicted BBB permeabilities. Together these results allow us to proceed towards meeting the TPP outlined by DND*i*'s for new drug leads against stage one and stage two HAT infections. These compounds can be produced economically in large quantities via a one step derivatization from a microbial fermentation broth isolate, making them encouraging lead candidates for further development. Finally, we have synthesized a fluorescent chemical probe based on this scaffold and demonstrated that it can be taken up by parasites in live cell imaging, which offers a potential avenue for further exploration of the mode of action of these unique antiparasitic agents.

## Materials and Methods

### Trypanosomes

*Trypanosoma brucei brucei* strain 427–221 were grown at 37°C, 5% CO_2_ in HMI-9 medium containing 10% Fetal Bovine Serum, 10% Serum Plus (JRH Inc., Lenexa, KS,USA) and penicillin/streptomycin.

### Trypanosome screening assay

T.b.b. 427–221 were diluted to 2.10^4^ per ml in complete HMI-9 medium and aliquoted in Greiner sterile 384-well flat white opaque culture plates using a WellMate cell dispenser from Matrix Tech. (Hudson, NH, USA). Test compounds were serially diluted in dimethyl sulfoxide (DMSO) and added to the assay plates with the robotic dispenser Biomek FXp liquid handler (Beckman Coulter). Thimerosal (2 µM final concentration) was added as a positive control and DMSO as a negative control (1% final concentration). Trypanosomes were incubated with compounds for 48 hours at 37°C with 5% CO_2_ before monitoring cell viability as previously described [25]. Briefly, trypanosomes were lysed in the wells by adding 25 µL of CellTiter-GloTM (Promega). The lysed trypanosomes were placed on an orbital shaker at room temperature for 2 minutes. After lysis, the resulting ATP-bioluminescence was measured at room temperature using an Analyst HT plate reader (Molecular Devices). Percentage inhibition of parasite growth was calculated for each well as [1−(RLU_x_−RLU_+_)/(RLU_−_−RLU_+_)]*100 where RLU_x_, RLU_+_ and RLU_−_ are respectively the Relative Light Units for each well, positive (thimerosal) and negative (DMSO) controls. A screening window coefficient, denoted Z′ factor, was used to evaluate the performance of the assay. The Z′ factor, calculated as 1−(3σ_c+_+3σ_c−_)/(μ_c+_−μ_c−_) where σ_c+_, σ_c−_, μ_c+_ and μ_c−_ are respectively the standard deviation and mean values of positive and negative controls, is reflective of the assay signal dynamic range and the data variation associated with signal measurement [26]. IC_50_'s were calculated using Prism (GraphPad).

### Live trypanosome fluorescence imaging

T.b.b 427–221 diluted to 5.105 mL^−1^ in complete HMI-9 medium were incubated with either 20 µM of compound **30**, 20 µM of the free coumarin dye or 30 µM of compound **17** at 37°C for 2 hours. The parasites were then washed once with complete HMI-9 medium and incubated in compound-free complete HMI-9 medium for 1 additional hour. Parasites were directly mounted under coverslips and viewed with 100×-oil immersion on a Zeiss Laser Confocal microscope LSM 510 Meta.

### HeLa toxicity screening assay

HeLa cells were plated at a density of 2,000 cells per well into two 384 well clear bottom plates (Costar 3712) in DMEM with 10% FBS. After 24 hours incubation at 37°C with 5% CO_2_ the cells were pinned with 200 nL of a two-fold serial dilution of merulin analogues. After 17 hours of treatment at 37°C in 5% CO_2_ the plates were fixed with 4% formaldehyde and washed with PBS (BioTek ELx405). After 10 minutes in PBS with 0.5% TritonX-100 each plate was washed and blocked with 2% Bovine Serum Albumin in PBS. Nuclei were stained with Hoechst 33342 then washed and placed in 40 µL of PBS with 0.1% sodium azide. Plates were imaged using the ImageXpress Micro epifluorescent scope (Molecular Devices). MetaXpress software was used to quantify total number of cells and IC_50_s were calculated using Prism (GraphPad).

### Glioblastoma toxicity screening assay

T98-G cells (ATTC number: CRL-1690) were plated at a density of 2,000 cells per well into two 384 well clear bottom plates (Costar 3712) in DMEM with 10% FBS. After 24 hours incubation at 37°C with 5% CO_2_ the cells were pinned with 200 nL of a two-fold serial dilution of merulin analogues. After 17 hours of treatment at 37°C in 5% CO_2_ the plates were fixed with 4% formaldehyde and washed with PBS (BioTek ELx405). After 10 minutes in PBS with 0.5% TritonX-100 each plate was washed and blocked with 2% Bovine Serum Albumin in PBS. Nuclei were stained with Hoechst 33342 then washed and placed in 40 µL of PBS with 0.1% sodium azide. Plates were imaged using the ImageXpress Micro epifluorescent scope (Molecular Devices). MetaXpress software was used to quantify total number of cells and IC_50_s were calculated using Prism (GraphPad).

### Computational ADMET analysis

ADMET data were acquired using Accelrys Discovery Studio Client 2.5.

### General experimental procedures

Unless otherwise stated, reactions were performed under argon atmosphere using freshly dried solvents. Tetrahydrofuran (THF) and methylene chloride (DCM) were dried by passing through activated alumina columns. All other commercially obtained reagents were used as received. Purification was performed by flash column chromatography as described by Still et al. [27] using silica gel (partical size 0.032–0.063) purchased from Silicycle or by HPLC purification using a Phenomenex Synergi C_18_ (4.6 mm×250 mm, 5 µm) RP-HPLC column unless otherwise stated. Solvents used for HPLC chromatography were HPLC grade and were used without further purification. NMR spectra and HPLC traces were obtained for all compounds (Figure S2), and purity of compounds tabulated in Table S1. Optical rotations were measured on a Jasco P-2000 polarimeter using a 10 mm path length cell at 589 nm. NMR spectra were acquired on 500 and 600 MHz spectrometers equipped with a 5 mm broadband probe and 5 mm HCN triple resonance cryoprobe, respectively, and referenced to residual solvent proton and carbon signals (δ_H_ 7.26, δ_C_ 77.16 for CDCl_3_ and δ_H_ 3.31, δ_C_ 49.00 for CD_3_OD). HRMS spectra were acquired using University of California, Berkeley QB3/Chemistry Mass Spectrometry Facility's multimode electrospray ionization (ESI) Fourier transfer mass spectrometer (FTMS).

### Experimental

#### (3S,4R,6aS,10R,10aS)-7,7,10a-trimethyloctahydro-3H-3,6a-methanobenzo[c][1,2]dioxocine-4,10-diol (8)

3.36 mg (0.0132 mmol, 1 eq) of merulin A (**1**) in 0.5 mL THF was cooled to 0°C. 1.00 mg (0.0459 mmol, 3.5 eq) of LiBH_4_ was added and the reaction stirred at 0°C for 15 minutes. TLC showed consumption of starting material **1** (TLC conditions: 5% MeOH:DCM in 0.02% HCOOH buffer; Rf of **1** = 0.23). The reaction mixture was diluted with 2 mL of EtOAc and quenched (3 mL of sat. aq. Na_2_CO_3_). The two phases were separated, and the aqueous layer extracted (3× EtOAc). The combined organics were dried (anhydrous Na_2_SO_4_), filtered, and concentrated to dryness under a stream of N_2_ gas. The crude mixture was purified with reverse phase high pressure liquid chromatography (isocratic: 56∶44 MeOH∶H_2_O with 0.02% HCOOH buffer) to give **8** as a clear oil (2.20 mg, 65%) as a single diastereomer. [α]^25^_D_ +21.8 (*c* 0.032, MeOH); UV (MeOH) *λ*max(log ε) 203 (3.03) nm; ^1^H NMR (CDCl_3_, 600 MHz) δ 4.57 (1H, ddd, *J* = 12.3, 4.9, 1.8 Hz), 4.14 (1H, td, *J* = 4.7, 1.8 Hz), 3.75 (1H, ddd, *J* = 19.6, 9.2, 4.2 Hz), 2.34 (1H, dt, *J* = 13.7, 4.4 Hz), 2.16 (3H, m), 2.08 (1H, d, *J* = 1.9 Hz), 2.00 (1H, ddd, *J* = 11.9, 8.3, 4.4 Hz), 1.88 (1H, dddd, *J* = 13.1, 4.8, 4.0, 3.1 Hz), 1.71 (1H, td, *J* = 14.4, 3.9 Hz), 1.29 (1H, dd, *J* = 13.8, 1.9 Hz), 1.27 (1H, dt, *J* = 14.2, 3.5 Hz), 1.19 (3H, s), 1.02 (3H, s), 0.85 (3H, s); ^13^C NMR (126 MHz, CDCl_3_) δ 79.31, 76.68, 70.04, 67.51, 38.19, 35.30, 32.64, 30.09, 29.52, 27.24, 26.71, 26.58, 25.14, 15.75; HRESIMS *m/z* 279.1575 [M+Na]^+^ (calcd for C_14_H_24_O_4_Na, 279.1567).

#### (1S,6S,8S,9R)-1,8,9-trihydroxy-1,5,5-trimethylspiro[5.5]undecan-2-one (9)

A suspension of **1** (2.32 mg, 0.00912 mmol) and 5% Pd/C (2.00 mg) in 2 mL of DCM were bubbled with H_2_ gas with stirring. After 2 hours, TLC showed consumption of starting material. The crude reaction mixture was filtered through a celite plug and washed with EtOAc (5 mL). The combined organics were dried (anhydrous Na_2_SO_4_), filtered, and evaporated under a stream of N_2_ gas to give **9** as a clear glass (1.10 mg, 47% yield). [α]^25^_D_ −66.0 (*c* 0.052, MeOH); UV (MeOH) *λ*max(log ε) 202 (2.64) nm; ^1^H NMR (CD_3_OD, 600 MHz) δ 3.78 (1H, q, *J* = 4.6 Hz), 3.52 (1H, dt, *J* = 7.8, 3.9 Hz), 2.78 (1H, ddd, *J* = 14.7, 12.5, 6.8 Hz), 2.36 (1H, ddd, *J* = 14.8, 5.2, 3.8 Hz), 2.03 (1H, m), 1.96 (1H, dd, *J* = 15.6, 5.3 Hz), 1.86 (3H, m), 1.62 (6H, m), 1.49 (3H, s), 1.21 (3H, s), 1.08 (3H, s); ^13^C NMR (CD_3_OD, 151 MHz) δ 215.96, 83.30, 71.56, 69.40, 40.00, 37.44, 35.39, 33.68, 29.06, 27.89, 25.50, 25.29, 24.95, 19.43; HRESIMS *m/z* 279.1569 [M+Na]^+^ (calcd for C_14_H_24_O_4_Na, 279.1567).

#### (3S,4R,6aS,10aS)-7,7,10a-trimethyl-10-oxooctahydro-3H-3,6a-methanobenzo[c][1,2]dioxocin-4-yl pivalate (11)

To a solution of **1** (3.45 mg, 0.0133 mmol, 1 eq) in DCM (1 mL) was added Boc-anhydride (23.0 mg, 0.105 mmol, 7.9 eq), and DMAP (2.0 mg, 0.0163 mmol, 1.2 eq) and the reaction mixture stirred at room temperature until TLC showed consumption of starting material **1** (6 hours). The crude reaction mixture was concentrated to dryness under a stream of N_2_ gas, and purified by column chromatography through a silica column (1% MeOH∶DCM with 0.02% HCOOH buffer) to give **11** (4.70 mg, 99% yield) as a white solid. [α]^25^_D_ +49.7 (*c* 0.044, MeOH); UV (MeOH) *λ*max(log ε) 202 (2.96) nm; ^1^H NMR (CDCl_3_, 600 MHz) δ 4.67 (1H, ddd, *J* = 11.3, 7.6, 3.8 Hz), 4.39 (1H, td, *J* = 4.2, 2.0 Hz), 2.67 (1H, ddd, *J* = 15.4, 14.4, 6.6 Hz), 2.49 (1H, dddd, *J* = 13.9, 12.8, 11.3, 6.3 Hz), 2.42 (1H, ddd, *J* = 15.4, 4.8, 2.4 Hz), 2.16 (1H, dddd, *J* = 14.3, 6.0, 4.1, 1.6 Hz), 2.10 (1H, dddd, *J* = 14.5, 8.0, 4.3, 1.5 Hz), 2.00 (2H, m), 1.62 (1H, tdd, *J* = 14.2, 6.2 Hz), 1.57 (2H, m), 1.46 (9H, s), 1.40 (3H, s), 1.25 (3H, s), 0.97 (3H, s); ^13^C NMR (CDCl_3_, 151 MHz) δ 208.09, 153.30, 90.00, 82.46, 75.66, 74.45, 41.41, 37.45, 35.92, 35.76, 30.46, 27.93, 27.47, 26.31, 25.43, 24.79, 21.53. HRESIMS *m/z* 337.1951 [M+Na]^+^ (calcd for C_19_H_30_O_6_Na, 337.1934).

### General synthesis of esters

Merulin A (**1**, 1 eq), caboxylic acid (1.2 eq), EDCI (2 eq), and DMAP (0.1 eq) were stirred under argon in DCM until TLC showed consumption of starting material **1** (Usually 2–8 hours; TLC condition: 5% MeOH∶DCM in 0.02% HCOOH buffer; Rf of **1** = 0.23). Crude reaction mixtures were dried under a stream of N_2_ gas and purified with silica chromatography (1% MeOH∶DCM in 0.02% HCOOH buffer gradient to 5% MeOH∶DCM in 0.02% HCOOH buffer). Purity was determined by a combination of LCMS and ^1^H NMR analysis (All products >95% pure).

#### (3S,4R,6aS,10aS)-7,7,10a-trimethyl-10-oxooctahydro-3H-3,6a-methanobenzo[c][1,2]dioxocin-4-yl acetate (10)

[α]^25^_D_ +256.25 (*c* 0.037, MeOH); UV (MeOH) *λ*max(log ε) 202 (3.09) nm; ^1^H NMR (CDCl_3_, 600 MHz) δ 4.90 (1H, ddd, *J* = 11.3, 7.6, 3.8 Hz), 4.26 (1H, td, *J* = 4.3, 2.0 Hz), 2.68 (1H, ddd, *J* = 15.4, 14.5, 6.7 Hz), 2.44 (2H, m), 2.16 (1H, dddd, *J* = 14.3, 5.9, 3.6, 1.5 Hz), 2.10 (3H, s), 2.06 (1H, dd, *J* = 12.8, 6.5 Hz), 2.01 (2H, dt, *J* = 14.9, 4.8 Hz), 1.63 (1H, td, *J* = 14.1, 6.2 Hz), 1.60 (1H, dd, *J* = 13.6, 2.0 Hz), 1.57 (1H, ddd, *J* = 14.3, 6.7, 2.5 Hz), 1.41 (3H, s), 1.26 (3H, s), 0.98 (3H, s); ^13^C NMR (CDCl_3_, 151 MHz) δ 207.89, 171.09, 90.08, 76.06, 71.74, 41.06, 37.47, 35.88, 35.75, 30.60, 27.52, 26.31, 25.44, 24.78, 21.56, 21.35; HRESIMS *m/z* 319.1520 [M+Na]^+^ (calcd for C_16_H_24_O_5_Na, 319.1516).

#### (3S,4R,6aS,10aS)-7,7,10a-trimethyl-10-oxooctahydro-3H-3,6a-methanobenzo[c][1,2]dioxocin-4-yl 2,3-dihydrobenzo[b][1,4]dioxine-2-carboxylate (12)

[α]^25^_D_ +109.6 (*c* 0.052, MeOH); UV (MeOH) *λ*max(log ε) 202 (4.00), 276 (2.96), 282 (2.93) nm; ^1^H NMR (CDCl_3_, 600 MHz) δ 8.79 (s, 1H), 8.29 (dd, *J* = 8.0, 1.7 Hz, 1H), 7.70 (ddd, *J* = 8.6, 7.2, 1.7 Hz, 1H), 7.50 (dt, *J* = 8.4, 2.1 Hz, 1H), 7.45 (ddd, *J* = 8.1, 7.2, 1.0 Hz, 1H), 5.18 (ddd, *J* = 11.3, 7.6, 3.9 Hz, 1H), 4.35 (td, *J* = 4.3, 1.9 Hz, 1H), 2.70 (td, *J* = 14.8, 6.7 Hz, 1H), 2.53 (tdd, *J* = 12.6, 10.8, 6.2 Hz, 1H), 2.46 (ddd, *J* = 15.4, 4.7, 2.4 Hz, 1H), 2.24–2.18 (m, 2H), 2.07 (dt, *J* = 13.7, 4.2 Hz, 1H), 2.02 (td, *J* = 14.4, 4.7 Hz, 1H), 1.70 (td, *J* = 14.2, 6.2 Hz, 1H), 1.67 (dd, *J* = 13.8, 1.8 Hz, 1H), 1.60 (ddd, *J* = 14.3, 6.7, 2.4 Hz, 1H), 1.45 (s, 3H), 1.28 (s, 3H), 1.01 (s, 3H); ^13^C NMR (CDCl_3_, 151 MHz) δ 207.76, 142.44, 122.34, 121.92, 117.58, 117.49, 117.45, 117.37, 75.82, 73.29, 73.04, 72.00, 65.08, 41.42, 37.49, 35.72, 30.55, 30.43, 27.48, 26.32, 25.36, 24.79, 21.60; HRESIMS *m/z* 439.1732 [M+Na]^+^ (calcd for C_23_H_28_O_7_Na_1_, 439.1727).

#### (3S,4R,6aS,10aS)-7,7,10a-trimethyl-10-oxooctahydro-3H-3,6a-methanobenzo[c][1,2]dioxocin-4-yl 2,3-dihydrobenzo[b][1,4]dioxine-6-carboxylate (13)

[α]^25^_D_ +92.3 (*c* 0.052, MeOH); UV (MeOH) *λ*max(log ε) 211 (3.97), 220 (3.48), 261 (3.48), 295 (3.48) nm; ^1^H NMR (CDCl_3_, 600 MHz) δ 7.64 (1H, d, *J* = 1.9 Hz), 7.62 (1H, dd, *J* = 8.4, 2.0 Hz), 6.86 (1H, d, *J* = 8.4 Hz), 5.12 (1H, ddd, *J* = 11.2, 7.5, 3.8 Hz), 4.32 (1H, td, *J* = 4.2, 1.9 Hz), 4.30 (2H, dd, *J* = 5.3, 2.7 Hz), 4.26 (2H, dd, *J* = 5.3, 2.5 Hz), 2.69 (1H, td, *J* = 14.9, 6.7 Hz), 2.55 (1H, dtd, *J* = 13.0, 11.5, 6.4 Hz), 2.45 (1H, ddd, *J* = 15.4, 4.6, 2.3 Hz), 2.19 (2H, m), 2.05 (1H, dt, *J* = 13.4, 4.1 Hz), 2.02 (1H, td, *J* = 14.4, 4.7 Hz), 1.69 (1H, td, *J* = 14.0, 6.2 Hz), 1.66 (1H, dd, *J* = 13.5, 1.7 Hz), 1.59 (1H, ddd, *J* = 14.3, 6.6, 2.2 Hz), 1.44 (3H, s), 1.28 (3H, s), 1.01 (3H, s); ^13^C NMR (CDCl_3_, 151 MHz) δ 207.94 165.84, 148.02, 143.27, 123.91, 123.54, 119.48, 117.15, 90.10, 77.16, 76.53, 71.83, 64.80, 64.19, 41.55, 37.50, 35.88, 35.77, 30.78, 27.74, 26.37, 25.51, 24.82, 21.65; HRESIMS *m/z* 439.1732 [M+Na]^+^ (calcd for C_23_H_28_O_7_Na_1_, 439.1727).

#### (3S,4R,6aS,10aS)-7,7,10a-trimethyl-10-oxooctahydro-3H-3,6a-methanobenzo[c][1,2]dioxocin-4-yl 2-oxo-2H-chromene-3-carboxylate (14)

[α]^25^_D_ +81.1 (*c* 0.053, MeOH); UV (MeOH) *λ*max(log ε) 203 (3.98), 293 (3.66), 331 (3.38) nm; ^1^H NMR (CDCl_3_, 600 MHz) δ 8.63 (1H, s), 7.64 (2H, m), 7.34 (2H, m), 5.18 (1H, ddd, *J* = 11.2, 7.6, 3.9 Hz), 4.35 (1H, s), 2.70 (1H, td, *J* = 14.9, 6.7 Hz), 2.55 (1H, ddd, *J* = 25.0, 13.2, 6.5 Hz), 2.46 (1H, ddd, *J* = 15.3, 4.4, 2.1 Hz), 2.22 (2H, m), 2.08 (1H, dt, *J* = 13.8, 4.1 Hz), 2.02 (1H, td, *J* = 14.4, 4.7 Hz), 1.71 (1H, td, *J* = 14.3, 6.3 Hz), 1.68 (1H, dd, *J* = 13.4, 1.2 Hz), 1.60 (1H, ddd, *J* = 14.2, 6.6, 2.0 Hz), 1.45 (3H, s), 1.28 (3H, s), 1.01 (3H, s); ^13^C NMR (CDCl_3_, 151 MHz) δ 207.92, 161.51, 156.89, 155.26, 149.04, 134.60, 129.87, 125.06, 117.99, 117.59, 116.91, 90.22, 76.25, 72.48, 41.49, 37.50, 35.82, 35.72, 30.64, 27.61, 26.35, 25.44, 24.79, 21.66; HRESIMS *m/z* 449.1576 [M+Na]^+^ (calcd for C_24_H_26_O_7_Na_1_, 449.1571).

#### (3S,4R,6aS,10aS)-7,7,10a-trimethyl-10-oxooctahydro-3H-3,6a-methanobenzo[c][1,2]dioxocin-4-yl [1,1′-biphenyl]-3-carboxylate (15)

[α]^25^_D_ +131.4 (*c* 0.054, MeOH); UV (MeOH) *λ*max(log ε) 202 (4.25), 231 (4.10) nm; ^1^H NMR (CDCl_3_, 500 MHz) δ 8.31 (1H, dd, *J* = 2.5, 1.0 Hz), 8.09 (1H, dt, *J* = 7.7, 1.4 Hz), 7.77 (1H, ddd, *J* = 7.7, 1.8, 1.2 Hz), 7.62 (1H, t, *J* = 1.6 Hz), 7.60 (1H, d, *J* = 0.7 Hz), 7.49 (1H, t, *J* = 7.7 Hz), 7.45 (2H, t, *J* = 7.6 Hz), 7.37 (1H, t, *J* = 7.4 Hz), 5.19 (1H, ddd, *J* = 11.2, 7.4, 3.8 Hz), 4.37 (1H, td, *J* = 4.5, 1.9 Hz), 3.70 (1H, ddd, *J* = 17.0, 11.3, 4.7 Hz), 2.70 (1H, td, *J* = 14.8, 6.7 Hz), 2.45 (1H, ddd, *J* = 15.4, 4.7, 2.3 Hz), 2.22 (2H, m), 2.08 (1H, dt, *J* = 13.6, 4.2 Hz), 2.02 (1H, td, *J* = 14.4, 4.8 Hz), 1.71 (1H, td, *J* = 14.4, 6.4 Hz), 1.69 (1H, dd, *J* = 13.7, 1.8 Hz), 1.59 (1H, ddd, *J* = 14.2, 6.6, 2.3 Hz), 1.45 (3H, s), 1.28 (3H, s), 1.01 (3H, s); ^13^C NMR (CDCl_3_, 126 MHz) δ 208.17, 166.33, 141.59, 140.24, 131.84, 130.82, 129.00, 128.91, 128.86, 128.54, 127.82, 127.36, 76.51, 72.30, 64.38, 41.13, 37.50, 35.88, 35.76, 30.77, 27.70, 26.36, 25.48, 24.82, 21.64; HRESIMS *m/z* 457.1979 [M+Na]^+^ (calcd for C_27_H_30_O_5_Na_1_, 457.1985).

#### (3S,4R,6aS,10aS)-7,7,10a-trimethyl-10-oxooctahydro-3H-3,6a-methanobenzo[c][1,2]dioxocin-4-yl 4-cyclohexylbenzoate (16)

[α]^25^_D_ +129.0 (*c* 0.055, MeOH); UV (MeOH) *λ*max(log ε) 203 (4.17), 243 (3.92), 297 (2.96) nm; ^1^H NMR (CDCl_3_, 600 MHz) δ 8.02 (2H, d, *J* = 8.3 Hz), 7.24 (2H, d, *J* = 8.2 Hz), 5.15 (1H, ddd, *J* = 11.3, 7.5, 3.8 Hz), 4.33 (1H, td, *J* = 4.3, 2.0 Hz), 4.28 (1H, m), 2.70 (1H, ddd, *J* = 15.3, 14.4, 6.7 Hz), 2.56 (2H, dddd, *J* = 17.5, 11.2, 8.8, 4.5 Hz), 2.45 (1H, ddd, *J* = 15.4, 4.7, 2.3 Hz), 2.19 (2H, m), 2.07 (1H, dt, *J* = 13.4, 4.3 Hz), 2.02 (1H, td, *J* = 14.5, 4.6 Hz), 1.85 (4H, m), 1.75 (1H, m), 1.70 (1H, td, *J* = 14.2, 6.2 Hz), 1.68 (1H, dd, *J* = 13.7, 2.0 Hz), 1.60 (1H, dt, *J* = 4.4, 2.4 Hz), 1.45 (3H, s), 1.40 (4H, m), 1.28 (3H, s), 1.01 (3H, s). ^13^C NMR (CDCl_3_, 151 MHz) δ 208.05 166.42, 130.16, 127.86, 126.93, 117.15, 90.11, 76.60, 71.78, 44.86, 41.56, 37.50, 35.88, 35.77, 34.29, 34.26, 31.09, 30.80, 27.75, 26.89, 26.37, 26.19, 25.51, 24.82, 21.66; HRESIMS *m/z* 463.2450 [M+Na]^+^ (calcd for C_27_H_36_O_5_Na_1_, 463.2455).

#### (3S,4R,6aS,10aS)-7,7,10a-trimethyl-10-oxooctahydro-3H-3,6a-methanobenzo[c][1,2]dioxocin-4-yl 2-(naphthalen-1-yl)acetate (17)

[α]^25^_D_ +126.9 (*c* 0.052, MeOH); UV (MeOH) *λ*max(log ε) 228 (4.52), 281 (3.50), 272 (3.42) nm; ^1^H NMR (CDCl_3_, 600 MHz) δ 8.01 (1H, dd, *J* = 8.2, 0.8 Hz), 7.85 (1H, m), 7.78 (1H, m), 7.53 (1H, ddd, *J* = 8.4, 6.8, 1.4 Hz), 7.48 (1H, ddd, *J* = 8.0, 6.8, 1.2 Hz), 7.43 (1H, s), 7.42 (1H, d, *J* = 2.5 Hz), 4.92 (1H, ddd, *J* = 11.3, 7.6, 3.8 Hz), 4.27 (1H, td, *J* = 4.3, 2.0 Hz), 4.13 (2H, d, *J* = 4.8 Hz), 2.69 (1H, ddd, *J* = 15.4, 14.4, 6.7 Hz), 2.49 (1H, dddd, *J* = 13.2, 12.4, 11.1, 6.0 Hz), 2.44 (1H, ddd, *J* = 15.5, 4.8, 2.5 Hz), 2.15 (1H, dddd, *J* = 14.0, 5.5, 3.7, 1.3 Hz), 2.01 (3H, m), 1.61 (1H, td, *J* = 14.1, 6.3 Hz), 1.58 (2H, m), 1.44 (3H, s), 1.25 (3H, s), 0.96 (3H, s); ^13^C NMR (CDCl_3_, 151 MHz) δ 208.05, 171.59, 133.94, 132.25, 130.62, 128.79, 128.28, 128.21, 126.54, 125.88, 125.65, 124.04, 94.57, 76.12, 72.29, 41.47, 39.19, 37.48, 35.87, 35.77, 30.59, 27.50, 26.32, 25.42, 24.79, 21.61; HRESIMS *m/z* 445.1990 [M+Na]^+^ (calcd for C_26_H_30_O_5_Na_1_, 445.1985).

#### (3S,4R,6aS,10aS)-7,7,10a-trimethyl-10-oxooctahydro-3H-3,6a-methanobenzo[c][1,2]dioxocin-4-yl 2-methylthiazole-5-carboxylate (18)

[α]^25^_D_ +75.9 (*c* 0.047, MeOH); UV (MeOH) *λ*max(log ε) 201 (3.89), 255 (3.70) nm; ^1^H NMR (CDCl_3_, 600 MHz) δ 8.28 (1H, s), 5.10 (1H, ddd, *J* = 11.1, 7.5, 3.8 Hz), 4.33 (1H, td, *J* = 4.5, 1.9 Hz), 2.73 (3H, s), 2.69 (1H, td, *J* = 14.9, 6.7 Hz), 2.56 (1H, m), 2.44 (1H, ddd, *J* = 15.4, 4.7, 2.4 Hz), 2.19 (2H, m), 2.07 (1H, td, *J* = 4.2, 4.2, 13.7 Hz), 2.00 (1H, dt, *J* = 14.4, 7.2 Hz), 1.68 (1H, dt, *J* = 14.5, 5.7 Hz), 1.65 (1H, dd, *J* = 13.7, 1.8 Hz), 1.59 (1H, ddd, *J* = 14.3, 6.7, 2.4 Hz), 1.44 (3H, s), 1.27 (3H, s), 1.00 (3H, s); ^13^C NMR (CDCl_3_, 151 MHz) δ 215.72, 172.58, 161.14, 148.84, 129.00, 90.11, 76.18, 72.82, 41.47, 37.48, 35.85, 35.72, 30.66, 27.65, 26.34, 25.44, 24.80, 21.62, 19.88; HRESIMS *m/z* 380.1530 [M+H]^+^ (calcd for C_19_H_26_ N_1_O_5_ S_1_, 380.1530).

#### (3S,4R,6aS,10aS)-7,7,10a-trimethyl-10-oxooctahydro-3H-3,6a-methanobenzo[c][1,2]dioxocin-4-yl 2-bromothiazole-5-carboxylate (19)

[α]^25^_D_ +107.2 (*c* 0.055, MeOH); UV (MeOH) *λ*max(log ε) 264 (3.58), 203 (3.34) nm; ^1^H NMR (CDCl_3_, 600 MHz) δ 8.19 (1H, s), 5.11 (1H, ddd, *J* = 11.2, 7.6, 3.8 Hz), 4.33 (1H, s), 2.69 (1H, td, *J* = 14.9, 6.7 Hz), 2.55 (1H, ddd, *J* = 24.4, 13.0, 6.5 Hz), 2.44 (1H, ddd, *J* = 15.4, 4.6, 2.3 Hz), 2.19 (2H, m), 2.06 (1H, dt, *J* = 13.8, 4.2 Hz), 2.01 (1H, td, *J* = 14.4, 4.7 Hz), 1.69 (1H, td, *J* = 14.1, 6.2 Hz), 1.65 (1H, dd, *J* = 13.7, 1.7 Hz), 1.58 (1H, ddd, *J* = 14.3, 6.7, 2.3 Hz), 1.44 (3H, s), 1.27 (3H, s), 1.00 (3H, s); ^13^C NMR (CDCl_3_, 151 MHz) δ 191.65, 159.79, 148.43, 142.55, 133.03, 90.14, 76.00, 73.39, 41.44, 37.48, 35.83, 35.70, 30.60, 27.61, 26.33, 25.41, 24.79, 21.61; HRESIMS *m/z* 544.0438 [M+DMSO+Na]^+^ (calcd for C_20_H_26_Br_1_ N_1_O_6_ S_2_Na_1_, 544.0421).

#### (3S,4R,6aS,10aS)-7,7,10a-trimethyl-10-oxooctahydro-3H-3,6a-methanobenzo[c][1,2]dioxocin-4-yl thiophene-3-carboxylate (20)

[α]^25^_D_ +189.01 (*c* 0.046, MeOH); UV (MeOH) *λ*max(log ε) 201 (3.82), 241 (3.49) nm; ^1^H NMR (CDCl_3_, 500 MHz) δ 8.18 (1H, dd, *J* = 3.1, 1.2 Hz), 7.56 (1H, dd, *J* = 5.1, 1.2 Hz), 7.28 (1H, dd, *J* = 5.1, 3.1 Hz), 5.11 (1H, ddd, *J* = 11.2, 7.4, 3.8 Hz), 4.33 (1H, td, *J* = 4.3, 2.0 Hz), 2.69 (1H, ddd, *J* = 15.2, 14.6, 6.7 Hz), 2.55 (1H, ddd, *J* = 13.7, 11.2, 6.8 Hz), 2.44 (1H, ddd, *J* = 15.4, 4.8, 2.4 Hz), 2.19 (2H, m), 2.06 (1H, dt, *J* = 13.6, 4.4 Hz), 2.01 (1H, td, *J* = 14.4, 4.8 Hz), 1.70 (1H, td, *J* = 14.2, 6.2 Hz), 1.67 (1H, dd, *J* = 13.7, 2.0 Hz), 1.59 (1H, ddd, *J* = 14.3, 6.7, 2.4 Hz), 1.44 (3H, s), 1.27 (3H, s), 1.00 (3H, s); ^13^C NMR (CDCl_3_, 126 MHz) δ 208.10, 162.46, 133.40, 128.19, 126.01, 90.11, 76.48, 71.83, 41.54, 41.10, 35.87, 35.75, 30.76, 27.73, 26.36, 25.48, 24.81, 21.64; HRESIMS *m/z* 365.1569 [M+H]^+^ (calcd for C_19_H_25_O_2_S_1_, 365.1570).

#### (3S,4R,6aS,10aS)-7,7,10a-trimethyl-10-oxooctahydro-3H-3,6a-methanobenzo[c][1,2]dioxocin-4-yl 2,5-dichlorothiophene-3-carboxylate (21)

[α]^25^_D_ +151.8 (*c* 0.054, MeOH); UV (MeOH) *λ*max(log ε) 214 (3.99), 244 (3.47) nm; ^1^H NMR (CDCl_3_, 600 MHz) δ 7.28 (1H, s), 5.09 (1H, ddd, *J* = 11.2, 7.5, 3.9 Hz), 4.31 (1H, br s), 2.69 (1H, td, *J* = 14.9, 6.7 Hz), 2.53 (1H, dtd, *J* = 13.2, 11.4, 6.5 Hz), 2.44 (1H, ddd, *J* = 15.4, 4.7, 2.4 Hz), 2.19 (2H, m), 2.06 (1H, d, *J* = 13.7 Hz), 2.01 (1H, td, *J* = 14.4, 4.7 Hz), 1.68 (1H, td, *J* = 14.0, 6.3 Hz), 1.65 (1H, dd, *J* = 13.8, 1.9 Hz), 1.59 (1H, ddd, *J* = 14.4, 6.7, 2.4 Hz), 1.43 (3H, s), 1.27 (1H, s), 1.00 (1H, s); ^13^C NMR (CDCl_3_, 151 MHz) δ 207.76, 160.32, 135.10, 128.27, 127.67, 126.30, 90.13, 76.21, 72.45, 41.49, 37.48, 35.84, 35.72, 30.64, 27.66, 26.34, 25.43, 24.79, 21.62; HRESIMS *m/z* 533.0599 [M+DMSO+Na]^+^ (calcd for C_19_H_25_O_2_S_1_, 365.1570).

#### (3S,4R,6aS,10aS)-7,7,10a-trimethyl-10-oxooctahydro-3H-3,6a-methanobenzo[c][1,2]dioxocin-4-yl 3-phenylisoxazole-5-carboxylate (22)

[α]^25^_D_ +137.5 (*c* 0.053, MeOH); UV (MeOH) *λ*max(log ε) 200 (4.14), 228 (4.00) nm; ^1^H NMR (CDCl_3_, 600 MHz) δ 7.83 (2H, m), 7.48 (3H, m), 7.31 (1H, s), 5.21 (1H, ddd, *J* = 11.2, 7.6, 3.9 Hz), 4.39 (1H, td, *J* = 4.4, 1.9 Hz), 2.71 (1H, td, *J* = 14.8, 6.7 Hz), 2.62 (1H, tdd, *J* = 13.3, 11.4, 6.6 Hz), 2.46 (1H, ddd, *J* = 15.4, 4.7, 2.4 Hz), 2.24 (2H, m), 2.10 (1H, dt, *J* = 13.8, 4.3 Hz), 2.03 (1H, td, *J* = 14.4, 4.7 Hz), 1.72 (1H, td, *J* = 14.3, 6.3 Hz), 1.68 (1H, dd, *J* = 13.9, 2.0 Hz), 1.61 (1H, ddd, *J* = 14.3, 6.7, 2.4 Hz), 1.46 (3H, s), 1.29 (3H, s), 1.02 (3H, s); ^13^C NMR (CDCl_3_, 151 MHz) δ 207.52, 163.15, 156.52, 153.86, 130.68, 129.24, 128.12, 127.01, 109.90, 107.87, 80.46, 75.96, 73.59, 41.48, 37.51, 35.72, 30.64, 27.60, 26.34, 25.45, 24.81, 21.64; HRESIMS *m/z* 448.1734 [M+Na]^+^ (calcd for C_24_H_27_ N_1_O_6_Na_1_, 448.1731).

#### (3S,4R,6aS,10aS)-7,7,10a-trimethyl-10-oxooctahydro-3H-3,6a-methanobenzo[c][1,2]dioxocin-4-yl isoxazole-5-carboxylate (23)

[α]^25^_D_ +144.1 (*c* 0.044, MeOH); UV (MeOH) *λ*max(log ε) 225 (3.48) nm; ^1^H NMR (CDCl_3_, 600 MHz) δ 8.36 (1H, d, *J* = 1.9 Hz), 6.98 (1H, d, *J* = 1.9 Hz), 5.17 (1H, ddd, *J* = 11.3, 7.6, 4.1 Hz), 4.37 (0H, m), 2.69 (1H, td, *J* = 14.8, 6.7 Hz), 2.60 (1H, ddt, *J* = 19.2, 13.0, 6.3 Hz), 2.44 (1H, ddd, *J* = 15.4, 4.5, 2.3 Hz), 2.22 (2H, m), 2.07 (2H, dt, *J* = 13.7, 4.1 Hz), 2.01 (1H, td, *J* = 14.5, 4.7 Hz), 1.70 (1H, td, *J* = 14.0, 6.4 Hz), 1.66 (1H, dd, *J* = 13.7, 1.5 Hz), 1.60 (1H, ddd, *J* = 14.3, 6.7, 2.0 Hz), 1.44 (3H, s), 1.27 (3H, s), 1.00 (3H, s); ^13^C NMR (CDCl_3_, 126 MHz) δ 207.69, 159.67, 156.34, 150.76, 109.07, 90.13, 75.82, 73.71, 41.40, 37.45, 35.80, 35.67, 30.55, 27.50, 26.28, 25.37, 24.75, 21.57; HRESIMS *m/z* 372.1421 [M+Na]^+^ (calcd for C_18_H_23_N_1_O_6_Na_1_, 372.1423).

#### (3S,4R,6aS,10aS)-7,7,10a-trimethyl-10-oxooctahydro-3H-3,6a-methanobenzo[c][1,2]dioxocin-4-yl 4-morpholinobenzoate (24)

[α]^25^_D_ +103.6 (*c* 0.055, MeOH); UV (MeOH) *λ*max(log ε) 302 (4.04), 201 (4.04), 223 (3.56) nm; ^1^H NMR (CDCl_3_, 600 MHz) δ 8.00 (2H, m), 6.83 (2H, m), 5.13 (1H, ddd, *J* = 11.2, 7.5, 3.8 Hz), 4.32 (1H, td, *J* = 4.5, 1.9 Hz), 3.85 (4H, dd, *J* = 5.5, 4.3 Hz), 3.27 (4H, m), 2.69 (1H, td, *J* = 14.8, 6.7 Hz), 2.55 (1H, tdd, *J* = 13.0, 11.1, 6.1 Hz), 2.44 (1H, ddd, *J* = 15.4, 4.7, 2.4 Hz), 2.19 (2H, m), 2.05 (1H, dt, *J* = 13.5, 4.2 Hz), 2.02 (1H, td, *J* = 14.3, 4.7 Hz), 1.70 (1H, td, *J* = 14.2, 6.2 Hz), 1.67 (1H, ddd, *J* = 13.6, 1.9 Hz), 1.58 (1H, ddd, *J* = 14.3, 6.7, 2.4 Hz), 1.44 (3H, s), 1.27 (3H, S), 1.01 (3H, s); ^13^C NMR (CDCl_3_, 151 MHz) δ 208.00, 154.46, 132.65, 131.70, 120.39, 113.51, 90.09, 76.72, 71.45, 66.75, 47.85, 41.56, 37.48, 35.87, 35.76, 30.81, 27.80, 26.36, 25.51, 24.82, 21.64; HRESIMS *m/z* 544.2345 [M+DMSO+Na]^+^ (calcd for C_27_H_39_ N_1_O_6_S_1_Na_1_, 544.2339).

#### (3S,4R,6aS,10aS)-7,7,10a-trimethyl-10-oxooctahydro-3H-3,6a-methanobenzo[c][1,2]dioxocin-4-yl isonicotinate (25)

[α]^25^_D_ +165.9 (*c* 0.045, MeOH); UV (MeOH) *λ*max(log ε) 211 (3.55), 274 (3.16) nm; ^1^H NMR (CDCl_3_, 600 MHz) δ 8.76 (1H, d, *J* = 1.6 Hz), 8.76 (1H, d, *J* = 1.6 Hz), 7.91 (1H, d, *J* = 1.6 Hz), 7.90 (1H, d, *J* = 1.6 Hz), 5.18 (1H, ddd, *J* = 11.1, 7.5, 3.9 Hz), 4.36 (1H, td, *J* = 4.4, 1.9 Hz), 2.70 (1H, ddd, *J* = 15.3, 14.4, 6.7 Hz), 2.59 (1H, tdd, *J* = 13.1, 11.0, 6.2 Hz), 2.45 (1H, ddd, *J* = 15.4, 4.8, 2.4 Hz), 2.22 (2H, m), 2.09 (1H, ddd, *J* = 13.7, 4.6, 3.5 Hz), 2.02 (1H, td, *J* = 14.3, 4.4 Hz), 1.71 (1H, td, *J* = 14.3, 6.1 Hz), 1.68 (1H, dd, *J* = 13.8, 2.1 Hz), 1.60 (1H, ddd, *J* = 14.4, 6.7, 2.4 Hz), 1.45 (3H, s), 1.28 (3H, s), 1.01 (3H, s); ^13^C NMR (CDCl_3_, 126 MHz) δ 207.82, 164.77, 150.38, 137.78, 123.34, 90.18, 76.13, 73.07, 41.50, 37.51, 35.86, 35.72, 30.66, 27.63, 26.34, 25.45, 24.80, 21.63; HRESIMS *m/z* 360.1810 [M+H]^+^ (calcd for C_20_H_26_N_1_O_5_Na_1_, 360.1805).

#### (3S,4R,6aS,10aS)-7,7,10a-trimethyl-10-oxooctahydro-3H-3,6a-methanobenzo[c][1,2]dioxocin-4-yl 6-(piperidin-1-yl)nicotinate (26)

[α]^25^_D_ +74.5 (*c* 0.055, MeOH); UV (MeOH) *λ*max(log ε) 302 (3.97), 201 (3.48) nm; ^1^H NMR (CDCl_3_, 600 MHz) δ 8.83 (1H, d, *J* = 2.3 Hz), 8.02 (1H, dd, *J* = 9.1, 2.4 Hz), 6.52 (1H, d, *J* = 9.1 Hz), 5.11 (1H, ddd, *J* = 11.2, 7.5, 3.8 Hz), 4.32 (1H, td, *J* = 4.5, 1.9 Hz), 3.27 (4H, m), 2.69 (1H, td, *J* = 14.8, 6.7 Hz), 2.53 (1H, tdd, *J* = 13.0, 11.5, 6.4 Hz), 2.44 (1H, ddd, *J* = 15.4, 4.7, 2.4 Hz), 2.17 (2H, m), 2.05 (1H, dt, *J* = 13.6, 4.2 Hz), 2.00 (1H, dd, *J* = 14.4, 4.7 Hz), 1.69 (1H, td, *J* = 13.8, 6.1 Hz), 1.66 (1H, dd, *J* = 13.7, 1.9 Hz), 1.62 (6H, m), 1.58 (1H, td, *J* = 6.6, 2.4 Hz), 1.43 (3H, s), 1.27 (3H, s), 1.00 (3H, s); ^13^C NMR (CDCl_3_, 151 MHz) δ 207.99, 165.83, 160.84, 151.84, 138.64, 113.71, 104.87, 90.07, 76.64, 71.36, 46.01, 41.55, 37.48, 35.87, 35.76, 30.79, 27.80, 26.36, 25.69, 25.50, 24.83, 21.64, 21.63; HRESIMS *m/z* 443.2545 [M+H]^+^ (calcd for C_25_H_35_N_2_O_5_, 443.2540).

#### tert-butyl-4-(2-oxo-2-(((3S,4R,6aS,10aS)-7,7,10a-trimethyl-10-oxooctahydro-3H-3,6a-methanobenzo[c][1,2]dioxocin-4-yl)oxy)ethyl)piperidine-1-carboxylate (27)

[α]^25^_D_ +81.9 (*c* 0.062, MeOH); UV (MeOH) *λ*max(log ε) 202 (3.37) nm; ^1^H NMR (CDCl_3_, 600 MHz) δ 4.91 (1H, ddd, *J* = 11.3, 7.6, 3.8 Hz), 4.24 (1H, td, *J* = 4.5, 2.1 Hz), 4.05 (2H, dq, *J* = 13.5, 2.4 Hz), 2.69 (3H, m), 2.46 (1H, m), 2.43 (1H, ddd, *J* = 15.2, 4.6, 2.3 Hz), 2.28 (2H, h, *J* = 7.4 Hz), 2.16 (1H, dt, *J* = 14.4, 4.3 Hz), 2.01 (3H, m), 1.94 (1H, ddd, *J* = 11.4, 7.6, 3.9 Hz), 1.65 (3H, m), 1.60 (1H, dd, *J* = 13.5, 1.9 Hz), 1.56 (1H, td, *J* = 7.7, 2.4 Hz), 1.44 (9H, s), 1.41 (3H, s), 1.25 (3H, s), 1.15 (2H, qt, *J* = 12.5, 4.1 Hz), 0.98 (3H, s); ^13^C NMR (CDCl_3_, 151 MHz) δ 207.98, 172.38, 154.93, 90.10, 79.46, 76.10, 71.71, 43.81, 41.46, 41.41, 37.48, 35.89, 35.75, 33.28, 31.93, 31.90, 30.57, 28.60, 27.56, 26.32, 25.43, 24.78, 21.56; HRESIMS *m/z* 502.2780 [M+Na]^+^ (calcd for C_26_H_41_N_1_O_7_Na_1_, 502.2775).

#### (3S,4R,6aS,10aS)-7,7,10a-trimethyl-10-oxooctahydro-3H-3,6a-methanobenzo[c][1,2]dioxocin-4-yl 2-(piperidin-4-yl)acetate (28)

[α]^25^_D_ +181.1 (*c* 0.088, MeOH); UV (MeOH) *λ*max(log ε) 202 (3.28), 262 (2.64) nm; ^1^H NMR (CD_3_OD, 600 MHz) δ 4.96 (1H, ddd, *J* = 11.3, 7.7, 3.8 Hz), 4.25 (1H, td, *J* = 4.3, 2.0 Hz), 3.38 (2H, m), 3.01 (2H, tdd, *J* = 12.8, 5.0, 2.9 Hz), 2.85 (1H, ddd, *J* = 15.6, 14.4, 6.8 Hz), 2.37 (4H, m), 2.22 (1H, dddd, *J* = 14.9, 6.1, 3.7, 1.6 Hz), 2.11 (1H, dddd, *J* = 18.6, 7.9, 6.9, 3.4 Hz), 2.01 (5H, m), 1.74 (2H, m), 1.60 (1H, ddd, *J* = 14.2, 6.8, 2.4 Hz), 1.48 (2H, m), 1.41 (3H, s), 1.29 (3H, s), 1.01 (3H, s); ^13^C NMR (CD_3_OD, 151 MHz) δ 210.72, 172.79, 91.60, 77.57, 73.56, 45.08, 42.59, 41.29, 38.32, 36.63, 36.54, 32.18, 31.27, 29.52, 28.65, 26.51, 26.12, 24.88, 21.78; HRESIMS *m/z* 380.2429 [M+H]^+^ (calcd for C_21_H_34_N_1_O_5_, 380.2432).

#### (3S,4R,6aS,10aS)-7,7,10a-trimethyl-10-oxooctahydro-3H-3,6a-methanobenzo[c][1,2]dioxocin-4-yl 2-aminoacetate (29)

[α]^25^_D_ +23.6 (*c* 0.039, MeOH); UV (MeOH) *λ*max(log ε) 206 (3.34), 275 (3.11) nm; ^1^H NMR (CD_3_OD, 600 MHz) δ 5.00 (1H, ddd, *J* = 11.3, 7.6, 3.8 Hz), 4.23 (1H, td, *J* = 4.5, 2.1 Hz), 3.41 (1H, s), 3.35 (1H, s), 2.84 (1H, ddd, *J* = 15.5, 14.5, 6.8 Hz), 2.39 (1H, m), 2.33 (1H, ddd, *J* = 15.6, 4.8, 2.4 Hz), 2.22 (1H, m), 2.08 (1H, m), 2.03 (1H, dd, *J* = 14.7, 10.0 Hz), 1.98 (1H, m), 1.77 (1H, m), 1.74 (1H, m), 1.60 (1H, ddd, *J* = 14.2, 6.8, 2.4 Hz), 1.41 (3H, s), 1.29 (3H, S), 1.01 (3H, s); ^13^C NMR (CD_3_OD, 151 MHz) δ 192.39, 174.43, 91.52, 77.77, 73.82, 43.97, 42.59, 38.31, 36.64, 36.54, 31.36, 28.64, 26.53, 26.12, 24.90, 21.78; HRESIMS *m/z* 344.2074 [M+MeOH+H]^+^ (calcd for C_17_H_30_N_1_O_6_, 344.2067).

#### (3S,4R,6aS,10aS)-7,7,10a-trimethyl-10-oxooctahydro-3H-3,6a-methanobenzo[c][1,2]dioxocin-4-yl 2-(7-(dimethylamino)-2-oxo-2H-chromen-4-yl)acetate (30)

[α]^25^_D_ +38.1 (*c* 0.060, MeOH); UV (MeOH) *λ*max (log ε) 208 (3.72), 376 (3.44), 245 (3. 36) nm; ^1^H NMR (CD_3_OD, 500 MHz) δ 7.54 (1H, dd, *J* = 9.0, 1.3 Hz), 6.78 (1H, dd, *J* = 9.1, 2.6 Hz), 6.57 (1H, d, *J* = 2.6 Hz), 6.07 (1H, s), 4.99 (1H, ddd, *J* = 11.2, 7.6, 3.7 Hz), 4.58 (1H, s), 4.23 (1H, s), 3.08 (6H, s), 2.84 (1H, ddd, *J* = 15.7, 14.4, 6.9 Hz), 2.40 (1H, ddd, *J* = 18.6, 12.6, 6.3 Hz), 2.34 (1H, ddd, *J* = 15.6, 4.8, 2.4 Hz), 2.22 (1H, m), 2.05 (2H, m), 1.99 (2H, m), 1.73 (2H, m), 1.59 (1H, ddd, *J* = 14.3, 6.7, 2.4 Hz), 1.41 (3H, s), 1.28 (3H, s), 1.00 (3H, s); ^13^C NMR (CD_3_OD, 126 MHz) δ 207.92, 169.17, 161.84, 156.08, 153.10, 148.38, 125.49, 111.01, 109.31, 108.58, 98.47, 90.17, 75.89, 72.95, 41.44, 40.27, 38.16, 37.49, 35.88, 35.76, 30.53, 29.86, 27.45, 26.32, 25.40, 24.79, 21.60; HRESIMS *m/z* 484.2337 [M+H]^+^ (calcd for C_27_H_34_N_1_O_7_, 484.2330).

#### N-(2-(2-(7-(dimethylamino)-2-oxo-2H-chromen-4-yl)acetamido)ethyl)-3-phenylpropanamide (31)

See Figure S1.

## Supporting Information

Figure S1 **Synthesis of compound 31.** Synthetic scheme and experimental procedures for the synthesis of compound **31**.
(DOC)

Figure S2 **HPLC and NMR experimental data.** ^1^H NMR spectra and HPLC purity traces for compounds **8–30**.
(PDF)

Table S1 **Tabulated purity of merulin library.** Calculated compound purities for compounds **8–30**.
(XLSX)

Table S2 **Tabulated parameters for QED measurements.** Qualitative estimate of drug-likeness scores for synthetic and reference compounds.
(XLS)
