# Supplementary material for: Hit-to-Lead Development of the Chamigrane Endoperoxide Merulin A for the Treatment of African Sleeping Sickness
Source: PLoS One. 2012 Sep 27;7(9):e46172. doi: 10.1371/journal.pone.0046172 (PMC3459870; doi:10.1371/journal.pone.0046172)
Supplement: Figure S1 — Synthesis of compound 31. Synthetic scheme and experimental procedures for the synthesis of compound 31. (DOC) [file pone.0046172.s001.doc]

**Synthesis of coumarin control 31**

**tert-butyl-(2-(2-(7-(dimethylamino)-2-oxo-2H-chromen-4-yl)acetamido)ethyl)carbamate (S1):** To a solution of 7-Dimethylaminocoumarin-4-acetic acid (12.7 mg, 0.051 mmol) in DMF (10 mL) was added *N*-Boc-ethylenediamine (8.64 mg, 0.054 mmol), PyBOP (20.8 mg, 0.040 mmol), and DIPEA (67 µL, 0.411 mmol) and the resulting solution stirred for 12 hrs. The crude reaction mixture was concentrated to dryness *in vacuo*, and the crude solid purified using reverse-phase HPLC (60:40 MeOH:H_2_O + 0.02% HCOOH, Phenomenex Synergi C_18_ 250 x 10 mm column, 10μm, 2 mL/min), to give primary amine **S1** as a white solid (11.9 mg, 60% yield). UV (MeOH) λ_max_ (log ε): 209 nm (4.30), 247 nm (4.02), 375 nm (4.14) ; ^1^H NMR (CD­_3_OD, 600 MHz) δ 7.557 (d, J = 9 Hz, 1H), 6.764 (d, J = 9 Hz, 1H), 6.564 (d, J = 2.4 Hz, 1H), 6.047 (s, 1H), 3.685 (s, 2H), 3.349 (s, 1H), 3.072 (m, 8H), 1.423 (s, 9H) ; ^13^C NMR (CD_3_OD, 150 MHz) δ 28.8, 40.36, 40.95, 40.99, 80.33, 98.92, 110.00, 110.66, 110.77, 127.06, 152.89, 154.99, 157.34, 164.48; HRESIMS *m/z* [M+Na]^+^ 412.1847 (calcd for C_20_H_27_O_5_N_3_Na, 412.1848).

**(2-(2-(7-(dimethylamino)-2-oxo-2H-chromen-4-yl)acetamido)ethyl)carbamic acid (S2):** A solution of **S1** (5.0 mg, 0.013 mmol) in 1:1 TFA/H20 (2 mL) was stirred for 30 minutes at room temperature. TLC showed consumption of starting material. The reaction mixture was concentrated to dryness *in vacuo* to give **S2** as a yellow oil (100% yield). UV (MeOH) λ_max_ (log ε): 208 nm (4.10), 246 nm (3.81), 375 nm (3.94); ^1^H NMR (CD_3_OD, 600 MHz) δ 7.545 (d, J = 9 Hz, 1H), 6.754 (dd, J = 9 Hz, 2.4 Hz, 1H), 6.553 (d, J = 2.4 Hz, 1H), 6.045 (s, 1H), 3.735 (s, 2H), 3.479 (t, J = 5.4 Hz, 2H), 3.066 (m, 8H) ; ^13^C NMR (CD_3_OD, 150 MHz) δ 38.62, 39.96, 40.34, 40.84, 98.91, 109.93, 110.62, 110.73, 127.04, 152.55, 155.00, 157.34, 164.42, 172.55; HRESIMS *m/z* [M+H]^+^ 290.1503 (calcd for C_15_H_20_O_3_N_3_, 290.1504).

**N-(2-(2-(7-(dimethylamino)-2-oxo-2H-chromen-4-yl)acetamido)ethyl)-3-phenylpropanamide (31):** A solution of **2** (1.79 mg, 0.00692 mmol) in of DCM (1 mL) was added to a pre-stirred solution of hydrocinnamic acid (2.58 mg, 0.017 mmol), PyBOP (5.86 µmol), and DIPEA (19 µL, 0.111 mmol) in DCM (1 mL) for 12 hrs. Reaction completion was verified by TLC. Reaction mixture was concentrated *in vacuo* and purified by RP-HPLC (60% MeOH, 40% H_2_O + 0.02% HCOOH, Phenomenex Jupiter C18 250 x 4.6 mm column, 5μm, 1 mL/min) to give **31** as a yellow solid (0.99 mg, 34%). UV (MeOH) λ_max_ (log ε): 202 nm (4.01), 245 nm (2.44), 376 nm (3.48) ; ^1^H NMR (CDCl_3_, 600 MHz) δ 7.435 (d, J = 9Hz, 1H), 7.301-7.253 (m, 1H), 7.230-7.171 (m, 2H), 7.154 (d, J = 7.2 Hz, 2H), 6.591 (dd, J = 9 Hz, 2.4 Hz, 1H), 6.485 (d, J = 2.4 Hz, 1H), 6.036 (s, 1H), 5.859 (bs, 1H), 3.562 (s, 2H), 3.004 (s, 6H), 2.964 (t, J = 7.8 Hz, 2H), 2.867 (t, J = 7.8 Hz, 2H), 2.682 (bs, 1H), 2.375 (t, J = 7.8 Hz, 2H), 1.465 (t, J = 7.8 Hz, 2H) ; ^13^C NMR (CDCl_3_, 150 MHz) δ 12.35, 26.53, 26.58, 31.68, 40.15, 46.47, 54.94, 98.40, 108.46, 109.24, 110.67, 110.79, 126.42, 126.44, 128.39, 128.47, 128.54, 128.60, 128.63, 128.70, 149.51, 153.20, 156.21, 169.12; HRESIMS *m/z* [M+Na]^+^ 444.1897 (calcd for C_24_H_27_O_4_N_3_Na, 444.1894).
