# Supplementary material for: Hit-to-Lead Development of the Chamigrane Endoperoxide Merulin A for the Treatment of African Sleeping Sickness
Source: PLoS One. 2012 Sep 27;7(9):e46172. doi: 10.1371/journal.pone.0046172 (PMC3459870; doi:10.1371/journal.pone.0046172)
Supplement: Figure S2 — HPLC and NMR experimental data. 1H NMR spectra and HPLC purity traces for compounds 8–30. (PDF) [file pone.0046172.s002.pdf]

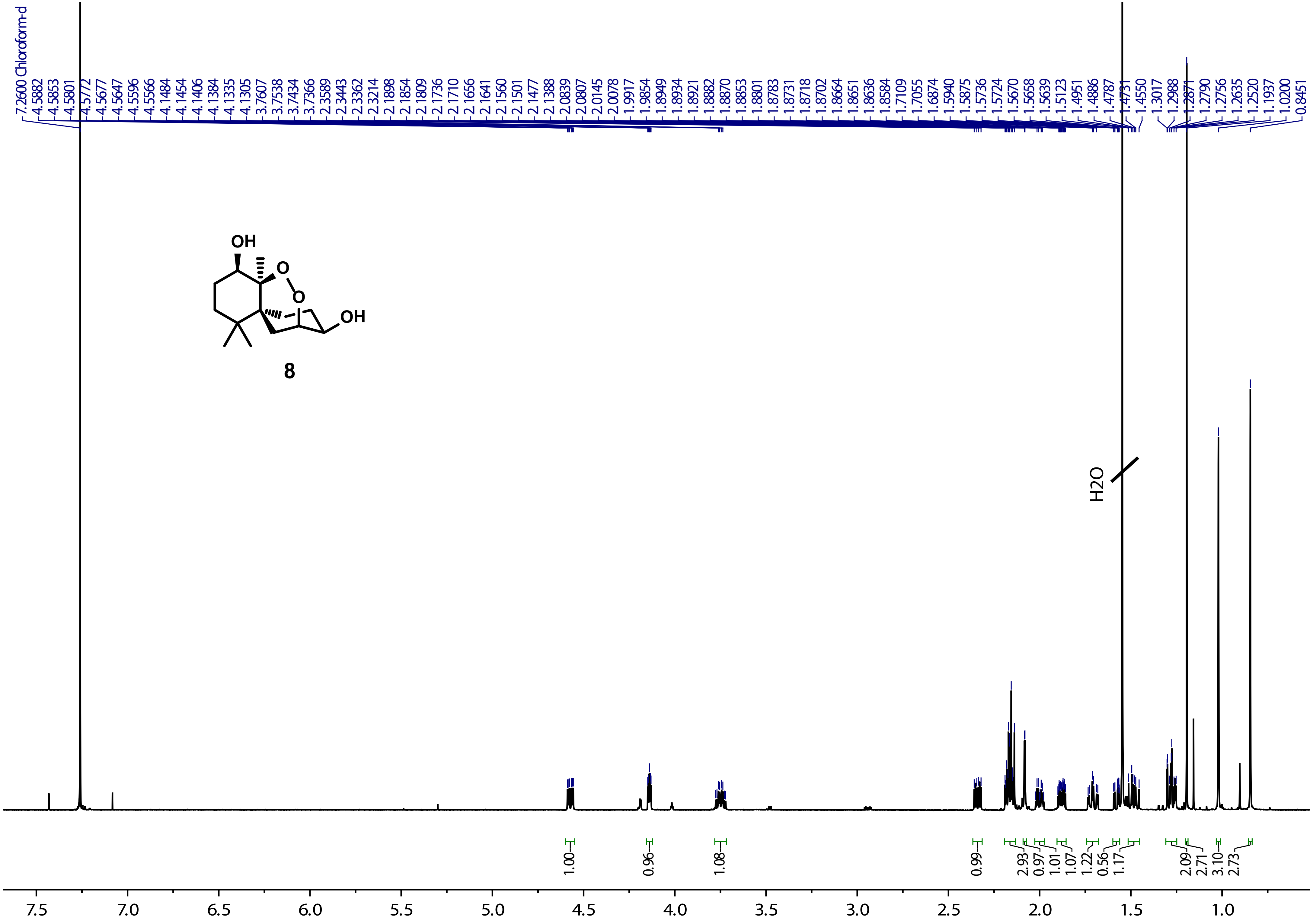

7.2600 Chloroform-d
4.5882
4.5853
4.5801
4.5772
4.5677
4.5647
4.5596
4.5566
4.1484
4.1454
4.1406
4.1384
4.1335
4.1305
3.7607
3.7538
3.7434
3.7366
2.3589
2.3443
2.3362
2.3214
2.1898
2.1854
2.1809
2.1736
2.1710
2.1656
2.1641
2.1560
2.1501
2.1477
2.1388
2.0839
2.0807
2.0145
2.0078
1.9917
1.9854
1.8949
1.8934
1.8921
1.8882
1.8870
1.8853
1.8801
1.8783
1.8731
1.8718
1.8702
1.8664
1.8651
1.8636
1.8584
1.7109
1.7055
1.6874
1.5940
1.5875
1.5736
1.5724
1.5670
1.5658
1.5639
1.5123
1.4951
1.4886
1.4787
1.4731
1.4550
1.3017
1.2988
1.2871
1.2790
1.2756
1.2635
1.2520
1.1937
1.0200
0.8451
OH
O
O
OH
8
H2O
1.00
0.96
1.08
0.99
2.93
0.97
1.01
1.07
1.22
0.56
1.17
2.09
2.71
3.10
2.73
7.5
7.0
6.5
6.0
5.5
5.0
4.5
4.0
3.5
3.0
2.5
2.0
1.5
1.0

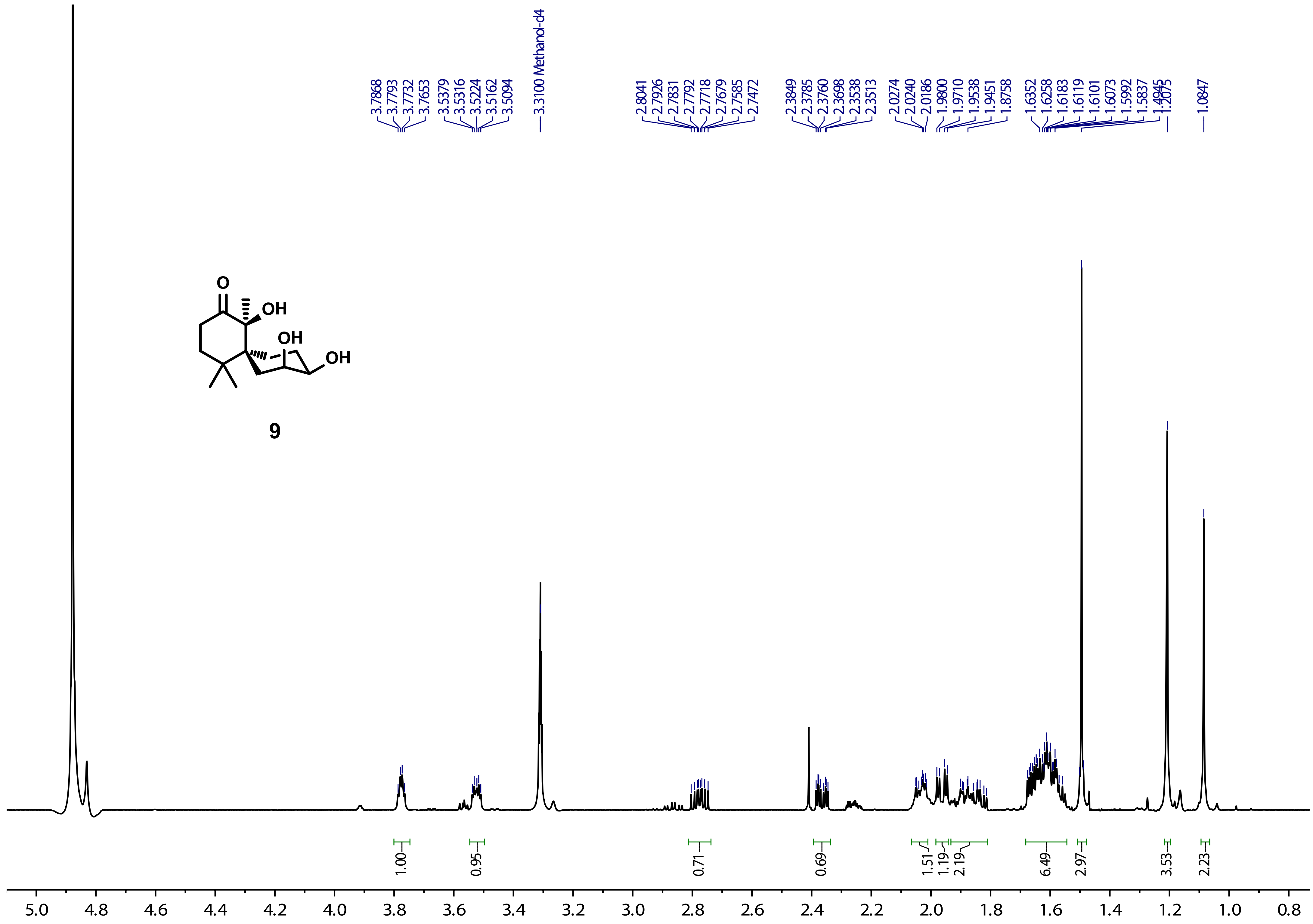

3.7868
3.7793
3.7732
3.7653
3.5379
3.5316
3.5224
3.5162
3.5094
3.3100 Methanol-d4
2.8041
2.7926
2.7831
2.7792
2.7718
2.7679
2.7585
2.7472
2.3849
2.3785
2.3760
2.3698
2.3538
2.3513
2.0274
2.0240
2.0186
1.9800
1.9710
1.9538
1.9451
1.8758
1.6352
1.6258
1.6183
1.6119
1.6101
1.6073
1.5992
1.5837
1.4945
1.2075
1.0847
O
OH
OH
OH
9
1.00
0.95
0.71
0.69
1.51
1.19
2.19
6.49
2.97
3.53
2.23
5.0
4.8
4.6
4.4
4.2
4.0
3.8
3.6
3.4
3.2
3.0
2.8
2.6
2.4
2.2
2.0
1.8
1.6
1.4
1.2
1.0
0.8

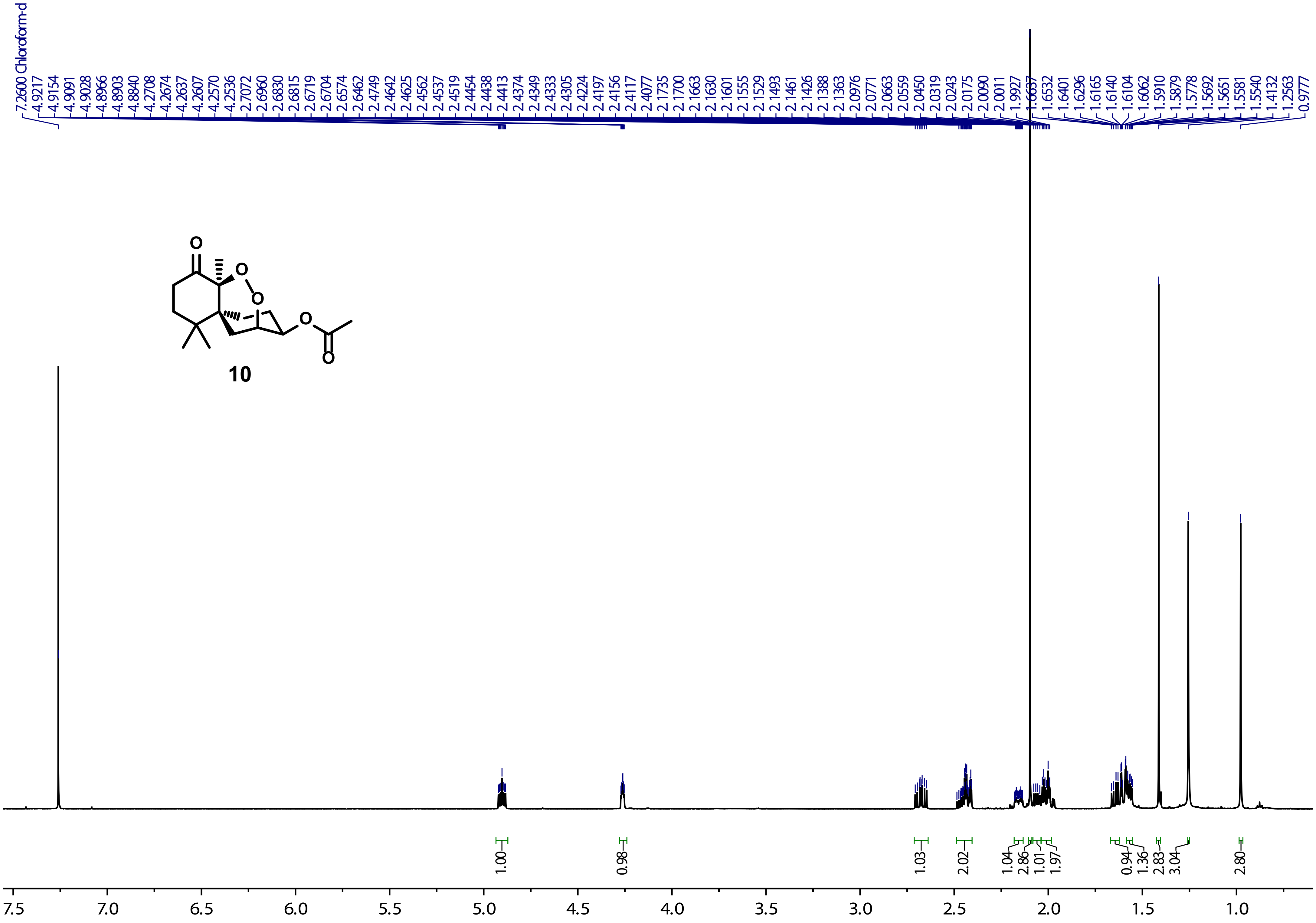
7.2600 Chloroform-d
4.9217
4.9154
4.9091
4.9028
4.8966
4.8903
4.8840
4.2708
4.2674
4.2637
4.2607
4.2570
4.2536
2.7072
2.6960
2.6830
2.6815
2.6719
2.6704
2.6574
2.6462
2.4749
2.4642
2.4625
2.4562
2.4537
2.4519
2.4454
2.4438
2.4413
2.4374
2.4349
2.4333
2.4305
2.4224
2.4197
2.4156
2.4117
2.4077
2.1735
2.1700
2.1663
2.1630
2.1601
2.1555
2.1529
2.1493
2.1461
2.1426
2.1388
2.1363
2.0976
2.0771
2.0663
2.0559
2.0450
2.0319
2.0243
2.0175
2.0090
2.0011
1.9927
1.6637
1.6532
1.6401
1.6296
1.6165
1.6140
1.6104
1.6062
1.5910
1.5879
1.5778
1.5692
1.5651
1.5581
1.5540
1.4132
1.2563
0.9777
O
O
O
O
O
10
1.00
0.98
1.03
2.02
1.04
2.86
1.01
1.97
0.94
1.36
2.83
3.04
2.80
7.5
7.0
6.5
6.0
5.5
5.0
4.5
4.0
3.5
3.0
2.5
2.0
1.5
1.0

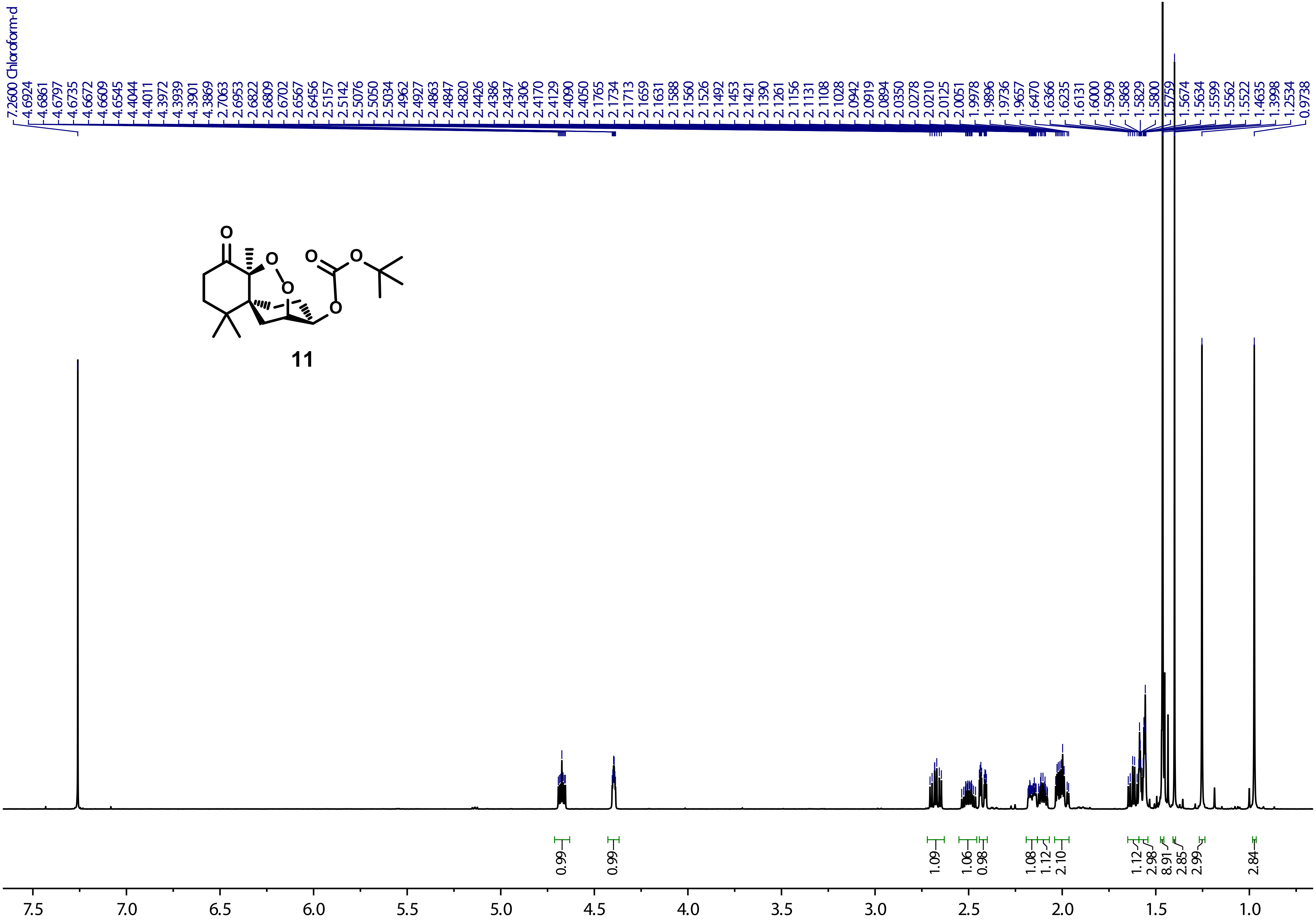

7.2600 Chloroform-d
4.6924
4.6861
4.6797
4.6735
4.6672
4.6609
4.6545
4.4044
4.4011
4.3972
4.3939
4.3901
4.3869
2.7063
2.6953
2.6822
2.6809
2.6702
2.6567
2.6456
2.5157
2.5142
2.5076
2.5050
2.5034
2.4962
2.4927
2.4863
2.4847
2.4820
2.4426
2.4386
2.4347
2.4306
2.4170
2.4129
2.4090
2.4050
2.1765
2.1734
2.1713
2.1659
2.1631
2.1588
2.1560
2.1526
2.1492
2.1453
2.1421
2.1390
2.1261
2.1156
2.1131
2.1108
2.1028
2.0942
2.0919
2.0894
2.0350
2.0278
2.0210
2.0125
2.0051
1.9978
1.9896
1.9736
1.9657
1.6470
1.6366
1.6235
1.6131
1.6000
1.5909
1.5868
1.5829
1.5800
1.5759
1.5674
1.5634
1.5599
1.5562
1.5522
1.4635
1.3998
1.2534
0.9738
11
0.99
0.99
1.09
1.06
0.98
1.08
1.12
2.10
1.12
2.98
8.91
2.85
2.99
2.84
7.5
7.0
6.5
6.0
5.5
5.0
4.5
4.0
3.5
3.0
2.5
2.0
1.5
1.0

Current Chromatogram(s)

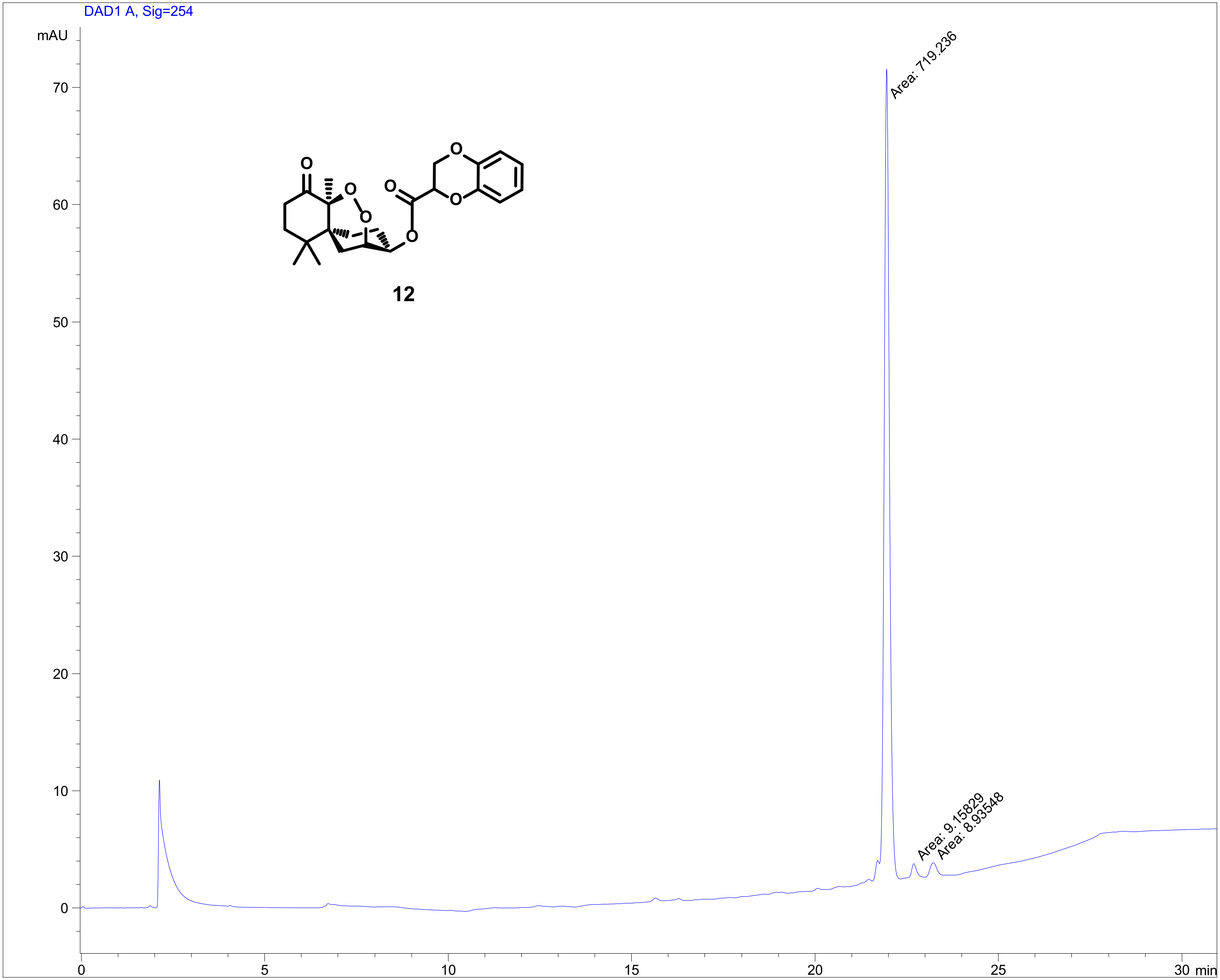

12

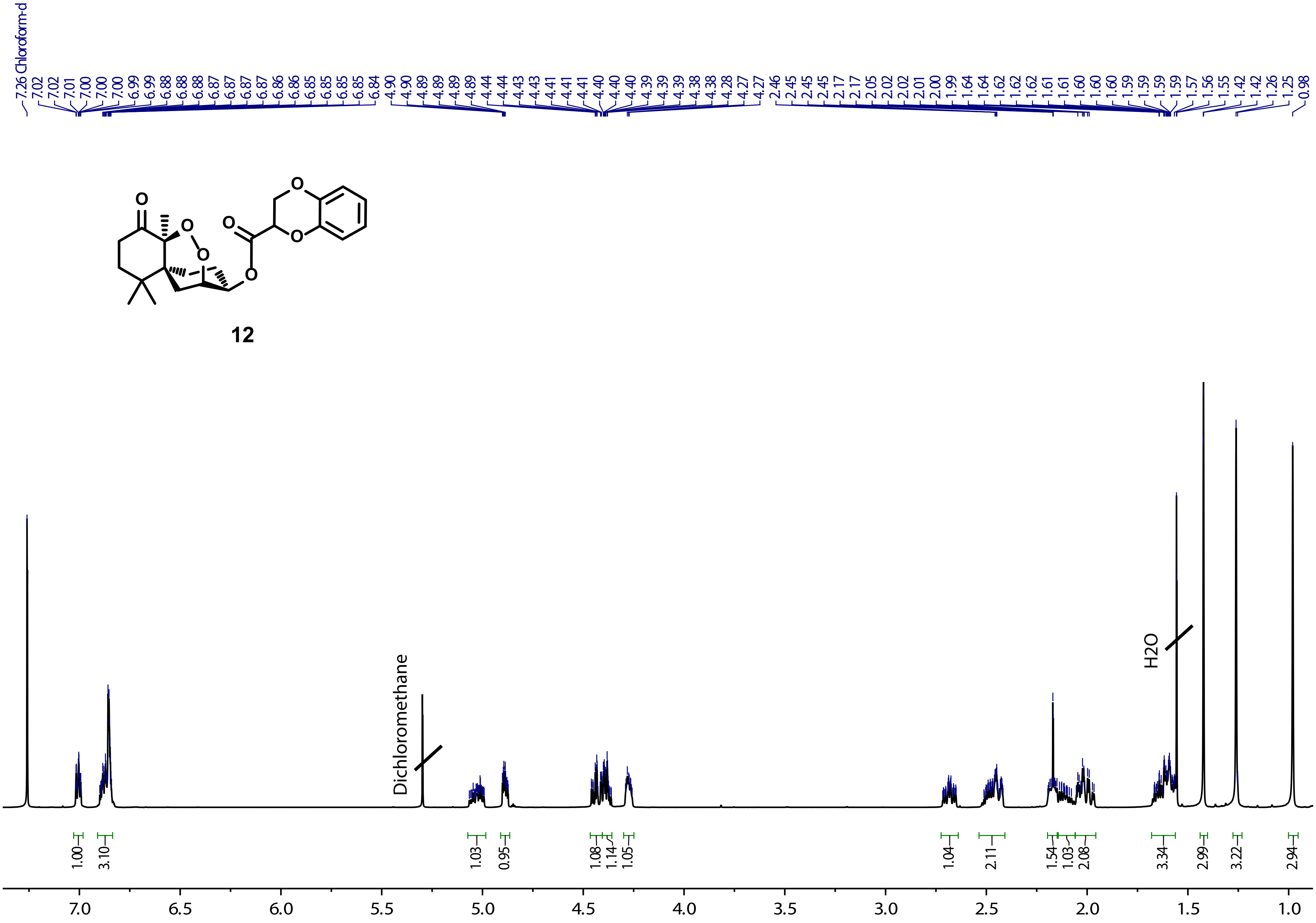
7.26 Chloroform-d
7.02 7.02 7.01 7.00 7.00 7.00 6.99 6.99 6.88 6.88 6.88 6.87 6.87 6.87 6.86 6.86 6.85 6.85 6.85 6.84
4.90 4.90 4.89 4.89 4.89 4.89 4.44 4.44 4.43 4.43 4.41 4.41 4.41 4.40 4.40 4.40 4.39 4.39 4.38 4.38 4.28 4.27 4.27
2.46 2.45 2.45 2.45 2.17 2.17 2.05 2.02 2.02 2.01 2.00 1.99 1.64 1.64 1.62 1.62 1.62 1.61 1.61 1.60 1.60 1.60 1.59 1.59 1.59 1.59 1.57 1.56 1.55 1.42 1.42 1.26 1.25 0.98
12
Dichloromethane
H2O
1.00 3.10 1.03 0.95 1.08 1.14 1.05 1.04 2.11 1.54 1.03 2.08 3.34 2.99 3.22 2.94
7.0 6.5 6.0 5.5 5.0 4.5 4.0 3.5 3.0 2.5 2.0 1.5 1.0

Current Chromatogram(s)

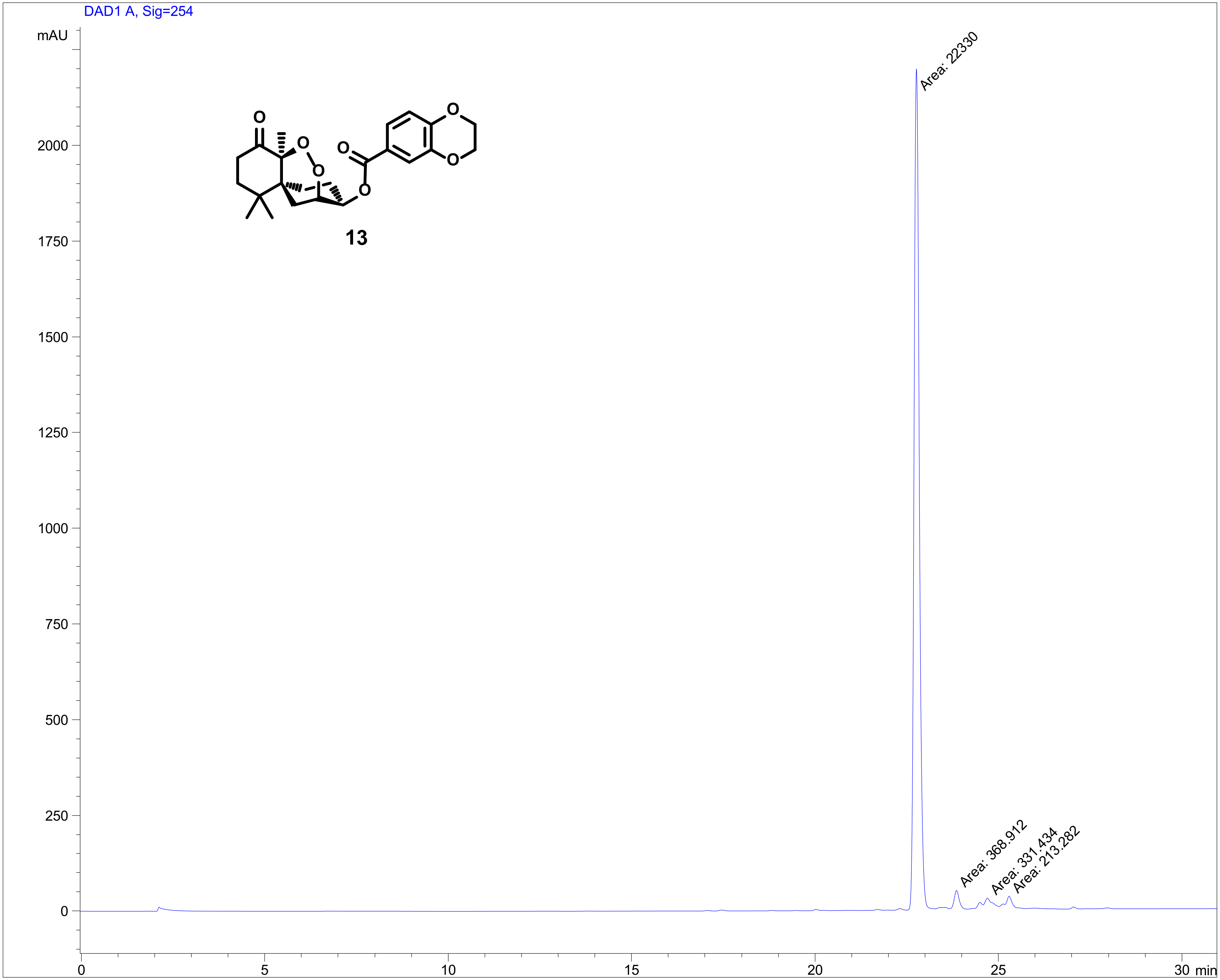

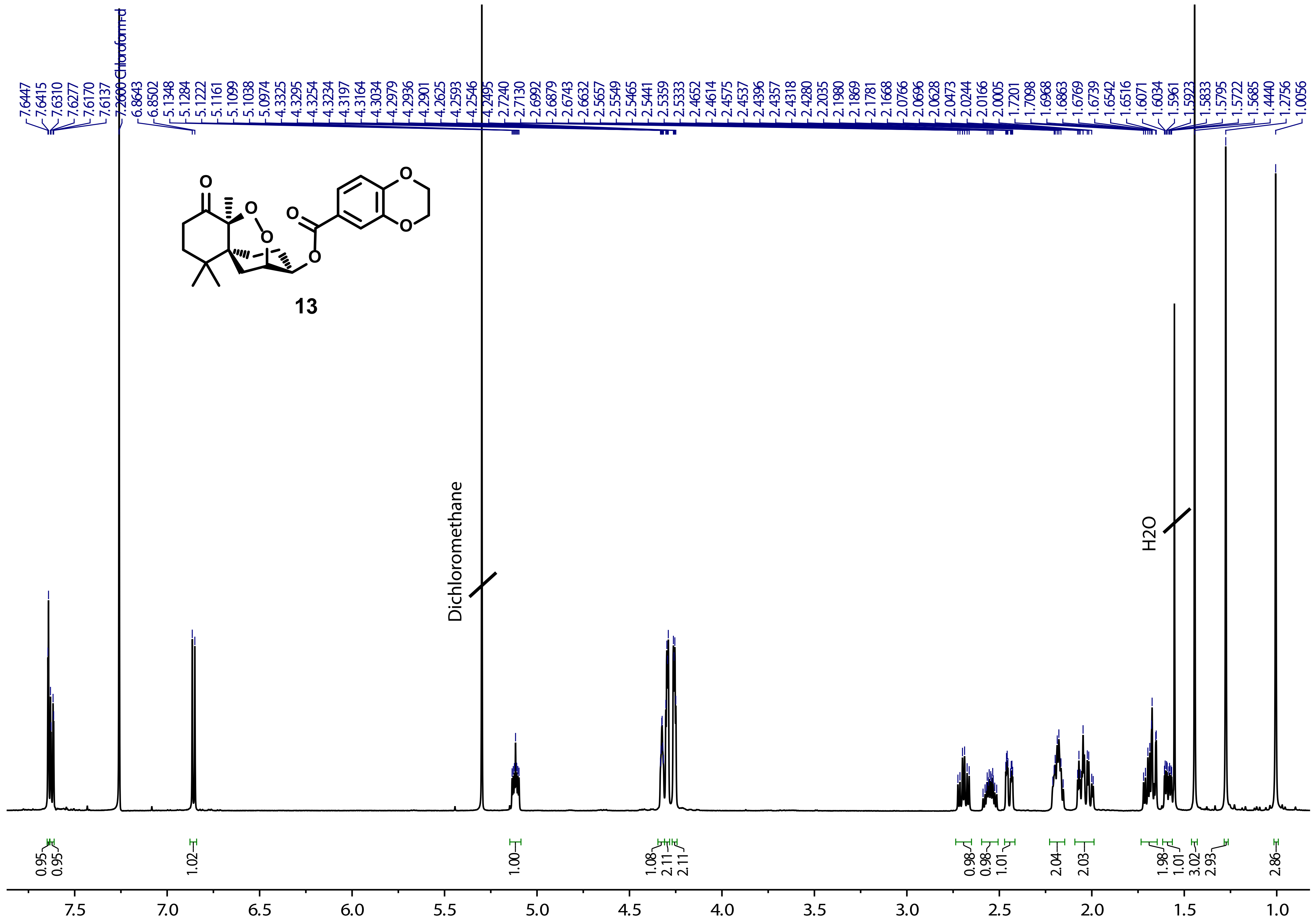

7.6447
7.6415
7.6310
7.6277
7.6170
7.6137
7.2600 Chloroform-d
6.8643
6.8502
5.1348
5.1284
5.1222
5.1161
5.1099
5.1038
5.0974
4.3325
4.3295
4.3254
4.3234
4.3197
4.3164
4.3034
4.2979
4.2936
4.2901
4.2625
4.2593
4.2546
4.2495
2.7240
2.7130
2.6992
2.6879
2.6743
2.6632
2.5657
2.5549
2.5465
2.5441
2.5359
2.5333
2.4652
2.4614
2.4575
2.4537
2.4396
2.4357
2.4318
2.4280
2.2035
2.1980
2.1869
2.1781
2.1668
2.0766
2.0696
2.0628
2.0473
2.0244
2.0166
2.0005
1.7201
1.7098
1.6968
1.6863
1.6769
1.6739
1.6542
1.6516
1.6071
1.6034
1.5961
1.5923
1.5833
1.5795
1.5722
1.5685
1.4440
1.2756
1.0056
13
Dichloromethane
H2O
0.95
0.95
1.02
1.00
1.08
2.11
2.11
0.98
0.98
1.01
2.04
2.03
1.98
1.01
3.02
2.93
2.86
7.5
7.0
6.5
6.0
5.5
5.0
4.5
4.0
3.5
3.0
2.5
2.0
1.5
1.0

Current Chromatogram(s)

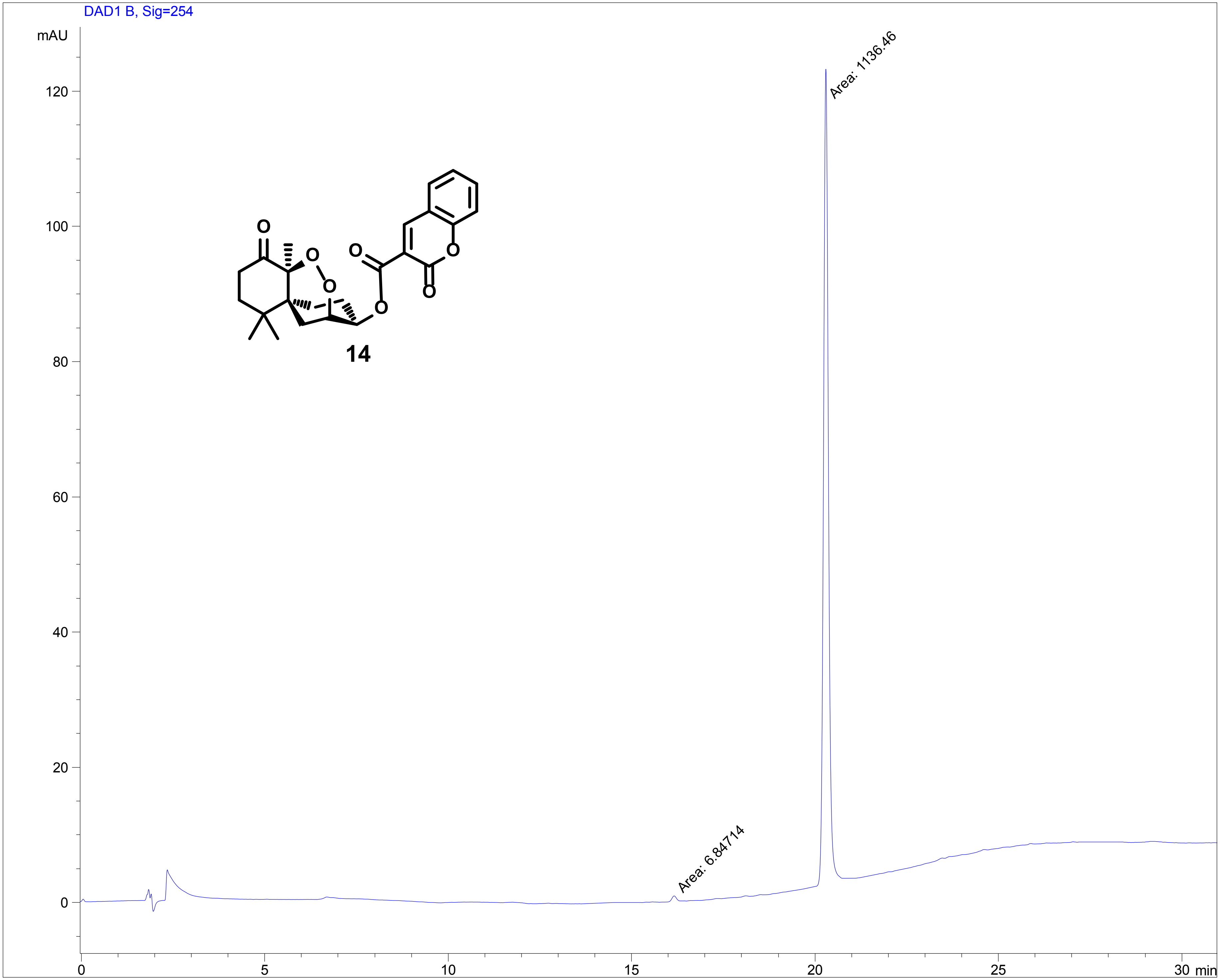

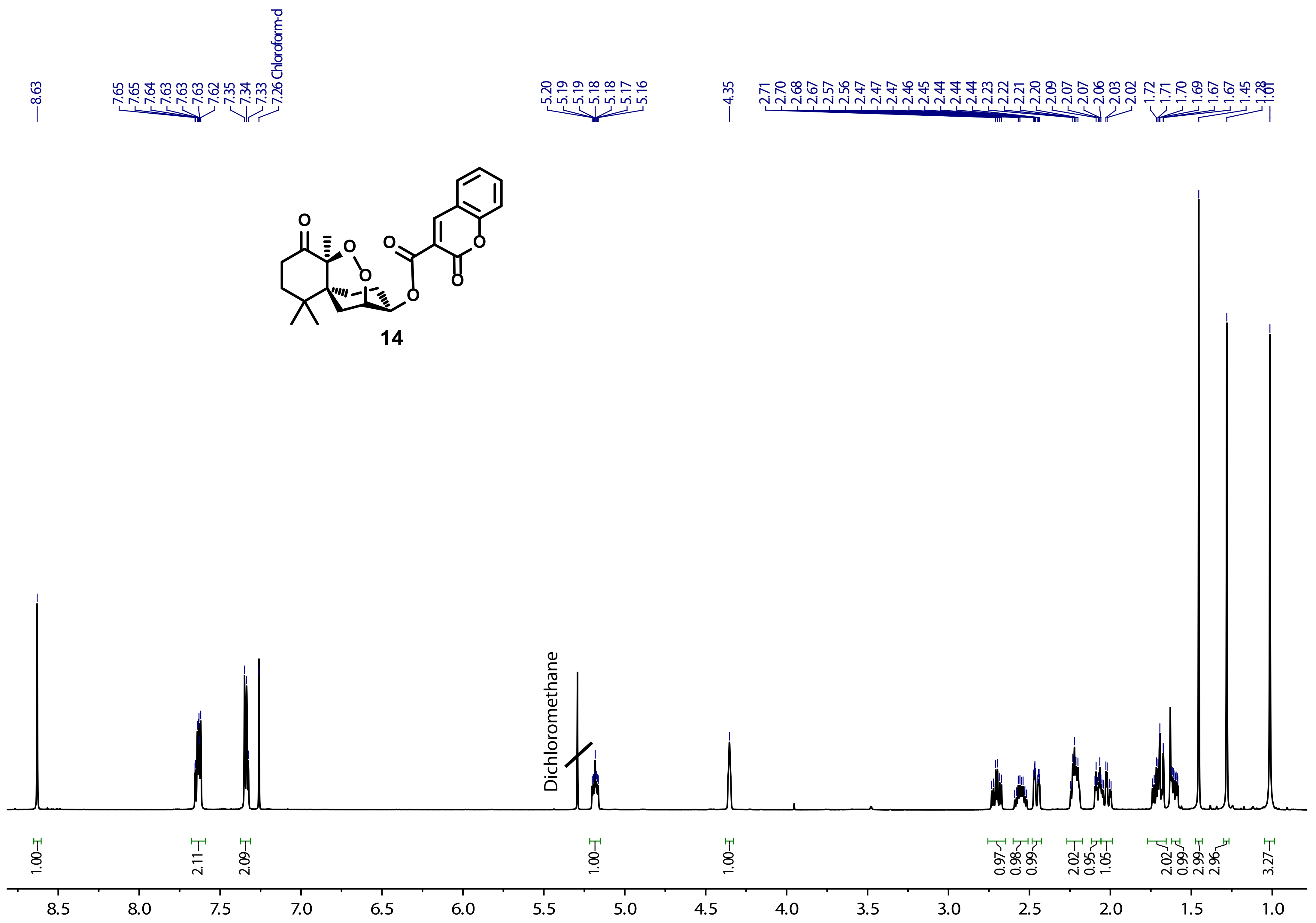
8.63
7.65
7.65
7.64
7.63
7.63
7.63
7.62
7.35
7.34
7.33
7.26 Chloroform-d
5.20
5.19
5.19
5.18
5.18
5.17
5.16
4.35
2.71
2.70
2.68
2.67
2.57
2.56
2.47
2.47
2.47
2.46
2.45
2.44
2.44
2.44
2.23
2.22
2.21
2.20
2.09
2.07
2.07
2.06
2.03
2.02
1.72
1.71
1.70
1.69
1.67
1.67
1.45
1.28
1.01
14
Dichloromethane
1.00
2.11
2.09
1.00
1.00
0.97
0.98
0.99
2.02
0.95
1.05
2.02
0.99
2.99
2.96
3.27
8.5
8.0
7.5
7.0
6.5
6.0
5.5
5.0
4.5
4.0
3.5
3.0
2.5
2.0
1.5
1.0

Current Chromatogram(s)

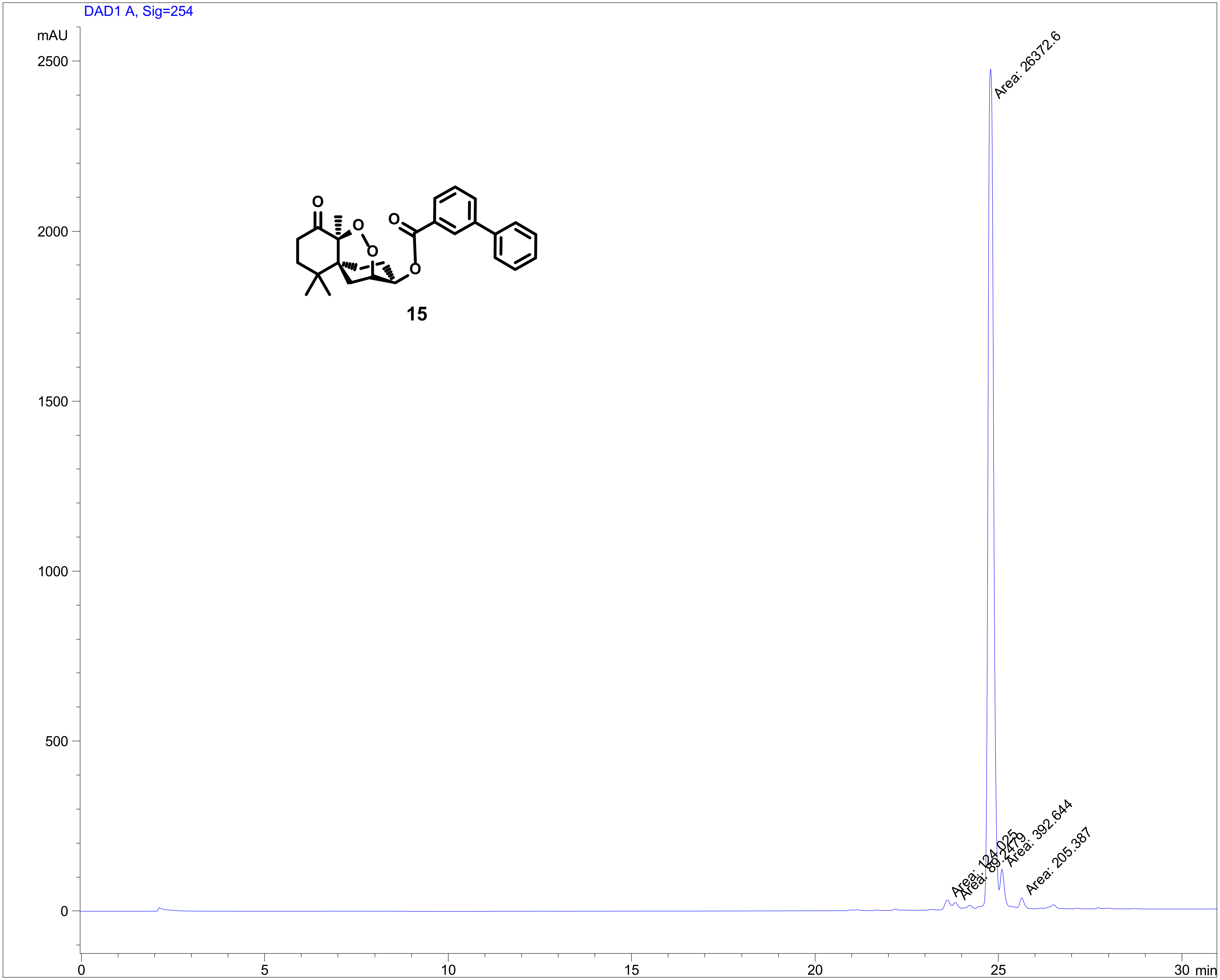

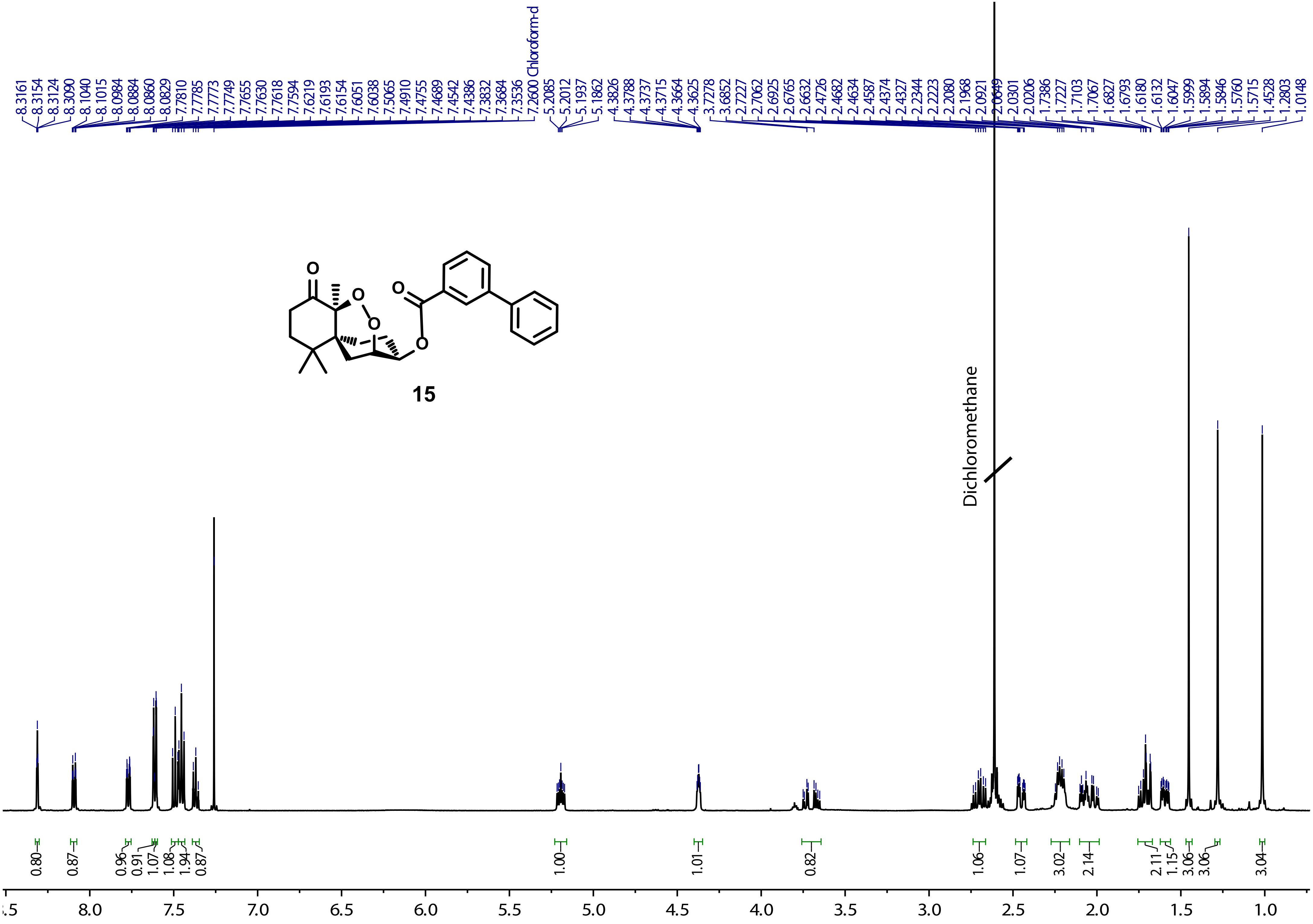

8.3161
8.3154
8.3124
8.3090
8.1040
8.1015
8.0984
8.0884
8.0860
8.0829
7.7810
7.7785
7.7773
7.7749
7.7655
7.7630
7.7618
7.7594
7.6219
7.6193
7.6154
7.6051
7.6038
7.5065
7.4910
7.4755
7.4689
7.4542
7.4386
7.3832
7.3684
7.3536
7.2600 Chloroform-d
5.2085
5.2012
5.1937
5.1862
4.3826
4.3788
4.3737
4.3715
4.3664
4.3625
3.7278
3.6852
2.7227
2.7062
2.6925
2.6765
2.6632
2.4726
2.4682
2.4634
2.4587
2.4374
2.4327
2.2344
2.2223
2.2080
2.1968
2.0921
2.0649
2.0301
2.0206
1.7386
1.7227
1.7103
1.7067
1.6827
1.6793
1.6180
1.6132
1.6047
1.5999
1.5894
1.5846
1.5760
1.5715
1.4528
1.2803
1.0148
15
Dichloromethane
0.80
0.87
0.96
0.91
1.07
1.08
1.94
0.87
1.00
1.01
0.82
1.06
1.07
3.02
2.14
2.11
1.15
3.06
3.06
3.04
8.0
7.5
7.0
6.5
6.0
5.5
5.0
4.5
4.0
3.5
3.0
2.5
2.0
1.5
1.0

Current Chromatogram(s)

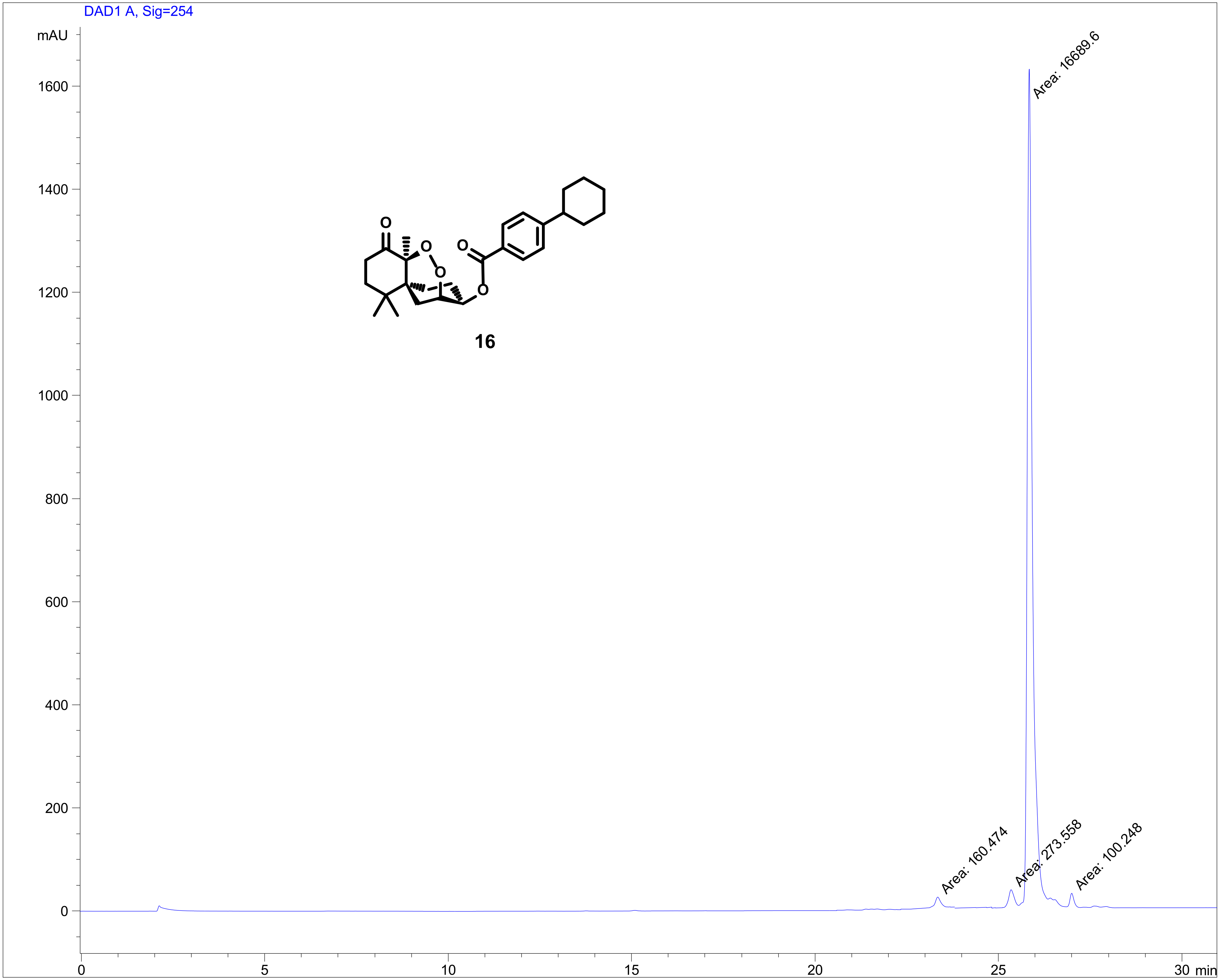

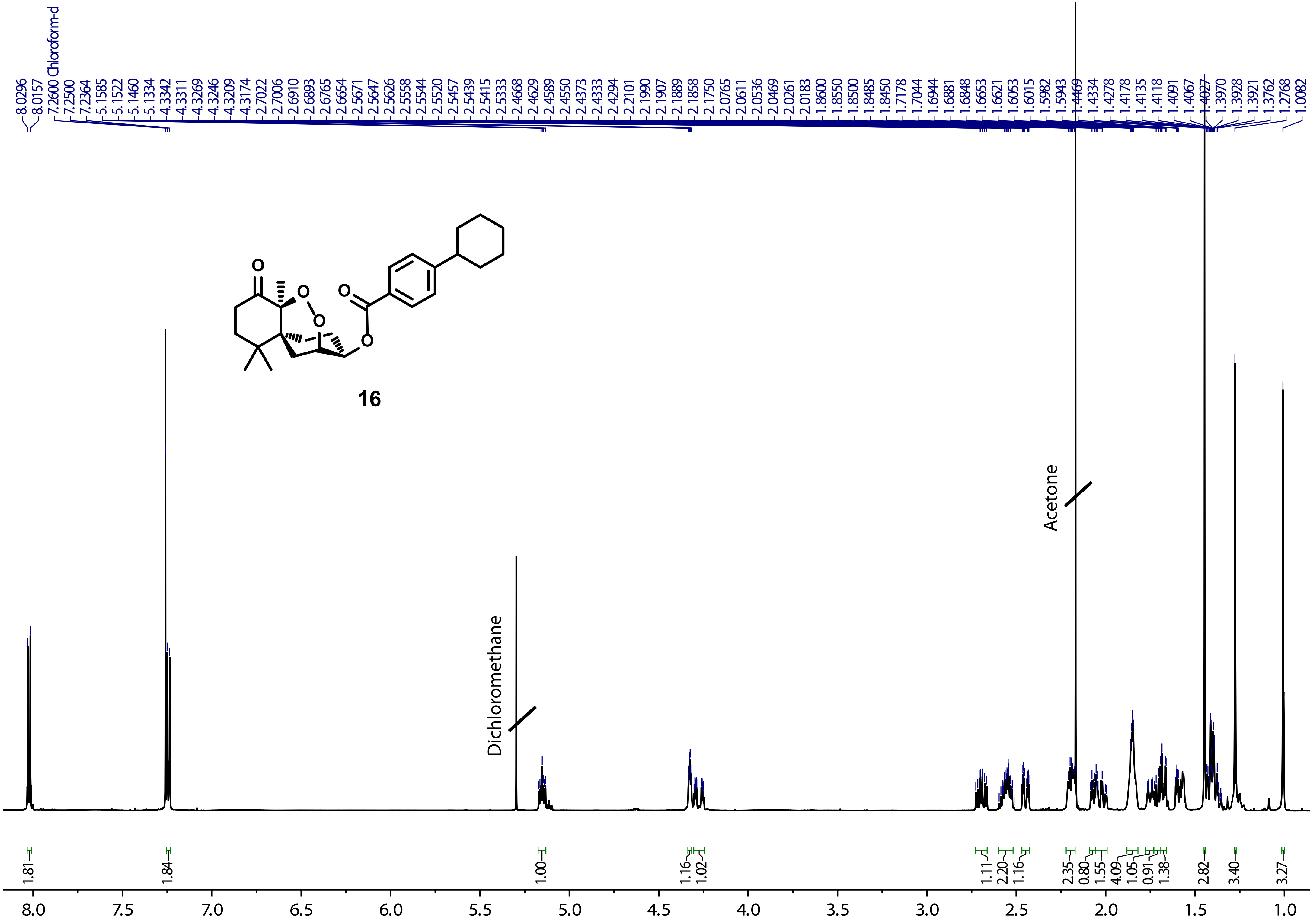

Chloroform-d
Dichloromethane
Acetone
16

Current Chromatogram(s)

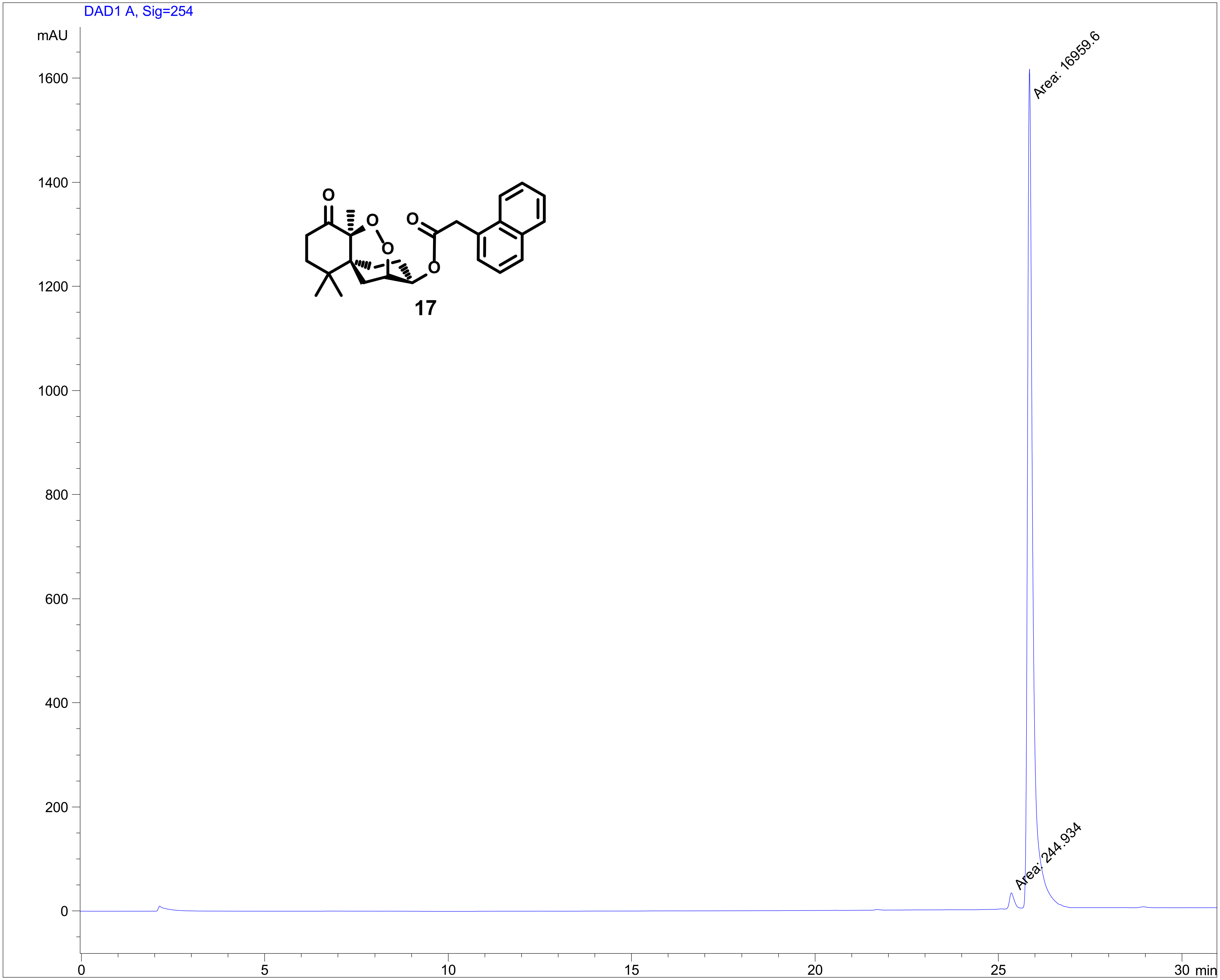

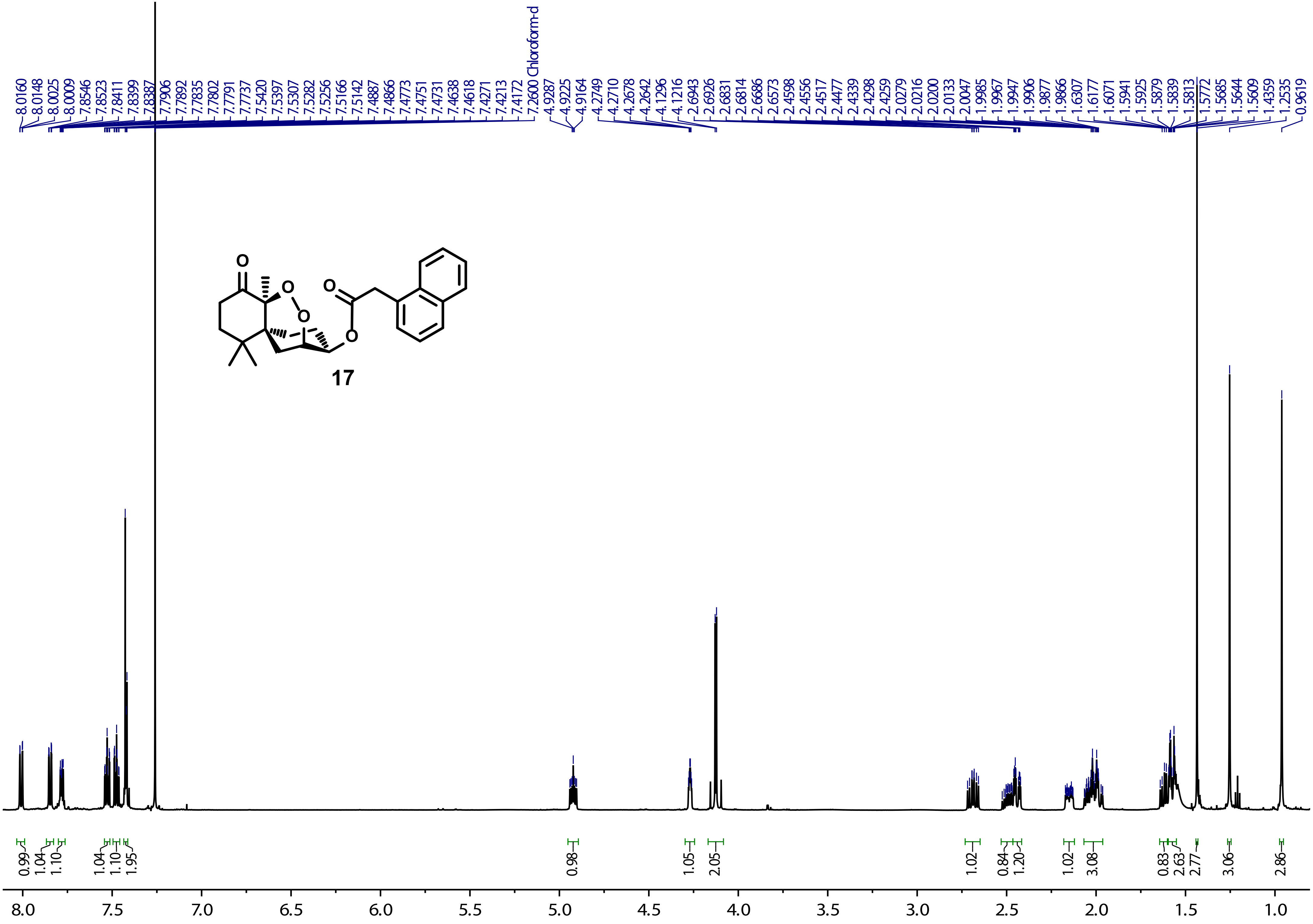
8.0160
8.0148
8.0025
8.0009
7.8546
7.8523
7.8411
7.8399
7.8387
7.7906
7.7892
7.7835
7.7802
7.7791
7.7737
7.5420
7.5397
7.5307
7.5282
7.5256
7.5166
7.5142
7.4887
7.4866
7.4773
7.4751
7.4731
7.4638
7.4618
7.4271
7.4213
7.4172
7.2600 Chloroform-d
4.9287
4.9225
4.9164
4.2749
4.2710
4.2678
4.2642
4.1296
4.1216
2.6943
2.6926
2.6831
2.6814
2.6686
2.6573
2.4598
2.4556
2.4517
2.4477
2.4339
2.4298
2.4259
2.0279
2.0216
2.0200
2.0133
2.0047
1.9985
1.9967
1.9947
1.9906
1.9877
1.9866
1.6307
1.6177
1.6071
1.5941
1.5925
1.5879
1.5839
1.5813
1.5772
1.5685
1.5644
1.5609
1.4359
1.2535
0.9619
O
O
O
O
O
17
0.99
1.04
1.10
1.04
1.10
1.95
0.98
1.05
2.05
1.02
0.84
1.20
1.02
3.08
0.83
2.63
2.77
3.06
2.86
8.0
7.5
7.0
6.5
6.0
5.5
5.0
4.5
4.0
3.5
3.0
2.5
2.0
1.5
1.0

Current Chromatogram(s)

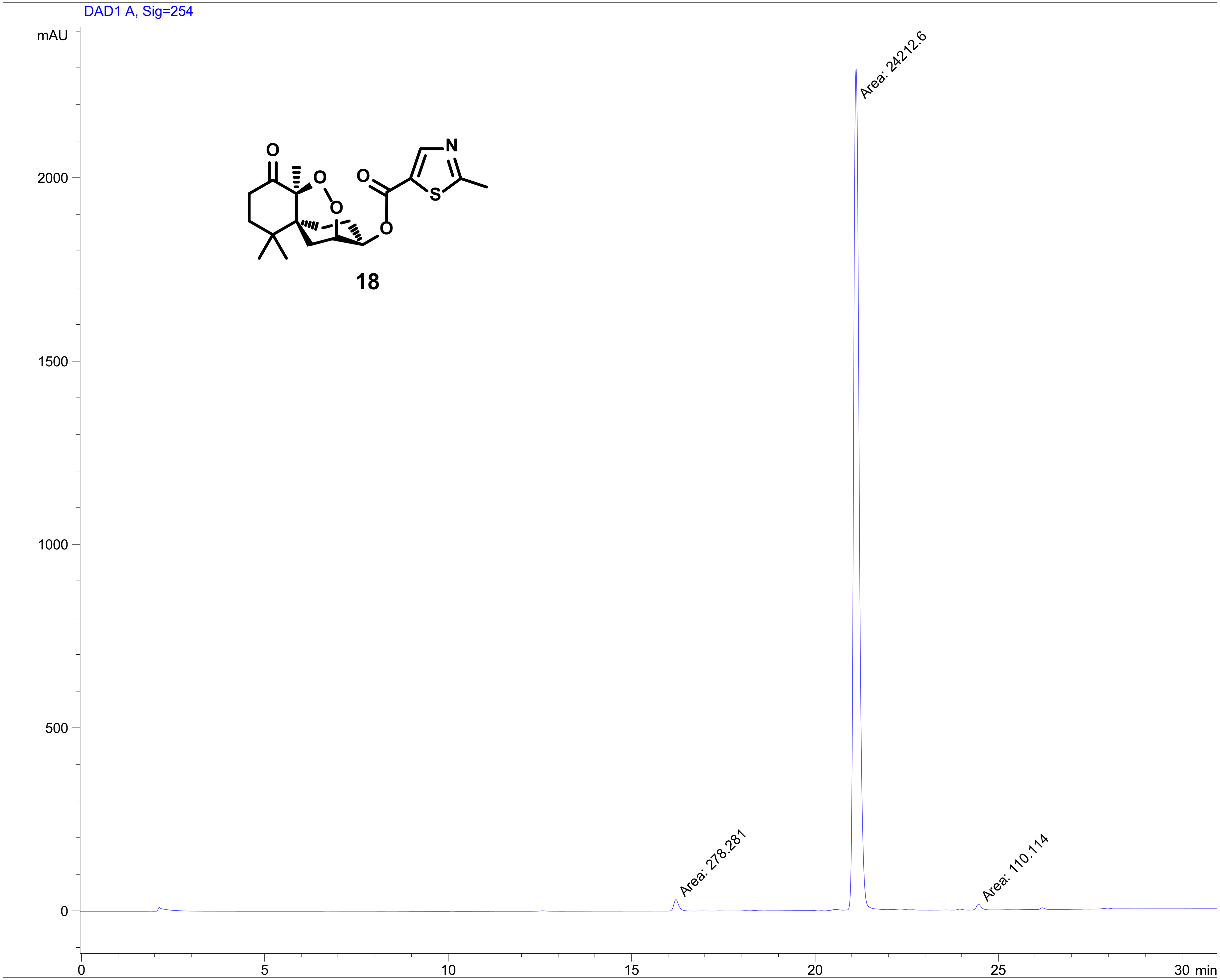

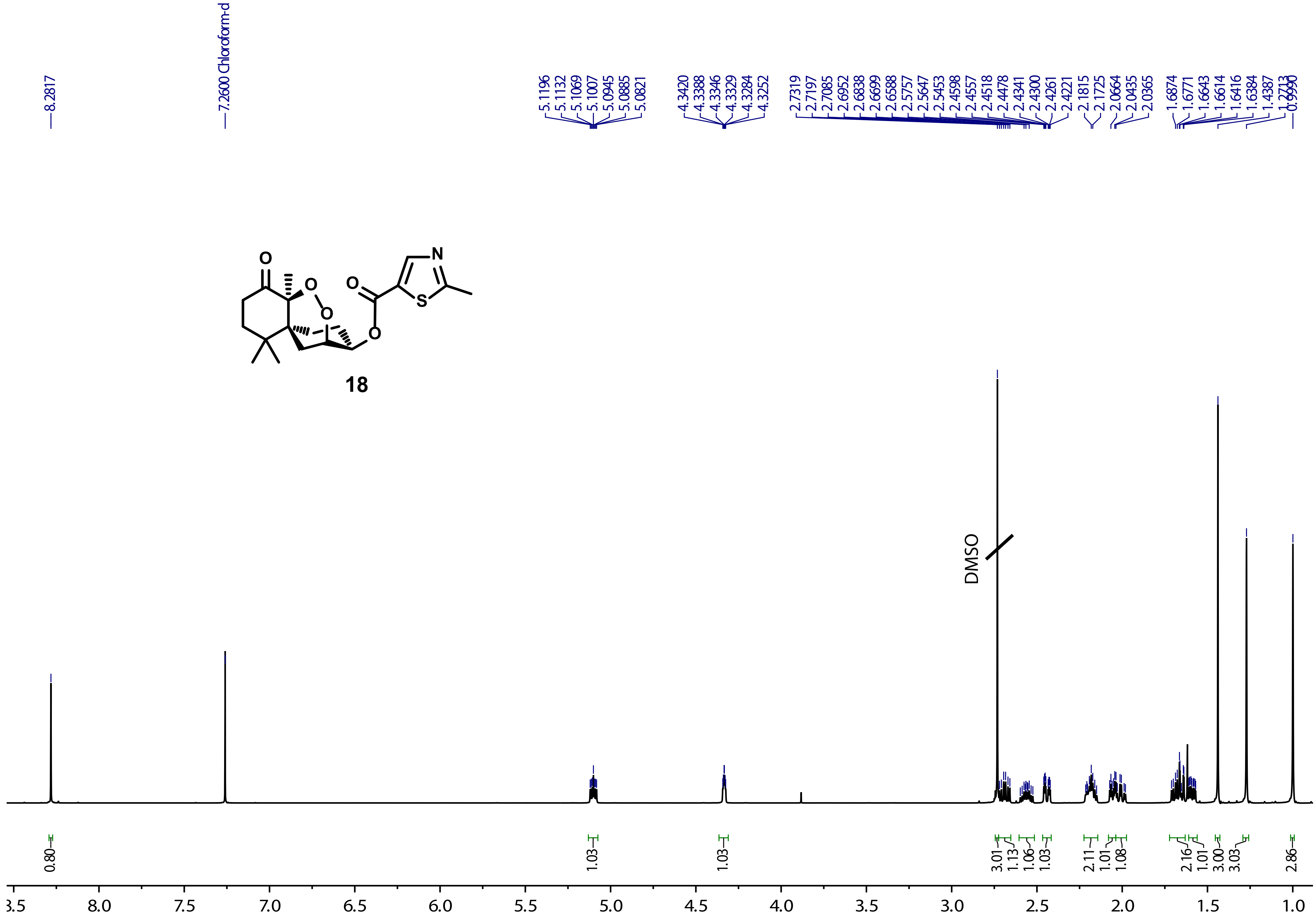
8.2817
7.2600 Chloroform-d
5.1196
5.1132
5.1069
5.1007
5.0945
5.0885
5.0821
4.3420
4.3388
4.3346
4.3329
4.3284
4.3252
2.7319
2.7197
2.7085
2.6952
2.6838
2.6699
2.6588
2.5757
2.5647
2.5453
2.4598
2.4557
2.4518
2.4478
2.4341
2.4300
2.4261
2.4221
2.1815
2.1725
2.0664
2.0435
2.0365
1.6874
1.6771
1.6643
1.6614
1.6416
1.6384
1.4387
1.2713
0.9990
O
O
O
O
O
N
S
18
DMSO
0.80
1.03
1.03
3.01
1.13
1.06
1.03
2.11
1.01
1.08
2.16
1.01
3.00
3.03
2.86
8.0
7.5
7.0
6.5
6.0
5.5
5.0
4.5
4.0
3.5
3.0
2.5
2.0
1.5
1.0

Current Chromatogram(s)

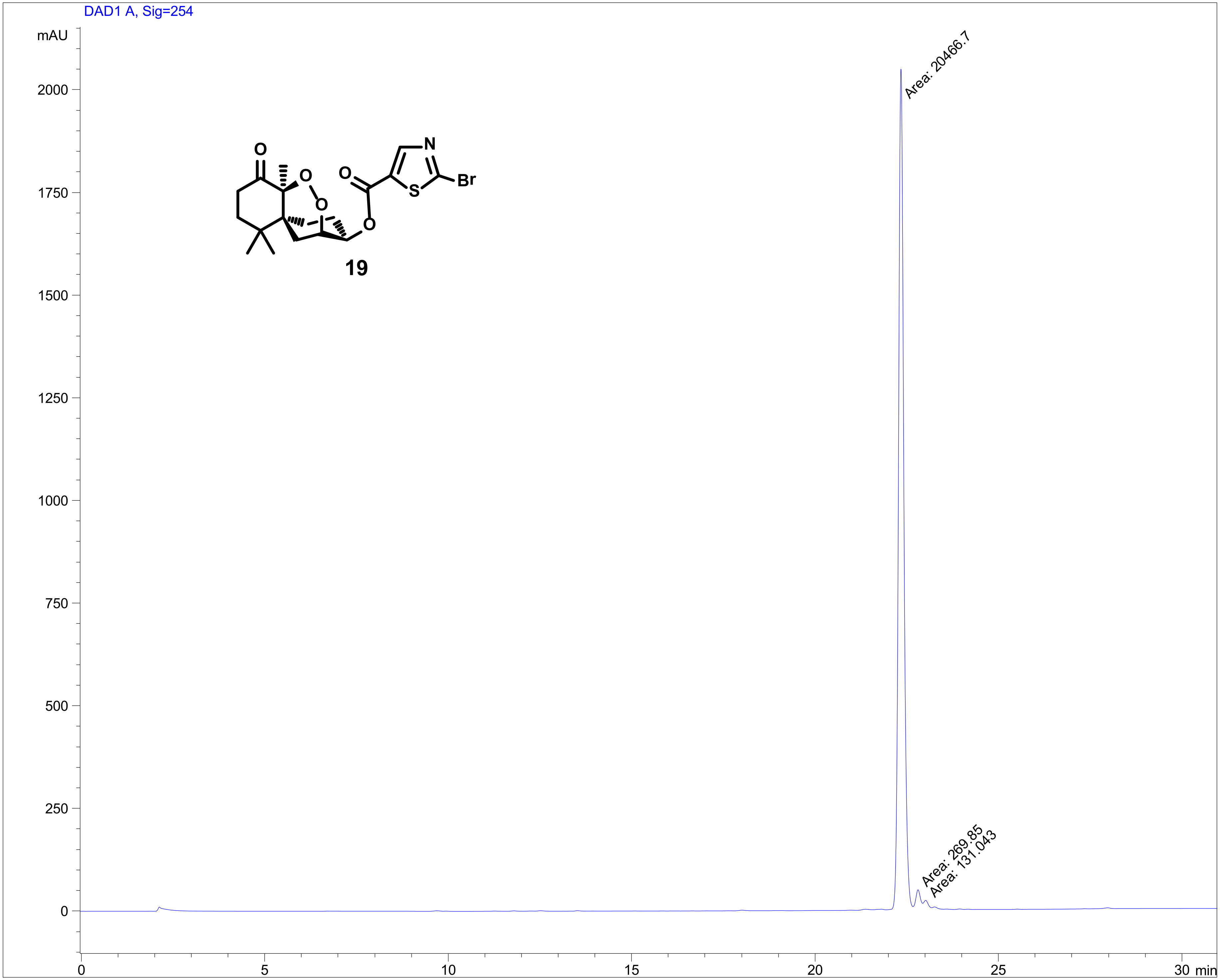

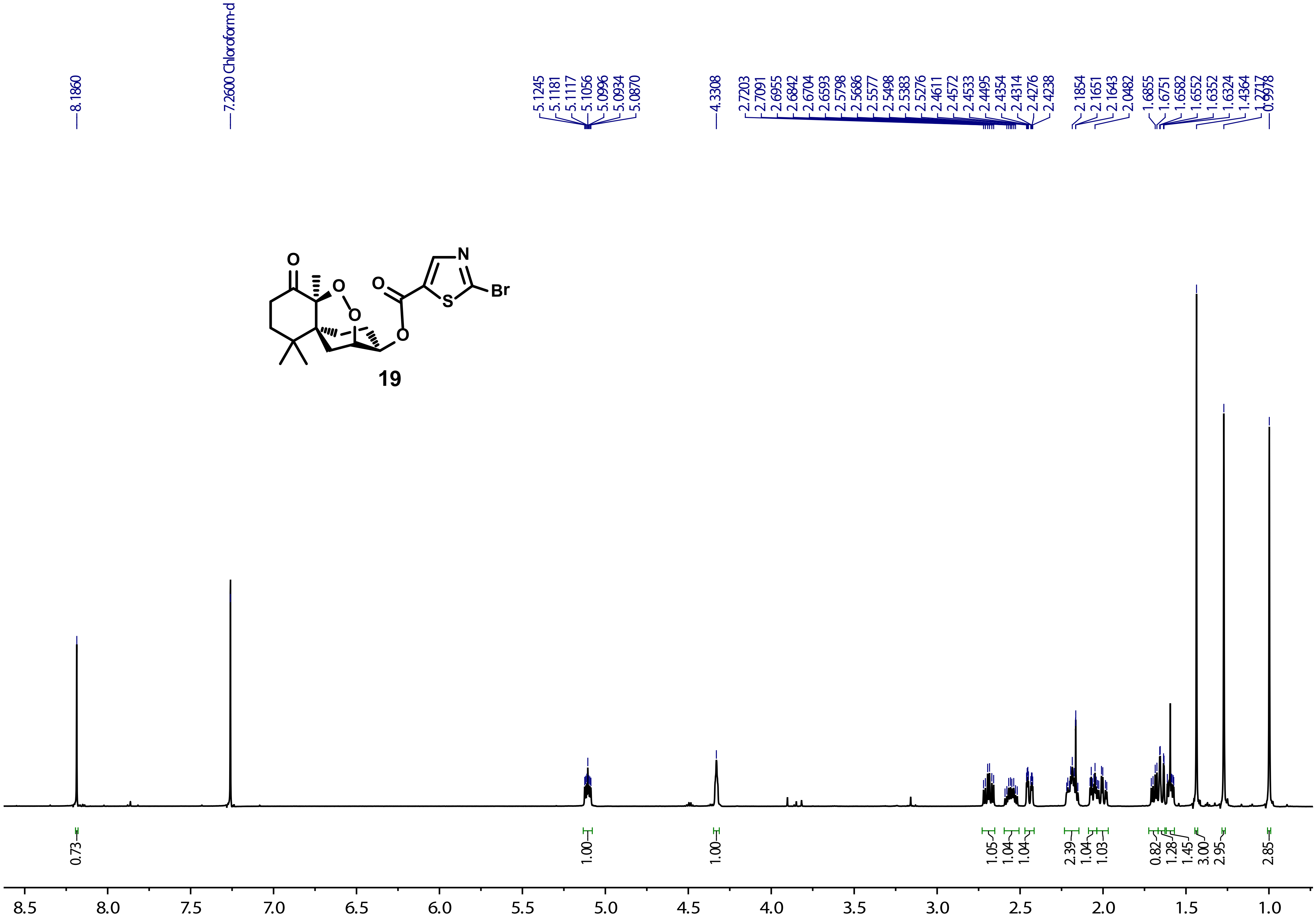
8.1860
7.2600 Chloroform-d
5.1245
5.1181
5.1117
5.1056
5.0996
5.0934
5.0870
4.3308
2.7203
2.7091
2.6955
2.6842
2.6704
2.6593
2.5798
2.5686
2.5577
2.5498
2.5383
2.5276
2.4611
2.4572
2.4533
2.4495
2.4354
2.4314
2.4276
2.4238
2.1854
2.1651
2.1643
2.0482
1.6855
1.6751
1.6582
1.6552
1.6352
1.6324
1.4364
1.2717
0.9978
O
O
O
O
O
N
S
Br
19
0.73
1.00
1.00
1.05
1.04
1.04
2.39
1.04
1.03
0.82
1.28
1.45
3.00
2.95
2.85
8.5
8.0
7.5
7.0
6.5
6.0
5.5
5.0
4.5
4.0
3.5
3.0
2.5
2.0
1.5
1.0

Current Chromatogram(s)

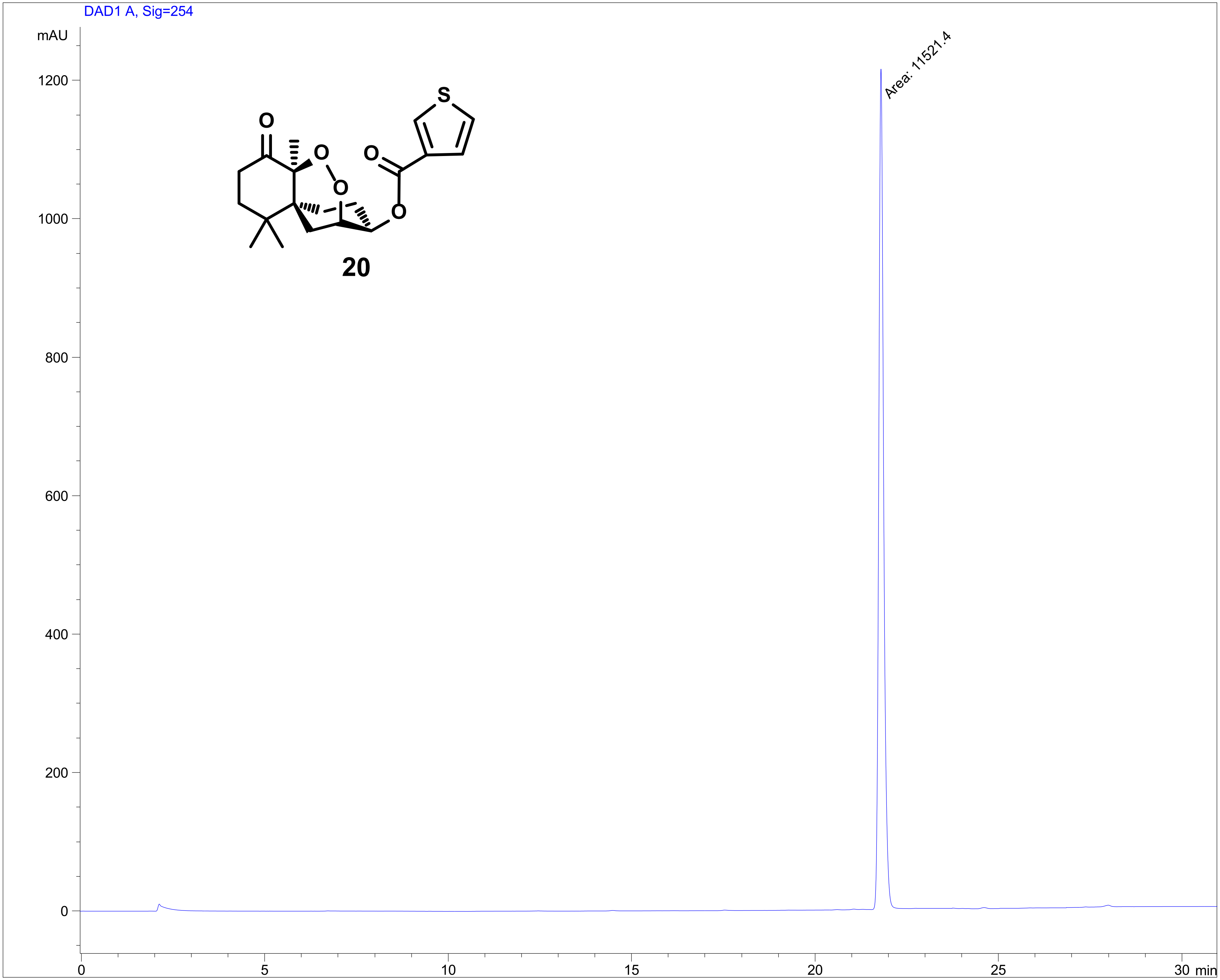

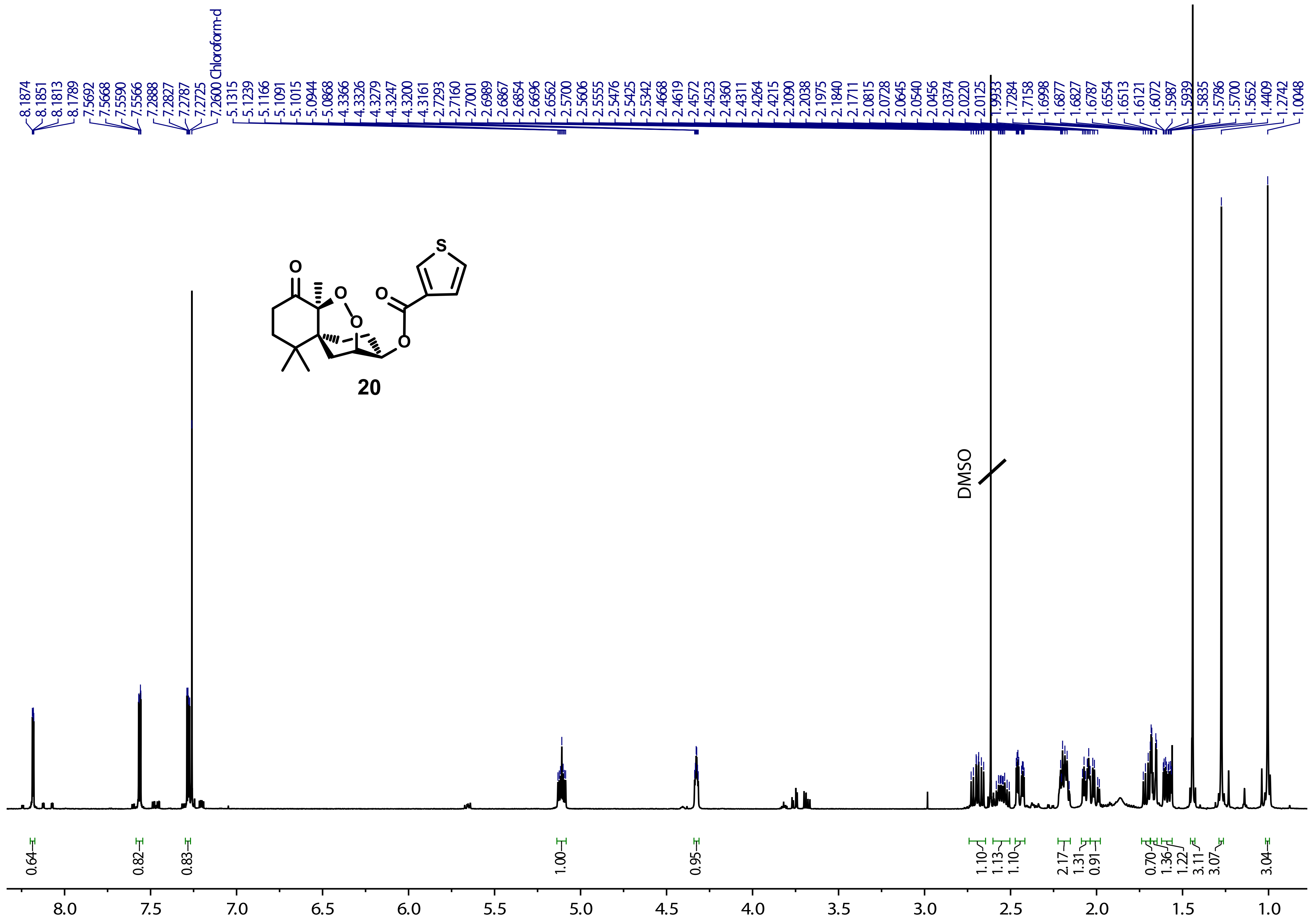

Current Chromatogram(s)

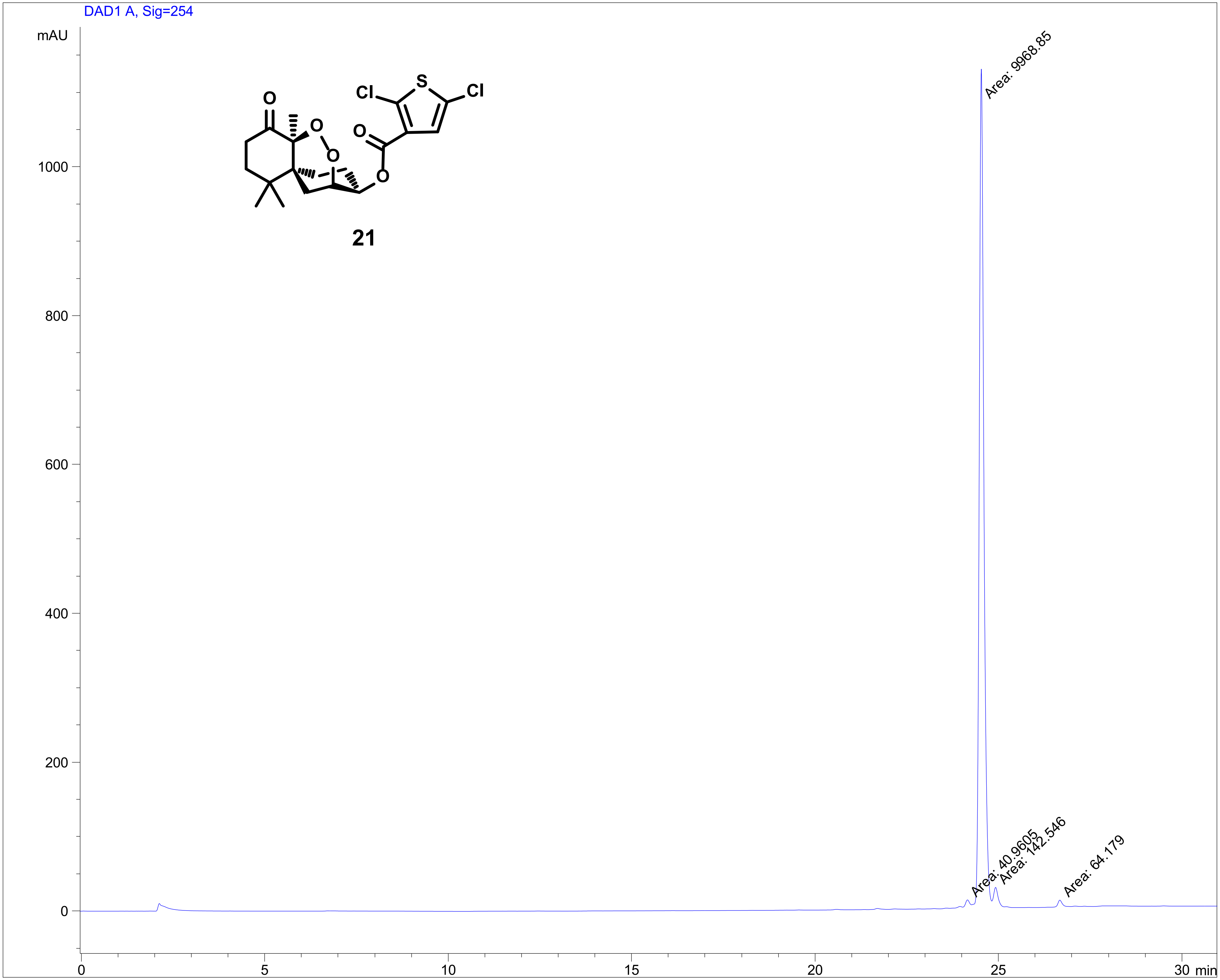

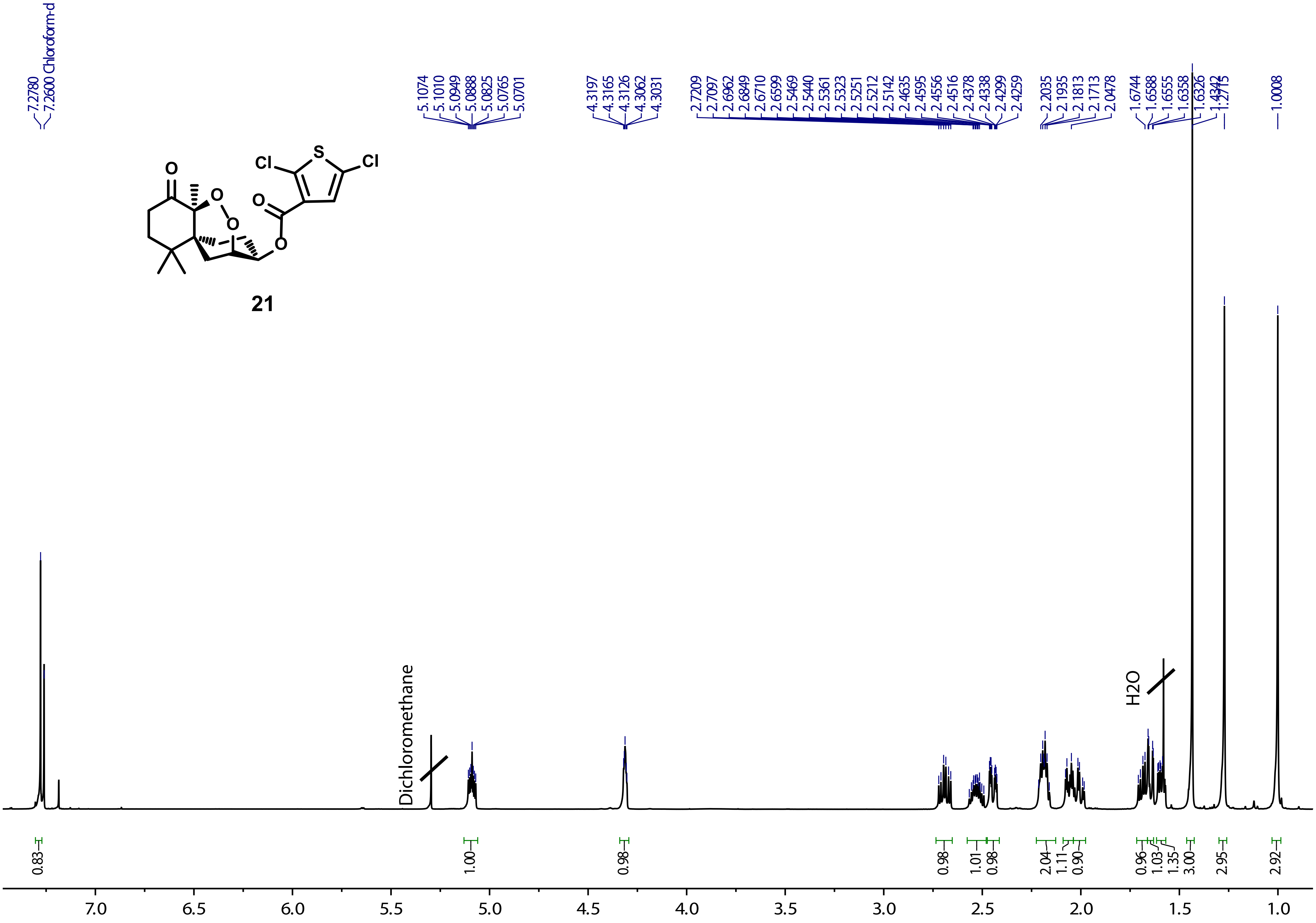
7.2780
7.2600 Chloroform-d
5.1074
5.1010
5.0949
5.0888
5.0825
5.0765
5.0701
4.3197
4.3165
4.3126
4.3062
4.3031
2.7209
2.7097
2.6962
2.6849
2.6710
2.6599
2.5469
2.5440
2.5361
2.5323
2.5251
2.5212
2.5142
2.4635
2.4595
2.4556
2.4516
2.4378
2.4338
2.4299
2.4259
2.2035
2.1935
2.1813
2.1713
2.0478
1.6744
1.6588
1.6555
1.6358
1.6326
1.4342
1.2715
1.0008
O
Cl
S
Cl
O
O
O
O
21
Dichloromethane
H2O
0.83
1.00
0.98
0.98
1.01
0.98
2.04
1.11
0.90
0.96
1.03
1.35
3.00
2.95
2.92
7.0
6.5
6.0
5.5
5.0
4.5
4.0
3.5
3.0
2.5
2.0
1.5
1.0

Current Chromatogram(s)

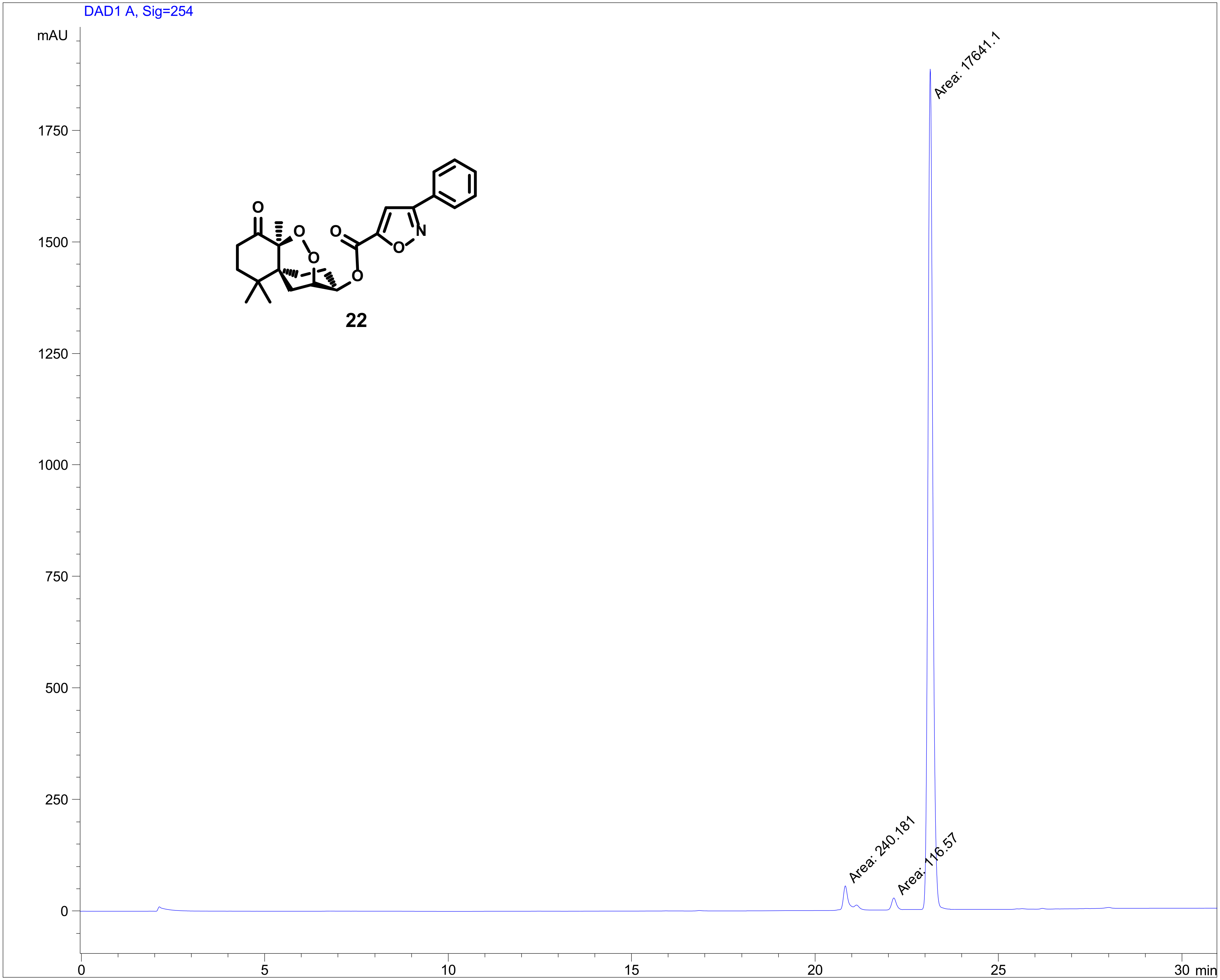

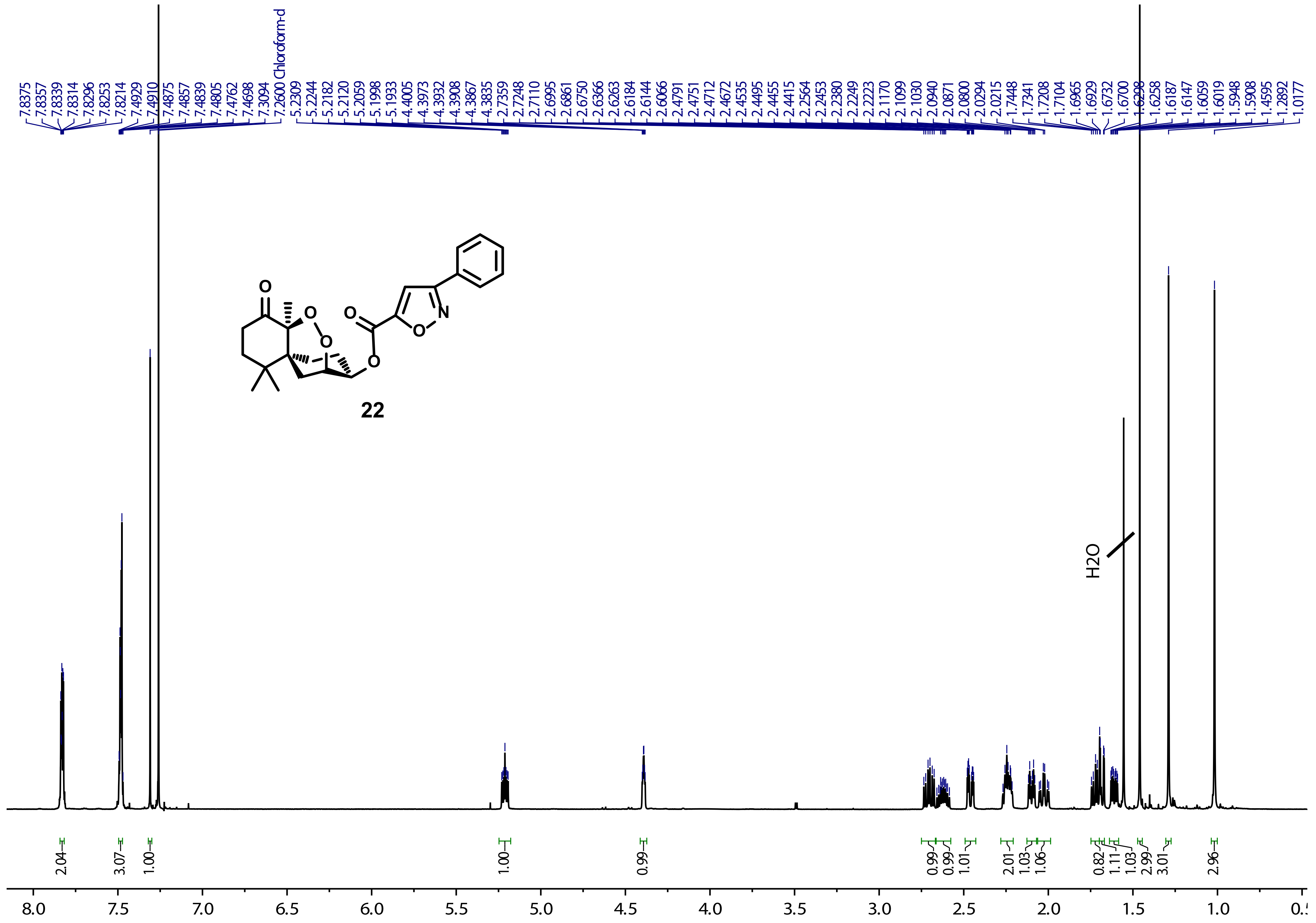

7.8375
7.8357
7.8339
7.8314
7.8296
7.8253
7.8214
7.4929
7.4910
7.4875
7.4857
7.4839
7.4805
7.4762
7.4698
7.3094
7.2600 Chloroform-d
5.2309
5.2244
5.2182
5.2120
5.2059
5.1998
5.1933
4.4005
4.3973
4.3932
4.3908
4.3867
4.3835
2.7359
2.7248
2.7110
2.6995
2.6861
2.6750
2.6366
2.6263
2.6184
2.6144
2.6066
2.4791
2.4751
2.4712
2.4672
2.4535
2.4495
2.4455
2.4415
2.2564
2.2453
2.2380
2.2249
2.2223
2.1170
2.1099
2.1030
2.0940
2.0871
2.0800
2.0294
2.0215
1.7448
1.7341
1.7208
1.7104
1.6965
1.6929
1.6732
1.6700
1.6298
1.6258
1.6187
1.6147
1.6059
1.6019
1.5948
1.5908
1.4595
1.2892
1.0177
22
H2O
2.04
3.07
1.00
1.00
0.99
0.99
0.99
1.01
2.01
1.03
1.06
0.82
1.11
1.03
2.99
3.01
2.96
8.0
7.5
7.0
6.5
6.0
5.5
5.0
4.5
4.0
3.5
3.0
2.5
2.0
1.5
1.0

Current Chromatogram(s)

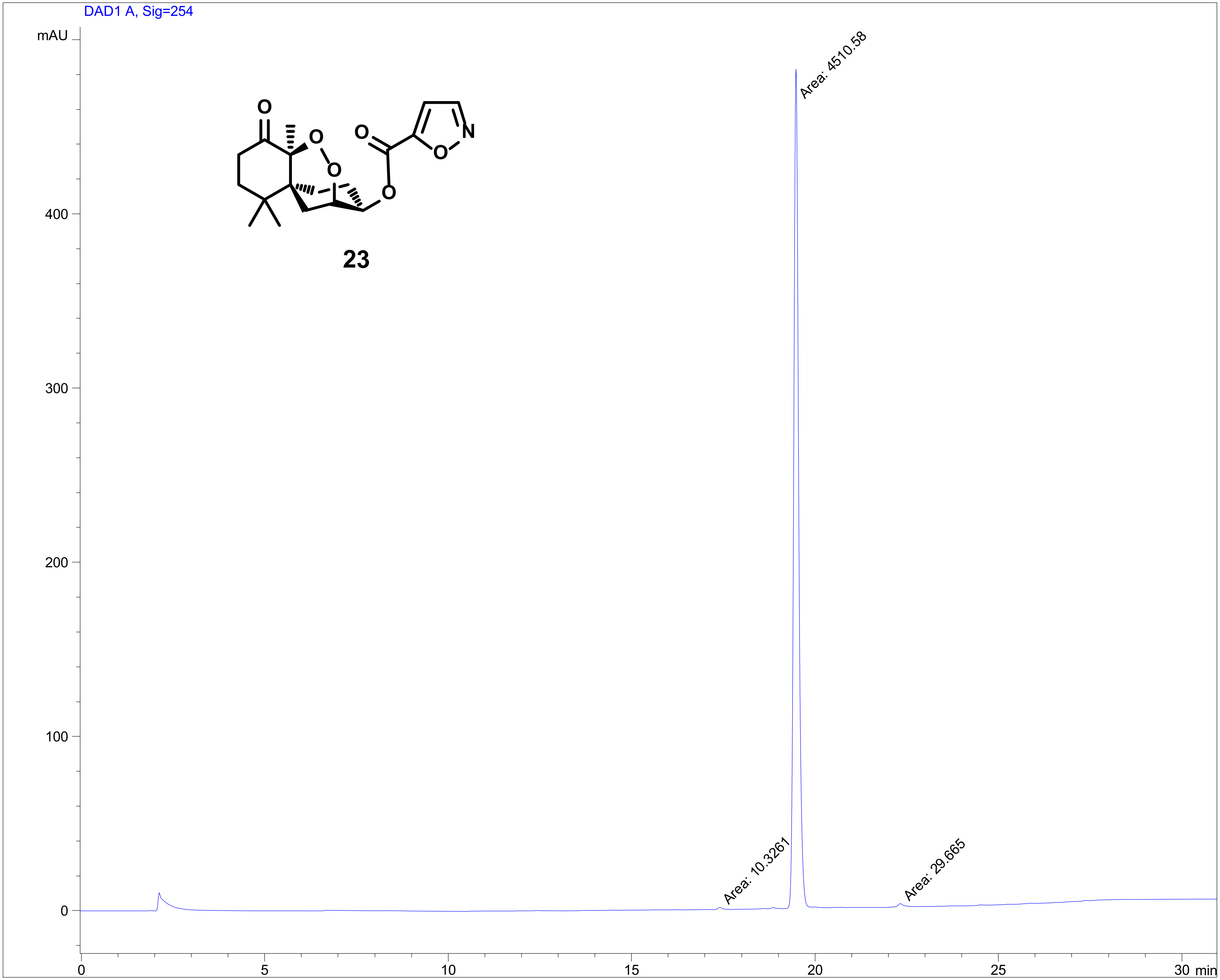

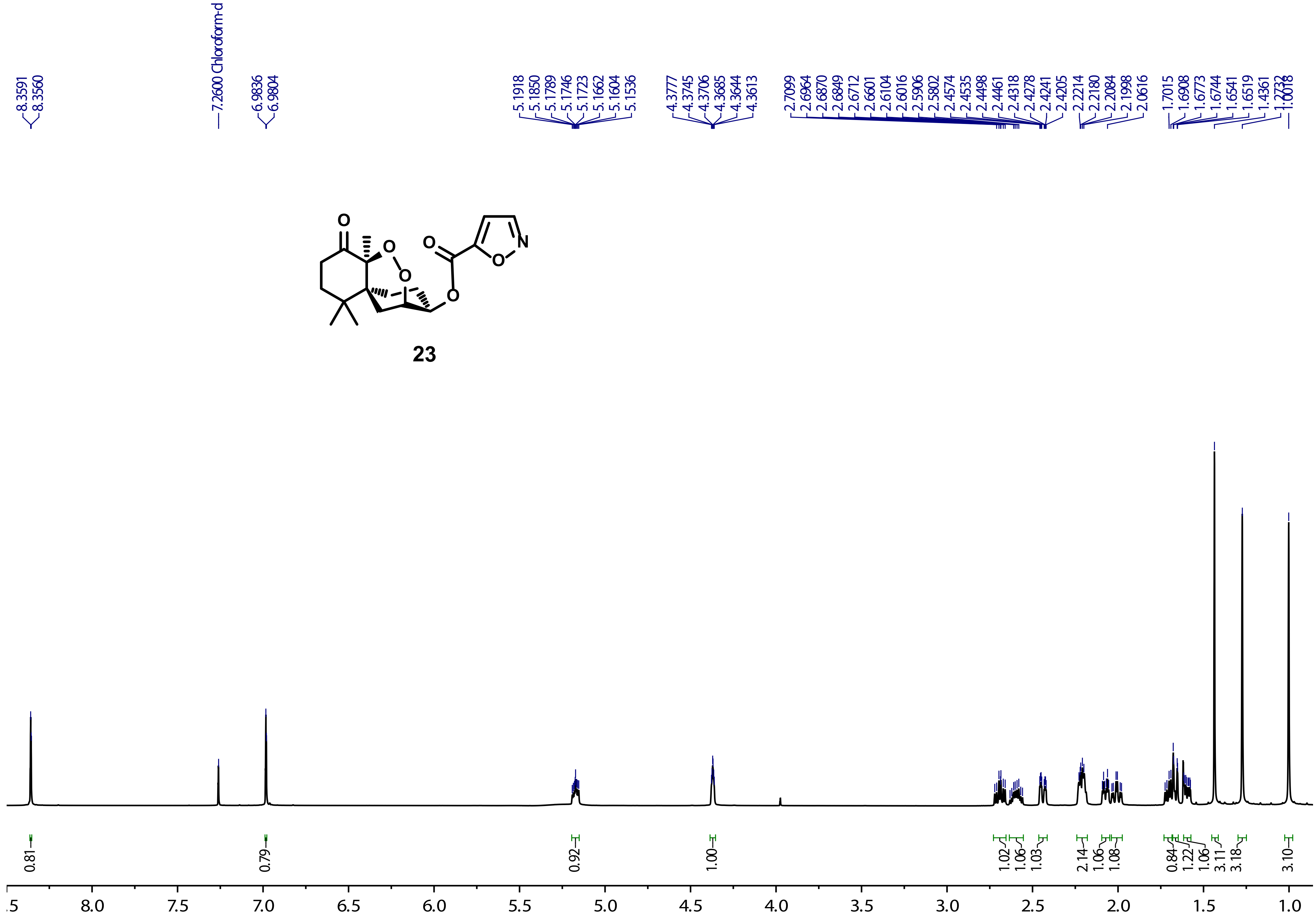
8.3591
8.3560
7.2600 Chloroform-d
6.9836
6.9804
5.1918
5.1850
5.1789
5.1746
5.1723
5.1662
5.1604
5.1536
4.3777
4.3745
4.3706
4.3685
4.3644
4.3613
2.7099
2.6964
2.6870
2.6849
2.6712
2.6601
2.6104
2.6016
2.5906
2.5802
2.4574
2.4535
2.4498
2.4461
2.4318
2.4278
2.4241
2.4205
2.2214
2.2180
2.2084
2.1998
2.0616
1.7015
1.6908
1.6773
1.6744
1.6541
1.6519
1.4361
1.2732
1.0018
O
O
O
O
O
O
N
23
0.81
0.79
0.92
1.00
1.02
1.06
1.03
2.14
1.06
1.08
0.84
1.22
1.06
3.11
3.18
3.10
.5
8.0
7.5
7.0
6.5
6.0
5.5
5.0
4.5
4.0
3.5
3.0
2.5
2.0
1.5
1.0

Current Chromatogram(s)

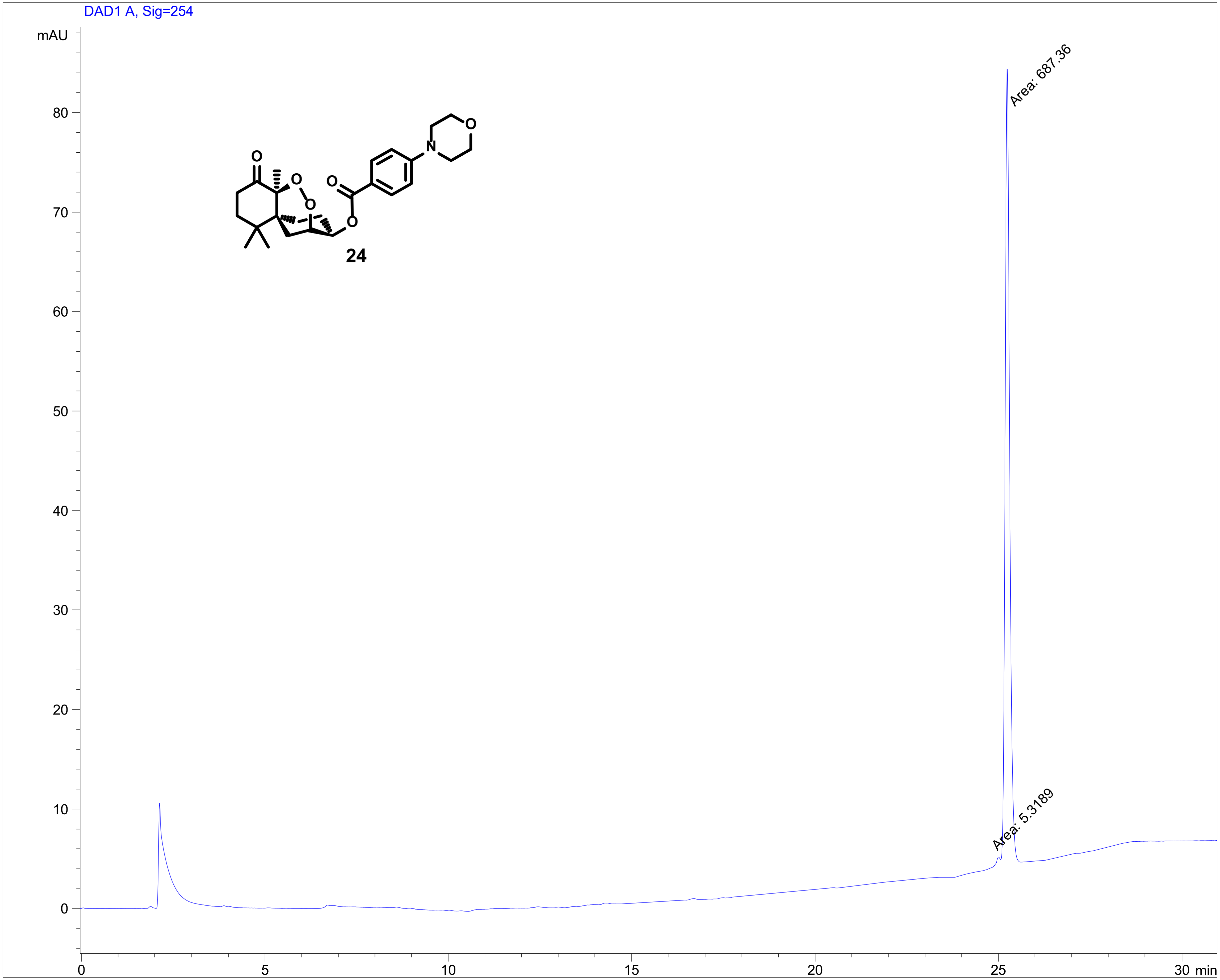

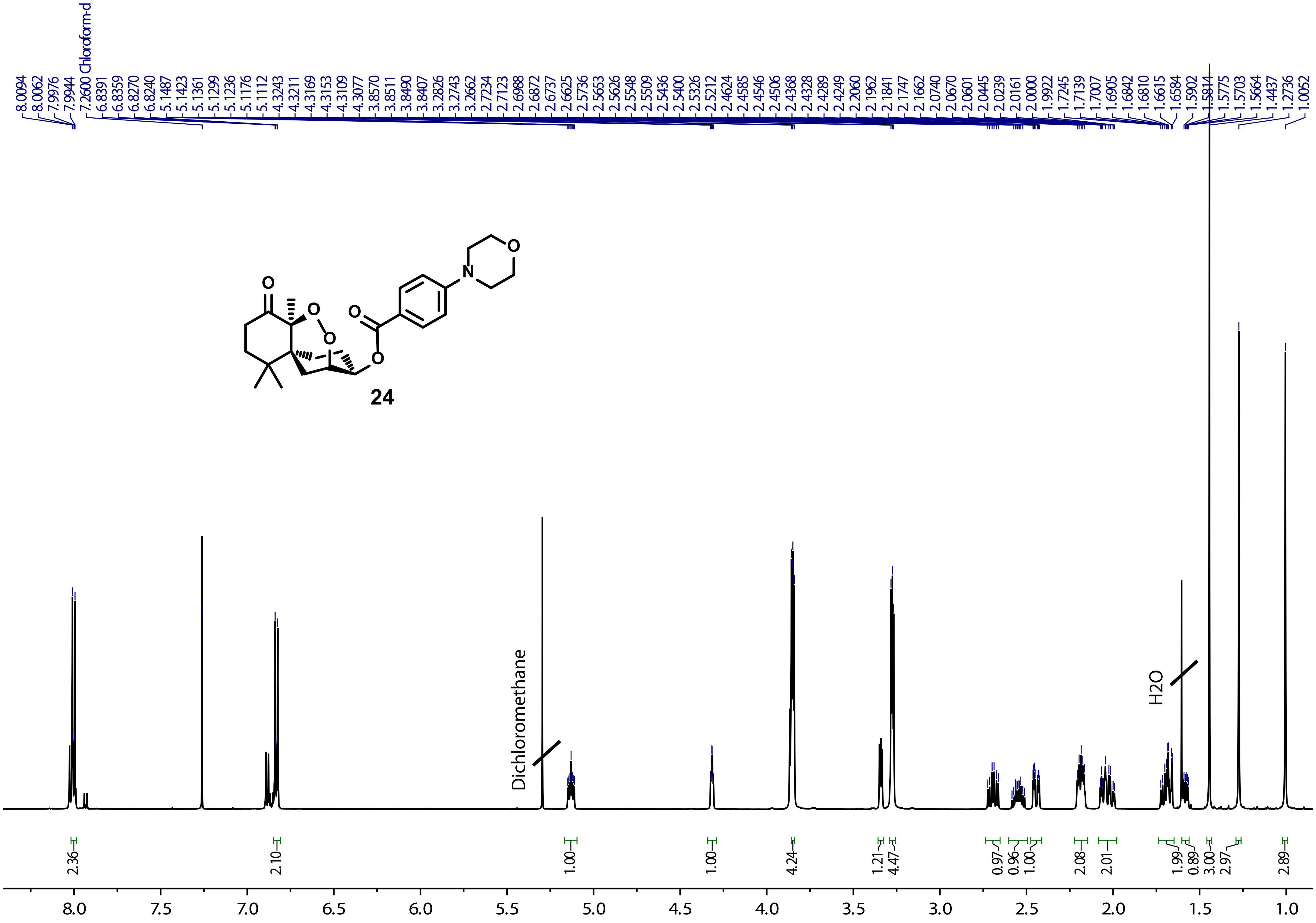
8.0094
8.0062
7.9976
7.9944
7.2600 Chloroform-d
6.8391
6.8359
6.8270
6.8240
5.1487
5.1423
5.1361
5.1299
5.1236
5.1176
5.1112
4.3243
4.3211
4.3169
4.3153
4.3109
4.3077
3.8570
3.8511
3.8490
3.8407
3.2826
3.2743
3.2662
2.7234
2.7123
2.6988
2.6872
2.6737
2.6625
2.5736
2.5653
2.5626
2.5548
2.5509
2.5436
2.5400
2.5326
2.5212
2.4624
2.4585
2.4546
2.4506
2.4368
2.4328
2.4289
2.4249
2.2060
2.1962
2.1841
2.1747
2.1662
2.0740
2.0670
2.0601
2.0445
2.0239
2.0161
2.0000
1.9922
1.7245
1.7139
1.7007
1.6905
1.6842
1.6810
1.6615
1.6584
1.5902
1.5814
1.5775
1.5703
1.5664
1.4437
1.2736
1.0052
O
O
O
O
O
N
O
O
24
Dichloromethane
H2O
2.36
2.10
1.00
1.00
4.24
1.21
4.47
0.97
0.96
1.00
2.08
2.01
1.99
0.89
3.00
2.97
2.89
8.0
7.5
7.0
6.5
6.0
5.5
5.0
4.5
4.0
3.5
3.0
2.5
2.0
1.5
1.0

Current Chromatogram(s)

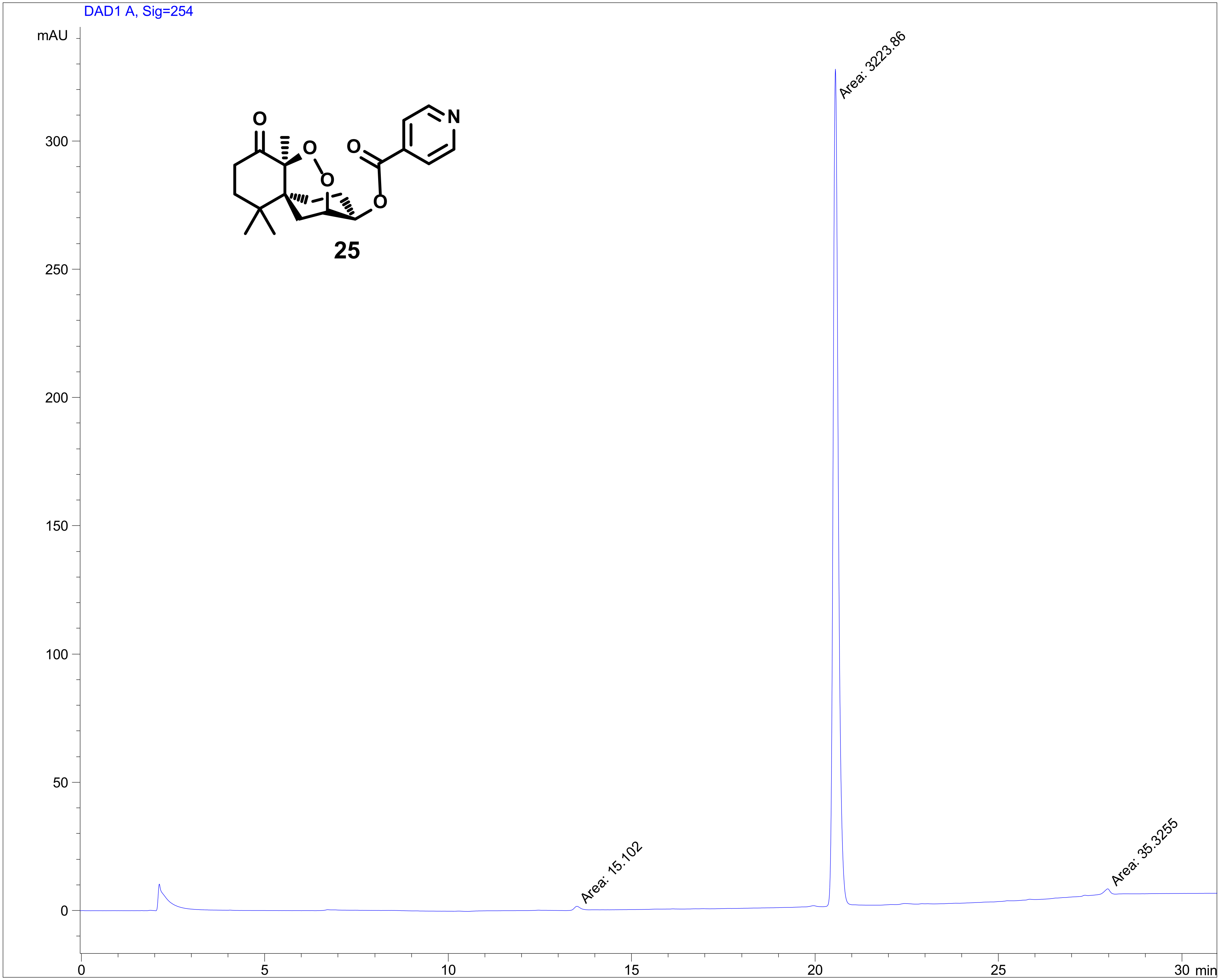

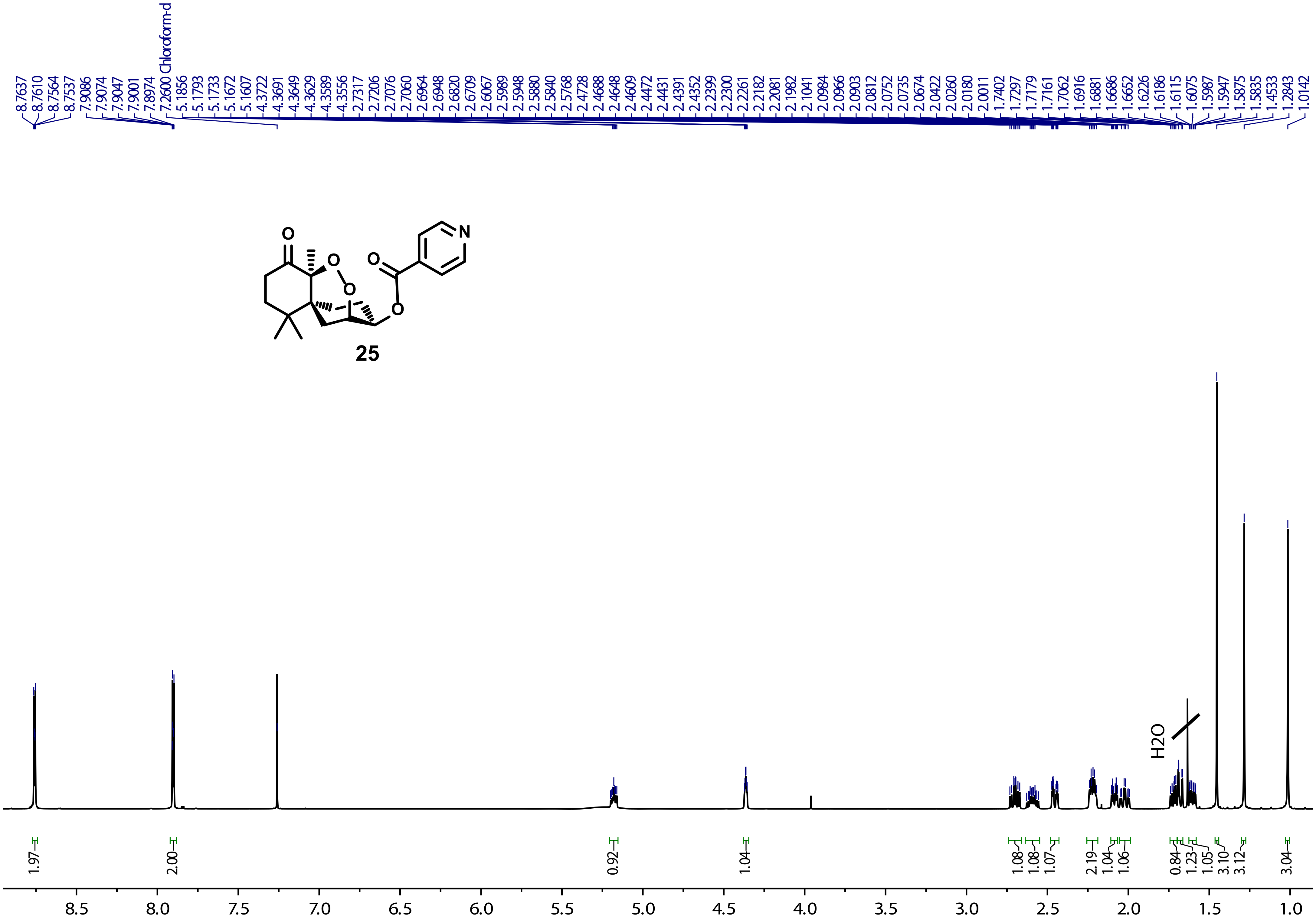
8.7637 8.7610 8.7564 8.7537 7.9086 7.9074 7.9047 7.9001 7.8974 7.2600 Chloroform-d 5.1856 5.1793 5.1733 5.1672 5.1607 4.3722 4.3691 4.3649 4.3629 4.3589 4.3556 2.7317 2.7206 2.7076 2.7060 2.6964 2.6948 2.6820 2.6709 2.6067 2.5989 2.5948 2.5880 2.5840 2.5768 2.4728 2.4688 2.4648 2.4609 2.4472 2.4431 2.4391 2.4352 2.2399 2.2300 2.2261 2.2182 2.2081 2.1982 2.1041 2.0984 2.0966 2.0903 2.0812 2.0752 2.0735 2.0674 2.0422 2.0260 2.0180 2.0011 1.7402 1.7297 1.7179 1.7161 1.7062 1.6916 1.6881 1.6686 1.6652 1.6226 1.6186 1.6115 1.6075 1.5987 1.5947 1.5875 1.5835 1.4533 1.2843 1.0142
O
O
O
O
O
N
25
H2O
1.97 2.00 0.92 1.04 1.08 1.08 1.07 2.19 1.04 1.06 0.84 1.23 1.05 3.10 3.12 3.04
8.5 8.0 7.5 7.0 6.5 6.0 5.5 5.0 4.5 4.0 3.5 3.0 2.5 2.0 1.5 1.0

Current Chromatogram(s)

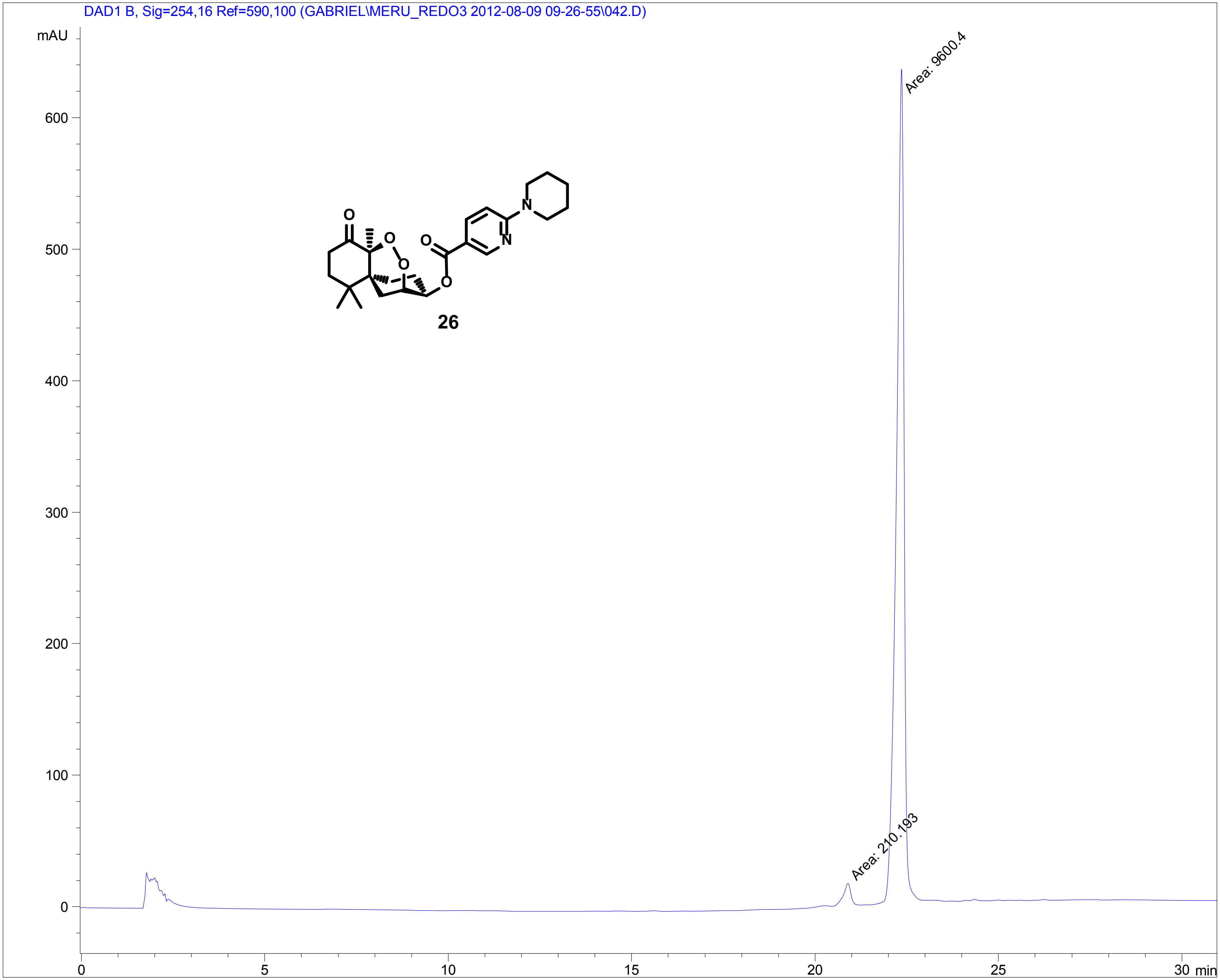

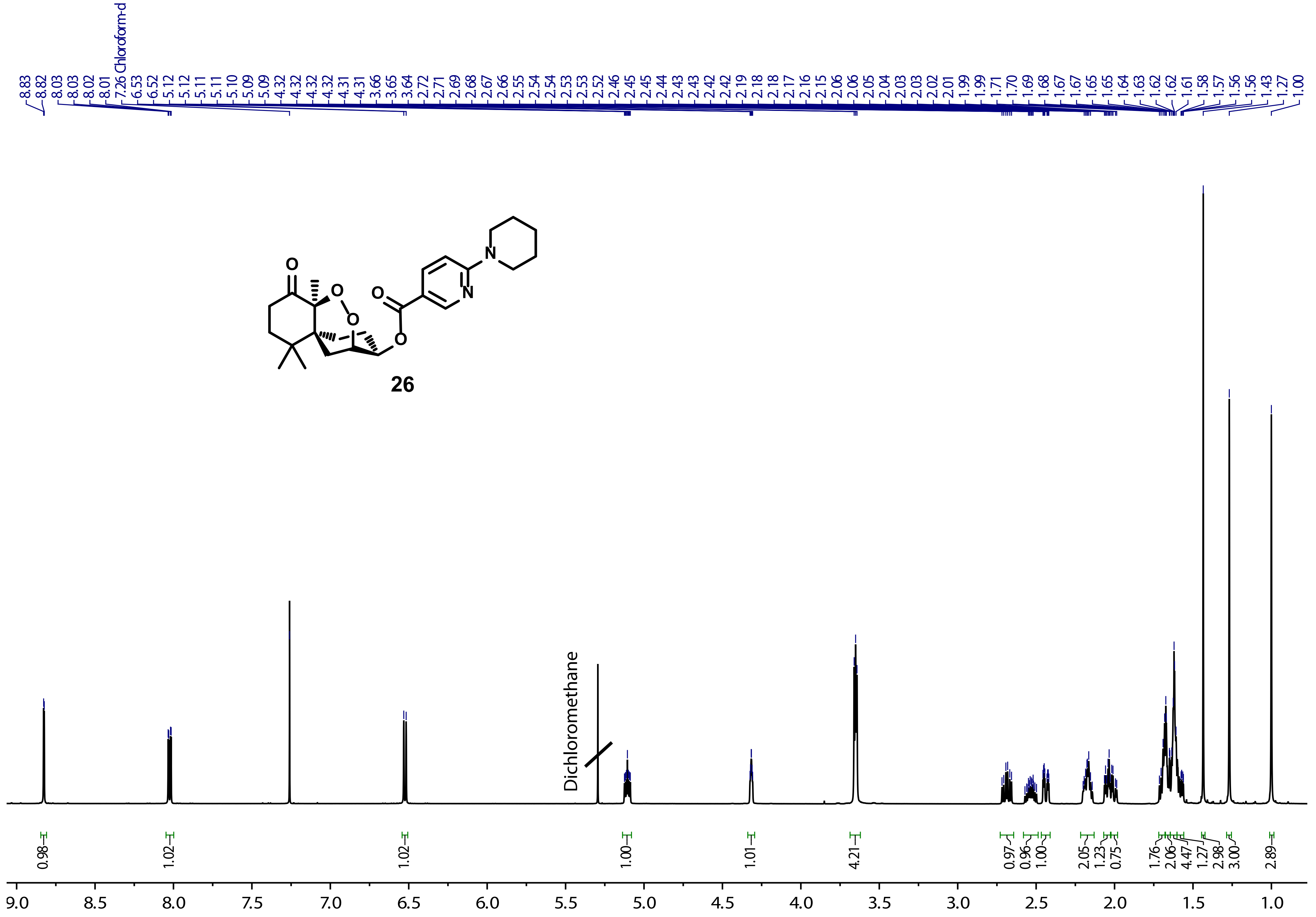
8.83
8.82
8.03
8.03
8.02
8.01
7.26 Chloroform-d
6.53
6.52
5.12
5.12
5.11
5.11
5.10
5.09
5.09
4.32
4.32
4.32
4.32
4.31
4.31
3.66
3.65
3.64
2.72
2.71
2.69
2.68
2.67
2.66
2.55
2.54
2.54
2.53
2.53
2.52
2.46
2.45
2.45
2.44
2.43
2.43
2.42
2.42
2.19
2.18
2.18
2.17
2.16
2.15
2.06
2.06
2.05
2.04
2.03
2.03
2.02
2.01
1.99
1.99
1.71
1.70
1.69
1.68
1.67
1.67
1.65
1.65
1.64
1.63
1.62
1.62
1.61
1.58
1.57
1.56
1.56
1.43
1.27
1.00
O
O
O
O
O
N
N
26
Dichloromethane
0.98
1.02
1.02
1.00
1.01
4.21
0.97
0.96
1.00
2.05
1.23
0.75
1.76
2.06
4.47
1.27
2.98
3.00
2.89
9.0
8.5
8.0
7.5
7.0
6.5
6.0
5.5
5.0
4.5
4.0
3.5
3.0
2.5
2.0
1.5
1.0

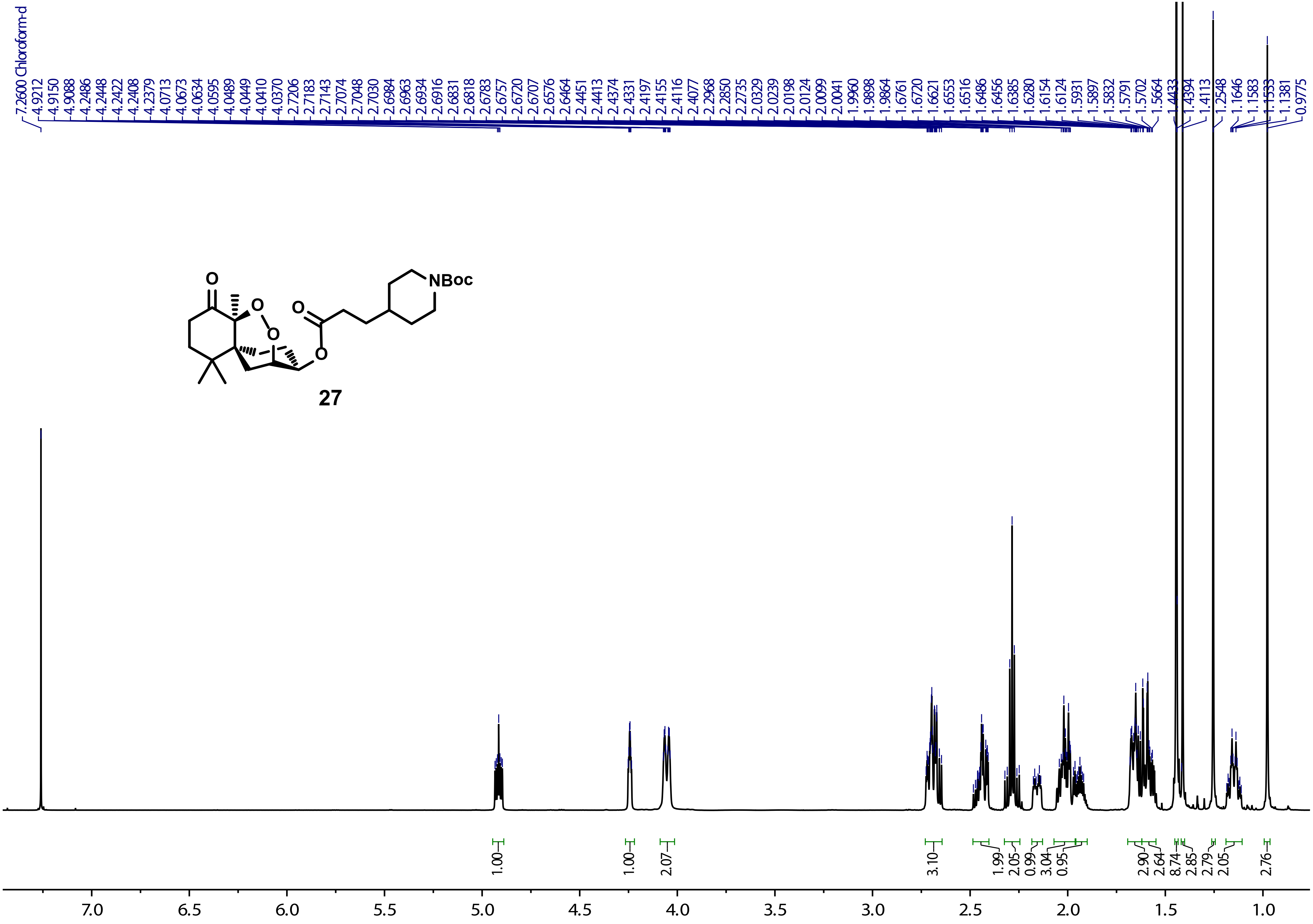
7.2600 Chloroform-d
4.9212
4.9150
4.9088
4.2486
4.2448
4.2422
4.2408
4.2379
4.0713
4.0673
4.0634
4.0595
4.0489
4.0449
4.0410
4.0370
2.7206
2.7183
2.7143
2.7074
2.7048
2.7030
2.6984
2.6963
2.6934
2.6916
2.6831
2.6818
2.6783
2.6757
2.6720
2.6707
2.6576
2.6464
2.6451
2.4413
2.4374
2.4331
2.4197
2.4155
2.4116
2.4077
2.2968
2.2850
2.2735
2.0329
2.0239
2.0198
2.0124
2.0099
2.0041
1.9960
1.9898
1.9864
1.6761
1.6720
1.6621
1.6553
1.6516
1.6486
1.6456
1.6385
1.6280
1.6154
1.6124
1.5931
1.5897
1.5832
1.5791
1.5702
1.5664
1.4433
1.4394
1.4113
1.2548
1.1646
1.1583
1.1533
1.1381
0.9775
O
O
O
O
O
NBoc
27
1.00
1.00
2.07
3.10
1.99
2.05
0.99
3.04
0.95
2.90
2.64
8.74
2.85
2.79
2.05
2.76
7.0
6.5
6.0
5.5
5.0
4.5
4.0
3.5
3.0
2.5
2.0
1.5
1.0

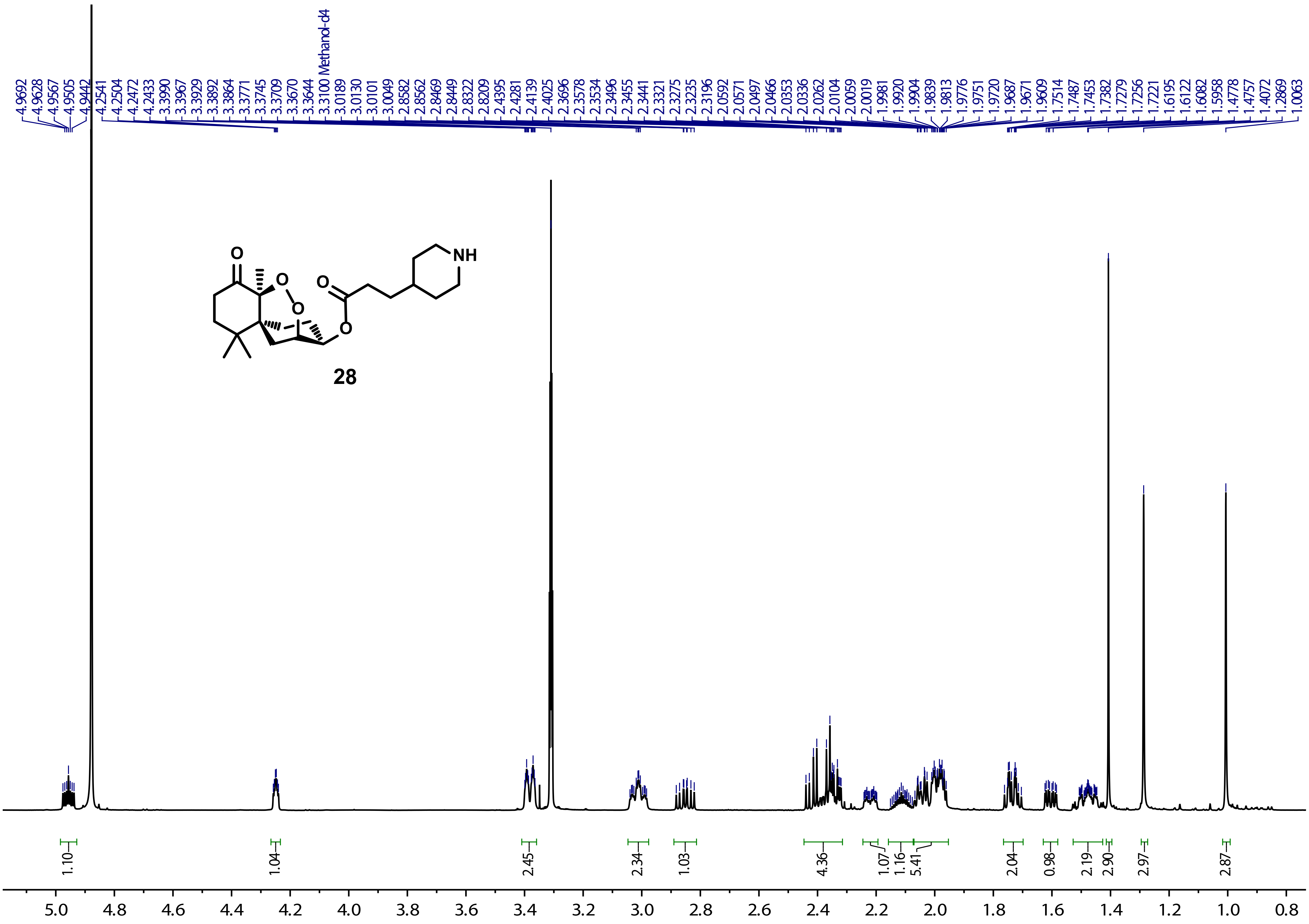
4.9692
4.9628
4.9567
4.9505
4.9442
4.2541
4.2504
4.2472
4.2433
3.3990
3.3967
3.3929
3.3892
3.3864
3.3771
3.3745
3.3709
3.3670
3.3644
Methanol-d4
3.3100
3.0189
3.0130
3.0101
3.0049
2.8582
2.8562
2.8469
2.8449
2.8322
2.8209
2.4395
2.4281
2.4139
2.4025
2.3696
2.3578
2.3534
2.3496
2.3455
2.3441
2.3321
2.3275
2.3235
2.3196
2.0592
2.0571
2.0497
2.0466
2.0353
2.0336
2.0262
2.0104
2.0059
2.0019
1.9981
1.9920
1.9904
1.9839
1.9813
1.9776
1.9751
1.9720
1.9687
1.9671
1.9609
1.7514
1.7487
1.7453
1.7382
1.7279
1.7256
1.7221
1.6195
1.6122
1.6082
1.5958
1.4778
1.4757
1.4072
1.2869
1.0063
O
O
O
O
NH
O
28
1.10
1.04
2.45
2.34
1.03
4.36
1.07
1.16
5.41
2.04
0.98
2.19
2.90
2.97
2.87
5.0
4.8
4.6
4.4
4.2
4.0
3.8
3.6
3.4
3.2
3.0
2.8
2.6
2.4
2.2
2.0
1.8
1.6
1.4
1.2
1.0
0.8

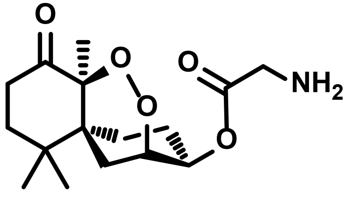
O
O
O
O
NH2
O
O

29

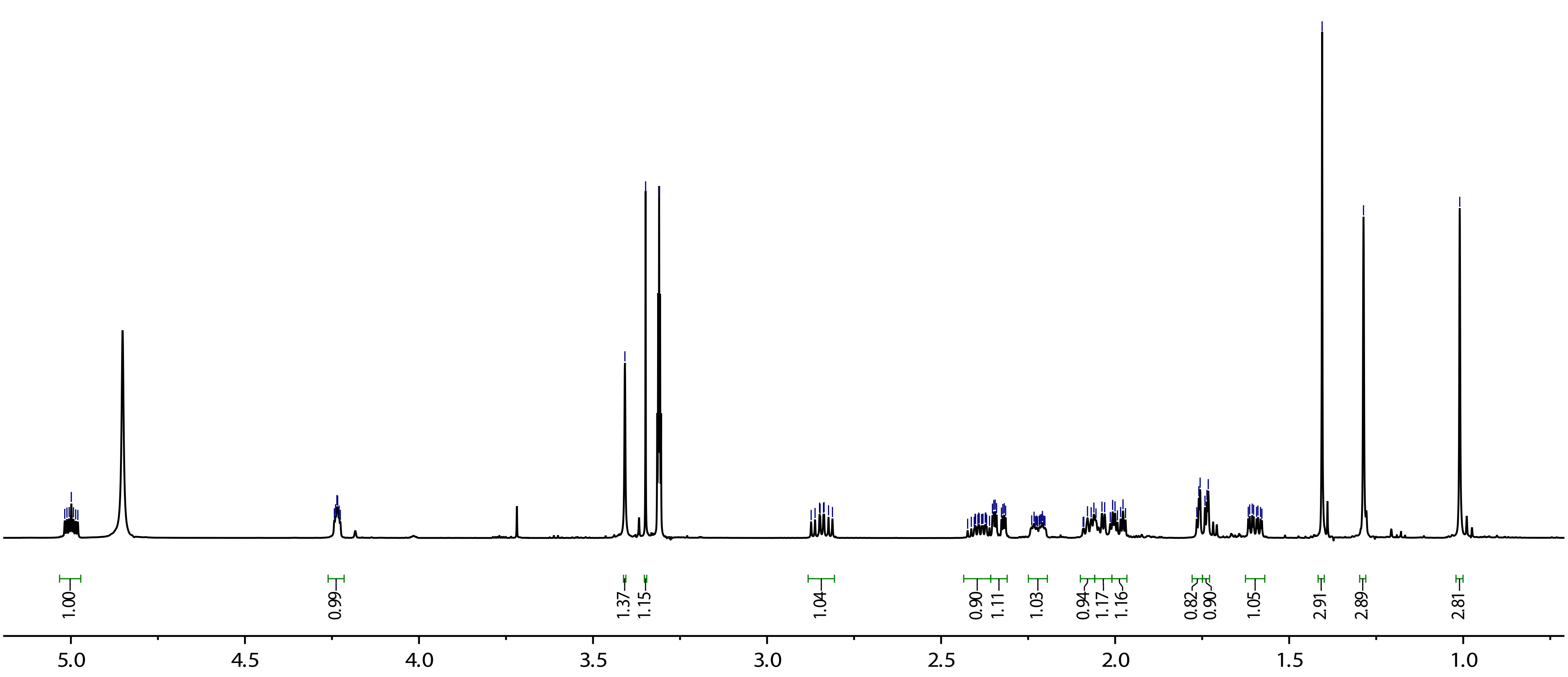
5.02
5.01
5.01
5.00
4.99
4.99
4.98
4.24
4.24
4.24
4.23
4.23
4.23
3.41
3.35
3.31 Methanol-d4
2.87
2.86
2.85
2.85
2.84
2.84
2.82
2.81
2.40
2.40
2.39
2.39
2.39
2.38
2.38
2.38
2.37
2.37
2.37
2.35
2.35
2.34
2.34
2.33
2.32
2.32
2.31
2.24
2.23
2.23
2.22
2.22
2.21
2.21
2.21
2.08
2.07
2.06
2.06
2.04
2.03
2.01
2.01
2.00
1.99
1.98
1.98
1.97
1.77
1.76
1.76
1.74
1.74
1.73
1.62
1.61
1.61
1.60
1.59
1.59
1.58
1.58
1.41
1.29
1.01
1.00
0.99
1.37
1.15
1.04
0.90
1.11
1.03
0.94
1.17
1.16
0.82
0.90
1.05
2.91
2.89
2.81
5.0
4.5
4.0
3.5
3.0
2.5
2.0
1.5
1.0

Current Chromatogram(s)

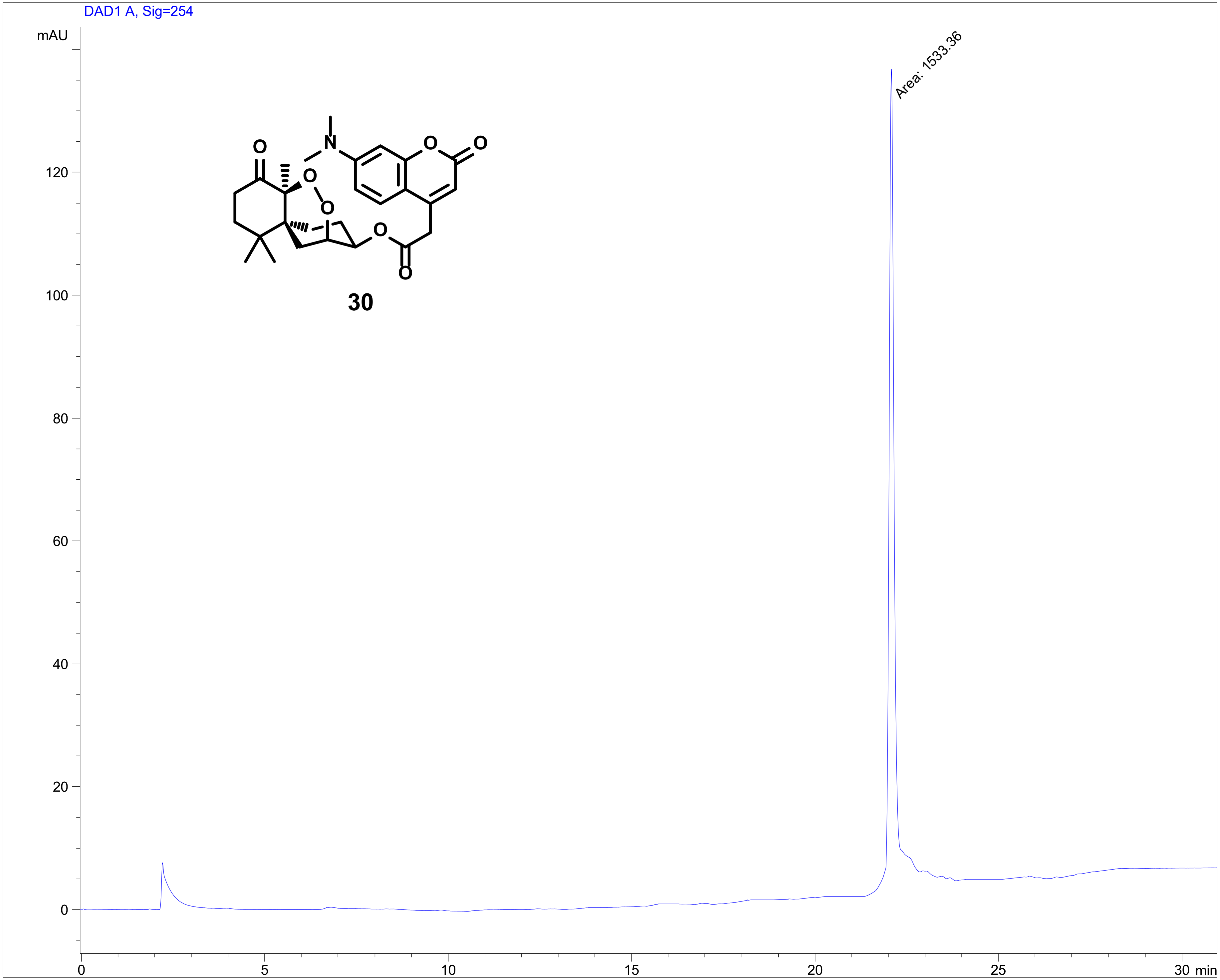

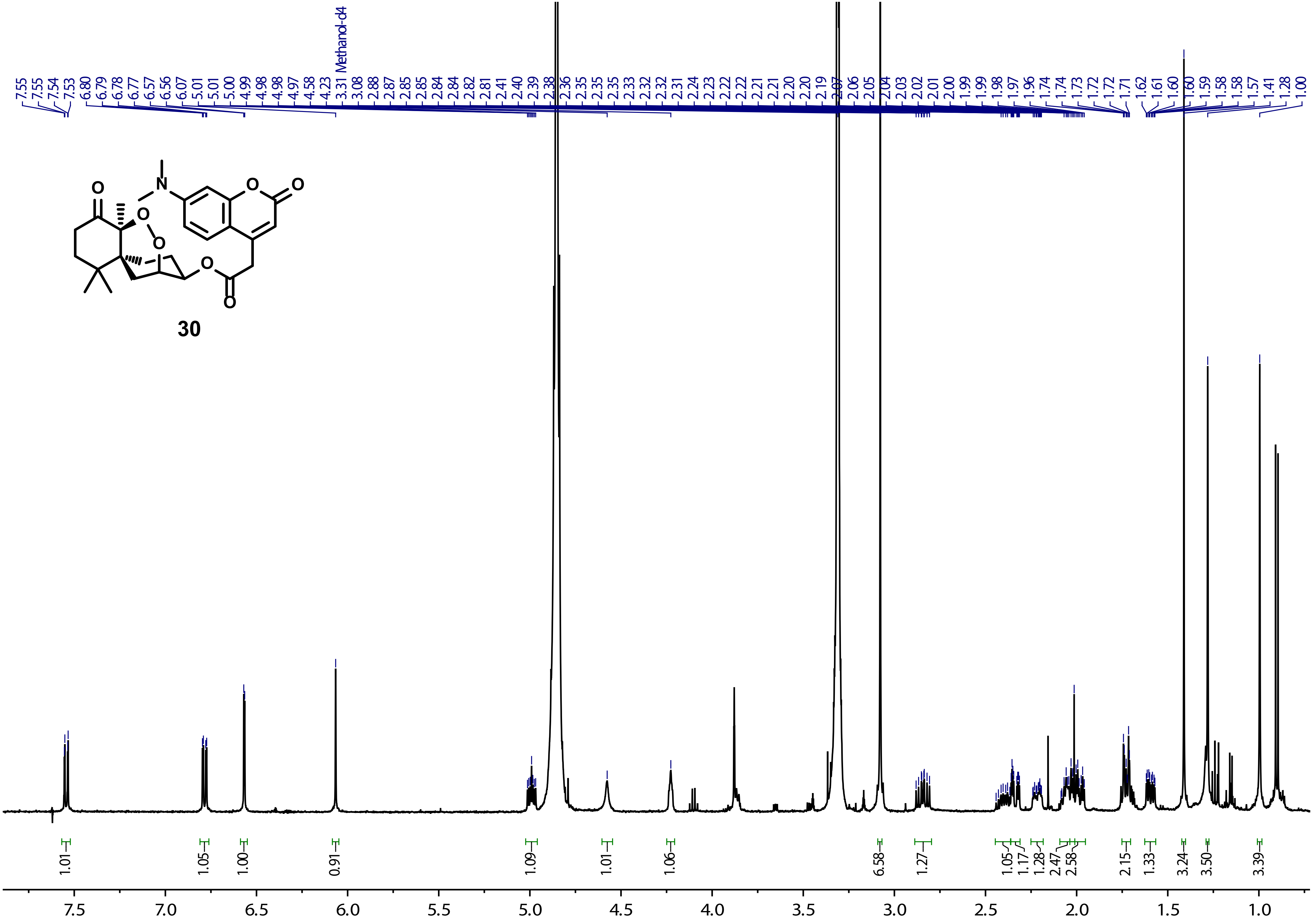

30
